# Plug Flow: Generating Renewable Electricity with Water from Nature by Breaking the Limit of Debye Length

**DOI:** 10.1021/acscentsci.4c02110

**Published:** 2025-04-16

**Authors:** Chi Kit Ao, Yajuan Sun, Yan Jie Neriah Tan, Yan Jiang, Zhenxing Zhang, Chengyu Zhang, Siowling Soh

**Affiliations:** Department of Chemical and Biomolecular Engineering, 37580 National University of Singapore, 4 Engineering Drive 4, Singapore 117585, Singapore

## Abstract

Charge separation occurs spontaneously at the solid–liquid interface, forming an electric double layer. Previous methods, including streaming current, used to harvest the constantly separated charge in micron-sized and larger systems reported negligible power output due to the fundamental limit caused by the very short nanoscale Debye length. This study reports on the phenomenon that plug flow of water that falls naturally down a millimeter-sized tube generates electricity with a high efficiency of >10% and power density of ∼100 W/m^2^. This high power breaks the theoretical limit defined by the Debye length in macroscale channels. Plug flow generates 5 orders of magnitude more electricity than continuous flow (i.e., streaming current) and more than other technologies using falling water. Plug flow triggers a unique interfacial chemistry with large chemical potential of charge separation: the complete spatial separation of aqueous H^+^ and OH^–^ ions without the electric double layer. Having macroscale channels enables the energy of water from nature (e.g., rain or rivers) to be harvested freely. The simple setup lights up multiple LEDs continuously, modifies surfaces, and performs chemical reactions. Plug flow via harvesting energy from nature is a source of renewable power with many advantages for achieving sustainable societies.

## Graphical abstract

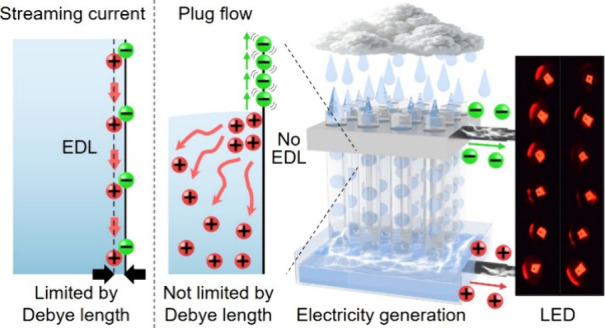

## Introduction

Novel sources of clean energy need to be developed to meet the increasing demand of energy globally, address the limitations of current sources of alternative energy (e.g., production of hydroelectric power is geographically limited and causes environmental damage), and ensure sustainability of societies. Charge separates spontaneously at the solid–liquid interface.^1–3^ Hence, electric charge can be generated by simply flowing water across surfaces for production of electricity. Previous studies have investigated this solid–liquid charge separation in various circumstances, including flows in channel, directed impact on a surface, splashing, and droplet flows.^3–7^ Because electricity can be generated directly by flowing water (e.g., from natural sources) across a surface, this phenomenon may potentially give rise to a method for harvesting green and renewable energy. However, only negligible amounts of charge can be produced by flowing a liquid across a surface as discussed in previous studies. Hence, this natural phenomenon of charge separation at the solid–liquid interface has not previously been considered a viable source of electricity generation.

The fundamental reason that only negligible amounts of electricity can be generated is because solid–liquid charge separation is a surface phenomenon; hence, charge is highly limited to only the surface. When charge separates at the solid–liquid interface, the electric double layer forms with charges on the surface and a layer of oppositely charged free ions attracted to the surface (part (i) of Figure 1a). The characteristic distance of this layer of free ions away from the charged surface is the Debye length, κ^–1^, as described in eq 1.^1^

1 $$\kappa^{-1}={\left(\frac{\underset{i}{\sum }c_{i}{z_{i}^{2}e}^{2}}{\varepsilon_{0}\varepsilon k_{B}T}\right)}^{-1/2}$$

where *c*_*i*_ (number/m^3^) is the concentration of the ionic species *i*, *z*_*i*_ is the charge number of ionic species *i*, *e* is the elementary charge, ε_0_ is the permittivity of free space, ε is the dielectric constant, *k*_B_ is the Boltzmann constant, and *T* is temperature. Using this formula, the Debye length for pure water at pH 7 is about 1 μm. The Debye length for water in equilibrium with atmospheric air at 298 K with dissolved carbon dioxide (i.e., pH around 5.6) is about 220 nm.^8^ If ions (e.g., salt) are dissolved in water, the Debye length is further reduced. Hence, the Debye length is very small at the nanometer scale.

**Figure 1:**
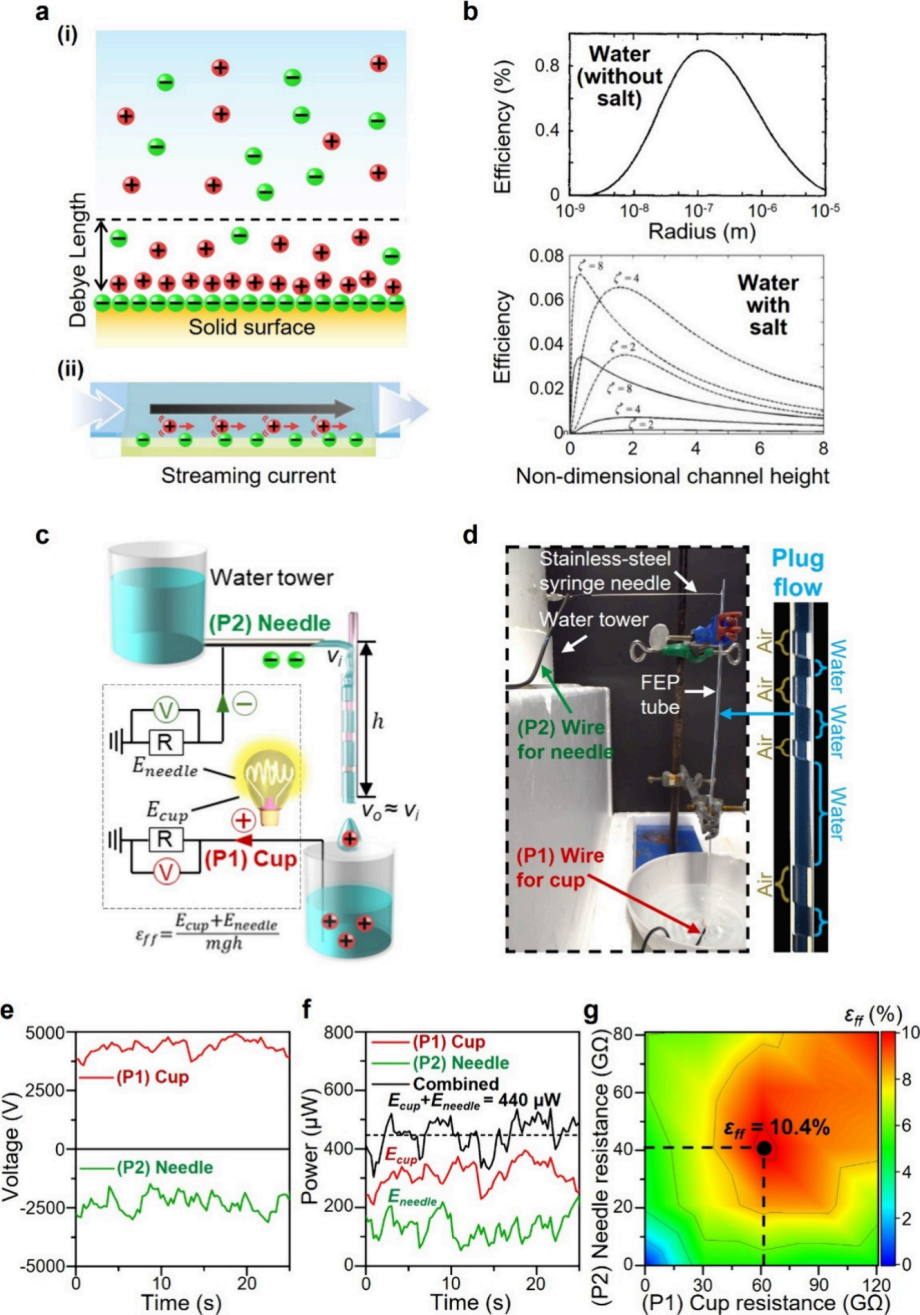
Plug flow in a tube generates electricity effectively. (a) Illustrations of the (i) electric double layer and (ii) mechanism of streaming current. (b) Theoretical plots of the energy efficiency by streaming current systems using pure water (top) and water with salt ions across surfaces with different charge densities (bottom). Nondimensional channel height, *K* = *h*/κ^–1^, is the ratio between the height of the channel, *h*, and the Debye length of the aqueous solution, κ^–1^. Top plot reproduced with permission from ref 11. Copyright 1965 American Institute of Physics. Bottom plot reproduced with permission from ref 19. Copyright 2008 Wiley-VCH. (c) Simple experimental setup for generating plug flow. Power is harvested from both points (P1) and (P2) simultaneously. (d) Images of the setup. The overall view shows the water tower on the left and water flowing out of the tower via a horizontally oriented needle into a vertically oriented FEP tube. The plug flow of water in the tube (image on the right). (e) Potential and (f) power generated continuously by the plug flow down a single small tube of only 2 mm in diameter. (g) Efficiency of power generation with varying resistive loads used at points (P1) (*x*-axis) and (P2) (*y*-axis).

To generate electricity, the most widely studied approach in previous research is streaming current. This system involves driving a continuous flow of aqueous solution through a small channel. The flow drives the layer of free ions close to the charged surface (i.e., a distance determined by the Debye length) to the outlet of the channel for the production of streaming current and electricity (part (ii) of Figure 1a).^7,9,10^ The efficiency of the system is usually defined as the amount of electricity generated divided by the amount of energy needed to drive the system (e.g., by the pump). When water (in equilibrium with air) is used, the maximum efficiency is almost negligible at less than 1%, regardless of the size of the channel (plot on top in Figure 1b).^11^ When the size of the channel is around the Debye length of 220 nm, the efficiency decreases rapidly with increasing size. The efficiency becomes practically completely negligible beyond 10 μm.

To increase efficiency, previous studies have considered many improvements, including using small nanoscale channels, adding salt to water (plot at the bottom in Figure 1b), increasing surface charge density, or modifying the system to prevent charge recombination.^12–17^ Despite these improvements, the trend remains the same in all previous theoretical or experimental studies on streaming current: the efficiency of the systems decreases rapidly when the channel size increases beyond the Debye length.^11,18,19^ No previous study has reported any significant efficiency when the channel size is beyond 10 μm. Because charge separation is a surface phenomenon, these results are fundamentally expected: the amount of interfacial area for charge separation becomes negligible in larger (e.g., >10 μm) systems.

It is critically needed to use macroscale (e.g., millimeter scale) channels for harvesting renewable energy of water from natural sources (e.g., rain or rivers). Water from natural sources cannot flow through small nanoscale channels naturally. For studying the production of streaming current, pumps are always used to drive liquid through the small channels. However, the very small efficiencies reported in all the previous studies on streaming current (i.e., a few to less than one percent) are based on the far greater energy needed by the pumps than the electricity generated;^11^ thus, it is currently impractical to use streaming current for generating electricity. At least millimeter-sized channels are needed to allow water from natural sources to flow through them naturally, without the need for a pump. Therefore, it is crucial to find methods to break the fundamental limit defined by the Debye length to effectively generate electricity using macroscale channels and using water from natural sources.

Previous research works have thus relied on other fundamental mechanisms instead. One method involves electrostatic induction. Technologies that use electrostatic induction include the Kelvin water dropper and droplet-based electricity generators.^20–33^ These systems typically involve first charging a solid surface and then flowing water droplets through or over the charged surface. Some studies reported first precharging the surface by either ion injection (i.e., a step that requires energy input) or charging very gradually via solid–liquid charge separation (i.e., a process that requires many droplets and a long time of charging).^22–24,28^ After charging the surface, water droplets are flowed across the charged surface. An electrode underneath the surface senses the changing amounts of charge via repeatedly having droplets on the charged surface or not by electrostatic induction for generating electricity. These devices are only able to produce transient pulsed (i.e., instantaneous but not continuous) power. Although the peak instantaneous powers of these devices are high, the duration of each instantaneous power was very short and ranged typically from 0.1 to 1 ms. To understand the effectiveness of these devices as a power source, it is needed to determine the average continuous power density instead of the reported peak instantaneous power. The average continuous power density can be determined by taking into account the energy harvested from one droplet of water, the frequency of droplets contacting the surface, and the surface area of the device. The highest average continuous power density of these devices was only on the order of 0.1 to 1 W/m^2^ (i.e., excluding devices combined with other energy-generating systems such as solar panels).^22,24,25,28^ Importantly, electricity is generated by electrostatic induction of the pre-existing and steady amount of charge present on the surface—the charge on the surface is not directly harvested as electricity. Because streaming current harvests the constantly separated charge directly as electricity, the mechanisms of these droplet-based generators are fundamentally different from streaming current.

This manuscript reports the surprising natural phenomenon that a high efficiency of electricity generation of >10% can be achieved by flowing water across a macroscale (i.e., millimeter-sized) channel. Because the system involves only flowing water through a channel, the electricity is generated by the separation of charge at the solid–liquid interface (i.e., not by other fundamental mechanisms). We found that the key to generating high efficiency of electricity generation is by using an optimal flow pattern: a plug flow (i.e., short columns of water separated by air). The high power efficiency of >10% using a millimeter-sized channel indicates that the fundamental limit set by the Debye length is broken by the plug flow. Importantly, because a macroscale channel is involved, water from natural sources (e.g., rain and rivers) can flow through the channel under its own weight (i.e., without the need for a pump). Therefore, renewable energy from nature can be harvested freely for generating electricity.

## Results

### Plug Flow Enables Highly Effective Power Generation

We showed that a typical experimental setup involved only simple materials: a makeshift tower for storing water (i.e., polymeric containers or plastic bottles; 0.75 – 1.65 m in height), a metallic needle attached to the bottom outlet of the tower, a polymeric tube, a cup for collecting water, and deionized water (Figures 1c and 1d; Figure S1; Methods). We used fluorinated ethylene propylene (FEP) tubes due to their superior charging property, without any treatment (e.g., no initial charging needed; Section S1 and Table S1). Importantly, its macroscale diameter of 2 mm (length: 32 cm) allowed water to fall naturally downward by gravity. Operationally, we only needed to fill the tower with water. The water then flowed out of the tower via the horizontally oriented metallic needle and collided with the top section of the vertically oriented tube that was cut in half. This head-on collision between the water and the surface of the tube caused the subsequent downward wavy flow that mixed air naturally into the column of water, thus creating a plug flow subsequently in the tube. The plug flow consisted of discrete columns of water separated by pockets of air in the tube (Figure 1d; Movie 1). The water was collected in the cup placed below the tube. We harvested power from two points: (P1) the water collected in the cup and (P2) the top of the tube.

Electricity was generated whenever water flowed through the tube. We obtained a positive current from (P1) and a negative current from (P2) simultaneously. After optimizing the many parameters of the setup (i.e., including the resistive loads at (P1) and (P2)), we measured a surprisingly high average power of 440 ± 13 μW from both the points (P1) and (P2) when water flowed (80 mL/min) through only one tube with the small diameter of 2 mm (Figures 1e–1g). This amount corresponds to a large average power density on the order of ∼100 W/m^2^ (i.e., based on the tubes with diameters of 2 mm arranged vertically in a close-packed structure and only a single-stage setup of 32 cm high). We define efficiency, ε_*ff*_, as the percentage of the electric energy generated over the loss in potential energy of the water as it flows down the tube. We obtained an optimal efficiency of 10.4%; hence, electricity was generated directly with a high efficiency by simply harvesting the potential energy due to the elevation of water (Section S2).

Kinetic energy was approximately constant throughout the system and relatively negligible; hence, it was not included in the calculation of efficiency (Section S3). In fact, we measured that the velocity of the flow was slightly higher at the outlet (i.e., *v*_o_ = 0.4 m/s) than at the inlet (i.e., *v*_i_ = 0.35 m/s) of the tube. Because the system produced more kinetic energy than it received, kinetic energy was not responsible for the generation of electricity. Furthermore, the kinetic energy was only around 2% of the loss of potential energy of the water. The velocity at the inlet of the tube was far lower than the typical speed of rain drops (e.g., the terminal velocity of rain is more than an order of magnitude than the inlet velocity at ∼9 m/s). Hence, it is possible to harvest energy directly from rain, without the need for the water tower.

The results showed that the power generated by the system fluctuated with time but remained continuous throughout time (i.e., instead of pulsed instantaneous power). Hence, the system is able to provide continuous power without interruptions. On the other hand, the plug flow was discontinuous and consisted of columns of air in between charged water. To study the continuous generation of power, we stopped the flow of water in the system and monitored the power generation. We found that electricity continued to flow for roughly ∼1 s even after the flow stopped (Figures 2a and 2b). This duration of continued supply of electricity without the flow of charged water was much longer than the duration taken by a single column of air to flow out of the tube at around ∼0.015 s. Therefore, electricity could be supplied continuously even when air was flowing out. The reason for the continued supply of electricity was possibly due to accumulated charge within the system that flowed out whenever air was flowing out instead of water (see Section S4 for a more detailed discussion).

**Figure 2:**
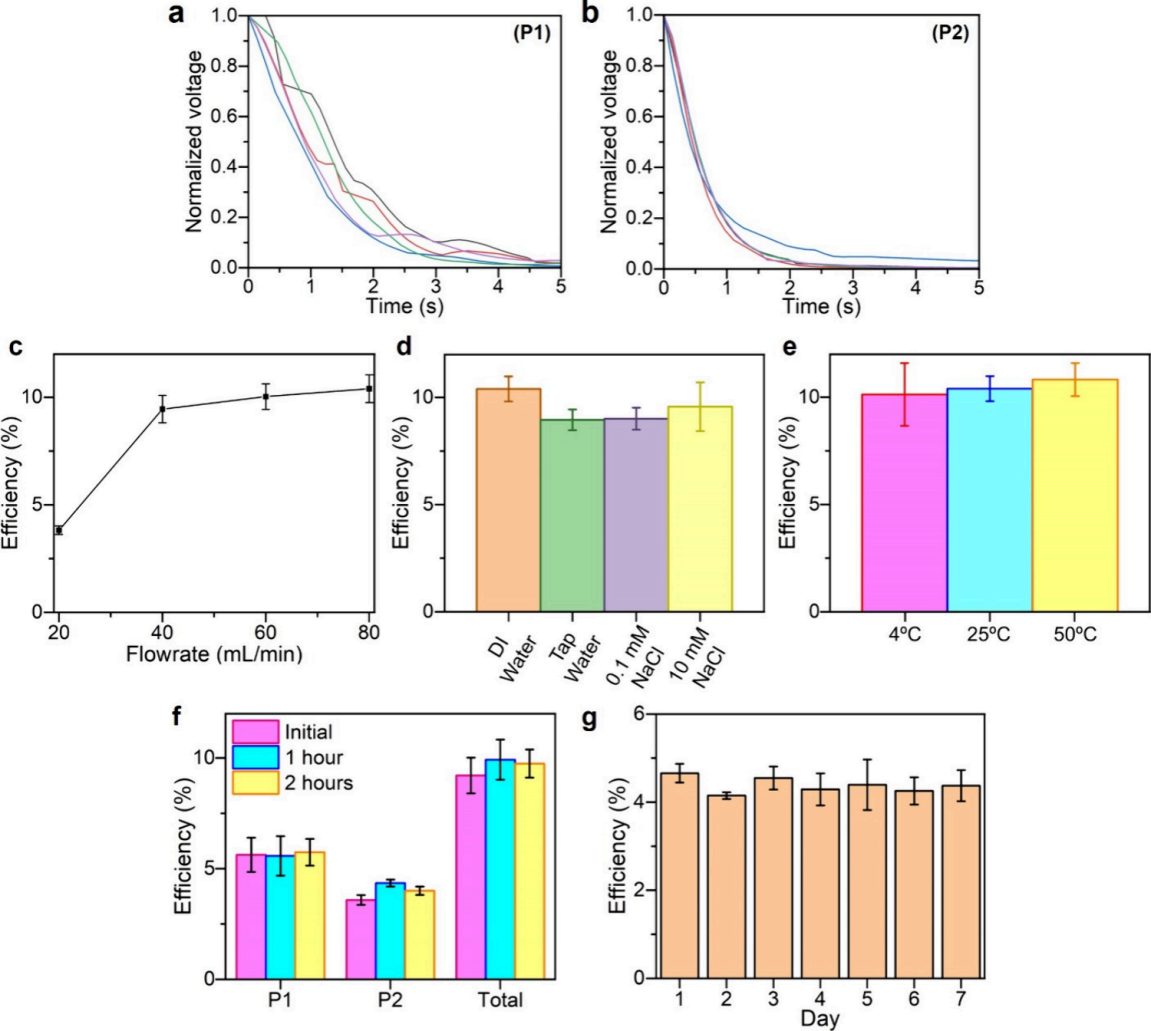
Plug flow provides consistent and highly efficient power generation under different environmental conditions. Continuous power generated as determined by decays of voltage after stopping plug flow measured at (a) (P1) and (b) (P2). Efficiencies of power generation were similar when varying (c) flow rate (40–80 mL/min), (d) quality of water, and (e) temperature of water. Long-term consistent power generation as shown by (f) uninterrupted plug flow using a water tower for 1 min (“initial”), 1 h, or 2 h and (g) plug flows across 7 days. Power was harvested using the optimal setup from (P1) with a resistive load of 61 GΩ and from (P2) with a resistive load of 41 GΩ in parts (a)–(f). For part (g), power was harvested only from (P1) with a resistive load of 31 GΩ.

We verified that the electricity generated was due to the separation of charge at the solid–liquid interface by directly measuring the amount of charge in the water after flowing out of the FEP tube by a Faraday cup connected to an electrometer. The amount of charge measured by the Faraday cup matched the electricity generated by the system (Section S5).

The effect was additive for scaling up the system in both the lateral plane and vertical direction. We obtained twice the power by flowing water simultaneously through two tubes. Twice the power was also obtained by first flowing the plug flow of water through one tube, harvesting the power, and then flowing the same volume of water through a second tube that was placed vertically below the first tube and harvesting the power again.

We investigated the influences of different parameters of the system on the efficiency of power generation (Section S6). We first varied systematically the length of the tube and found that the voltage and current from both (P1) and (P2) increased linearly with increasing length of the tube until around 32 cm, beyond which they did not increase further. We varied the flow rate and found that the efficiency remained at more than 90% of the optimal efficiency of the system for a large range of flow rates from 40–80 mL/min (Figure 2c). The efficiencies generated by tap water, 0.1 mM NaCl solution, and 10 mM NaCl solution were more than 85% of the optimal efficiency of the system via using deionized water (Figure 2d). The efficiencies generated by using water with different temperatures of 4, 25, and 50 °C were found to be statistically similar (Figure 2e). Therefore, it seemed that the efficiency of power generation by the system remained similar under different environmental conditions.

We found that the system produced consistent power after repeated and long-term use (Section S7). Similar efficiencies were obtained after flowing plug flows of water through the FEP tube at (P1) and (P2) for a duration of either 1 min, 1 h, or 2 h (Figure 2f); hence, the overall efficiencies of the system were similar for all three durations. In another set of experiments, we flowed plug flows of water through the FEP tube five times each day, 1 min each time, for 7 days in a row (Figure 2g). Similar efficiencies were obtained throughout the 7 days.

### Relationship between Flow Patterns and Generation of Electricity

We investigated the relationship between flow pattern and generation of electricity from the solid–liquid interface. For a fair comparison, we obtained different patterns of fluid flow by making only minor changes to the setup while keeping the important aspects (e.g., flow rate) the same. By changing only the diameter of the tube and orientation of the metallic needle (i.e., horizontally or vertically), we obtained five types of flow patterns (Figure 3a and Section S8): plug flow (2 mm tube), plug-dripping flow (3 mm tube; Movie S2), dripping flow (6 mm tube; Movie S3), rivulet (6 mm tube; Movie S4), and full flow in a tube (1 mm tube; Movie S5). Our results showed that plug flow generated 5 orders of magnitude more electricity than the full flow (Figure 3b and Table S2); hence, the flow pattern was critical for power generation. Different flow patterns generated vastly different amounts of power. These results showed that the type of flow pattern effectively controlled the amount of charge generated in the liquid.

**Figure 3:**
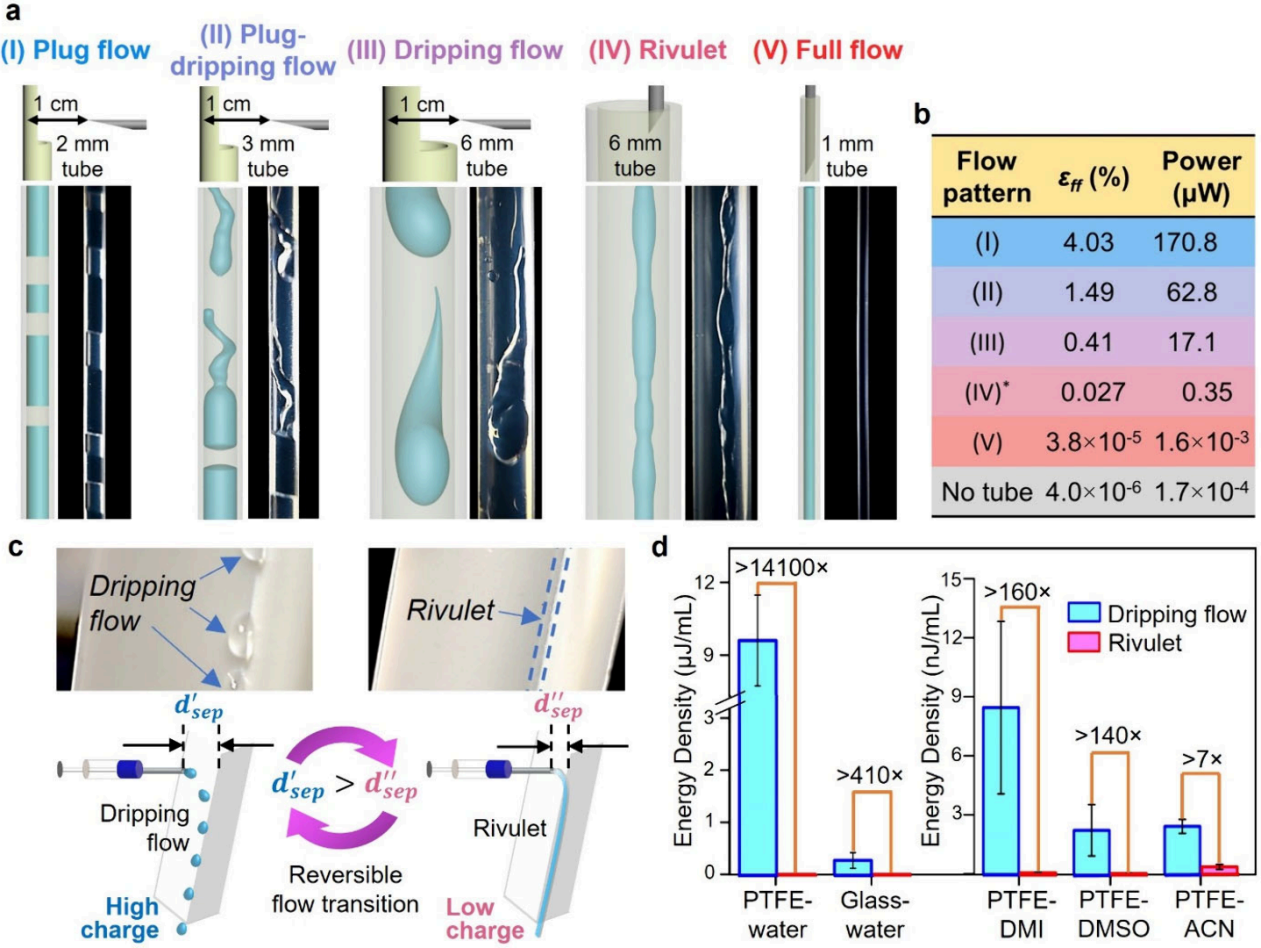
Flow pattern critically affects generation of electricity from solid–liquid interface. (a) Five different flow patterns (I–V) investigated. (b) Efficiency, ε_*ff*_, and power generated by the five flow patterns shown in (a) by harvesting from only (P1) with a resistive load of 31 GΩ. * indicates a shorter tube used (see Table S2). Grounding (P2) or not did not affect the result (Figure S2). (c) Reversible flow transition between dripping flow and rivulet by varying *d*_sep_. (d) Energy densities of dripping flow and rivulet using different solids (i.e., polytetrafluoroethylene (PTFE) and glass) and liquids (i.e., 1,3-dimethyl-2-imidazolidinone (DMI), dimethyl sulfoxide (DMSO), and acetonitrile (ACN)). The error bars represent the standard deviation.

We devised a reversible flow transition to show clearly the dramatically large effect of the flow pattern on charge generation in real time. The experiment involved flowing water out of a syringe needle and onto an inclined V-shaped surface (polytetrafluoroethylene, PTFE; Figure 3c). The transition was induced via changing only the distance of separation, *d*_sep_, between the syringe needle and the polymeric surface (Methods, Section S9, and Figure S3). The flow was a rivulet when *d*_sep_ was small (i.e., ∼0.5 mm) and was dripping when *d*_sep_ was large (i.e., >2 mm). Results showed that the energy density produced by the dripping flow was 14,100 times greater than that produced by the rivulet (Figure 3d). The dramatically different amounts of power generated can be reproduced easily by reversibly and repeatedly inducing the dripping-rivulet flow transition dynamically in real time ( Movie S6). Besides *d*_sep_, all other aspects of the experimental setup remained the same; thus, the flow pattern was the cause of the difference in charge generation. We showed that this phenomenon was general for different liquids—including organic liquids—and solids (e.g., glass).

### Discontinuity in Flow Triggers Unique Interfacial Chemistry

The flow patterns (i.e., the plug and dripping flow) that generated larger amounts of electricity had discontinuities within the flow, whereas the flow patterns (i.e., rivulet and full flow) that generated lower amounts of electricity were continuous. We investigated the fundamental role of discontinuity by first studying the polarity of charge generated by a continuous flow (Figure 4a) and discontinuous dripping flow (Figure 4b). We first coated surfaces of glass with different types of polycations (poly­(ethylenimine) (PEI), poly­(allylamine hydrochloride) (PAH), poly­(diallyldimethylammonium chloride) (PDDA)) or polyanions (poly­(sodium 4-styrenesulfonate) (PSS)) (Figure 4c; Methods; Section S10; Tables S3–S5). We verified that the surfaces were uniformly coated with the polyelectrolytes via labeling the coated surfaces with a fluorescent dye and observing the surfaces by confocal microscopy, X-ray photoelectron spectroscopy (XPS), and contact angle measurement.

**Figure 4:**
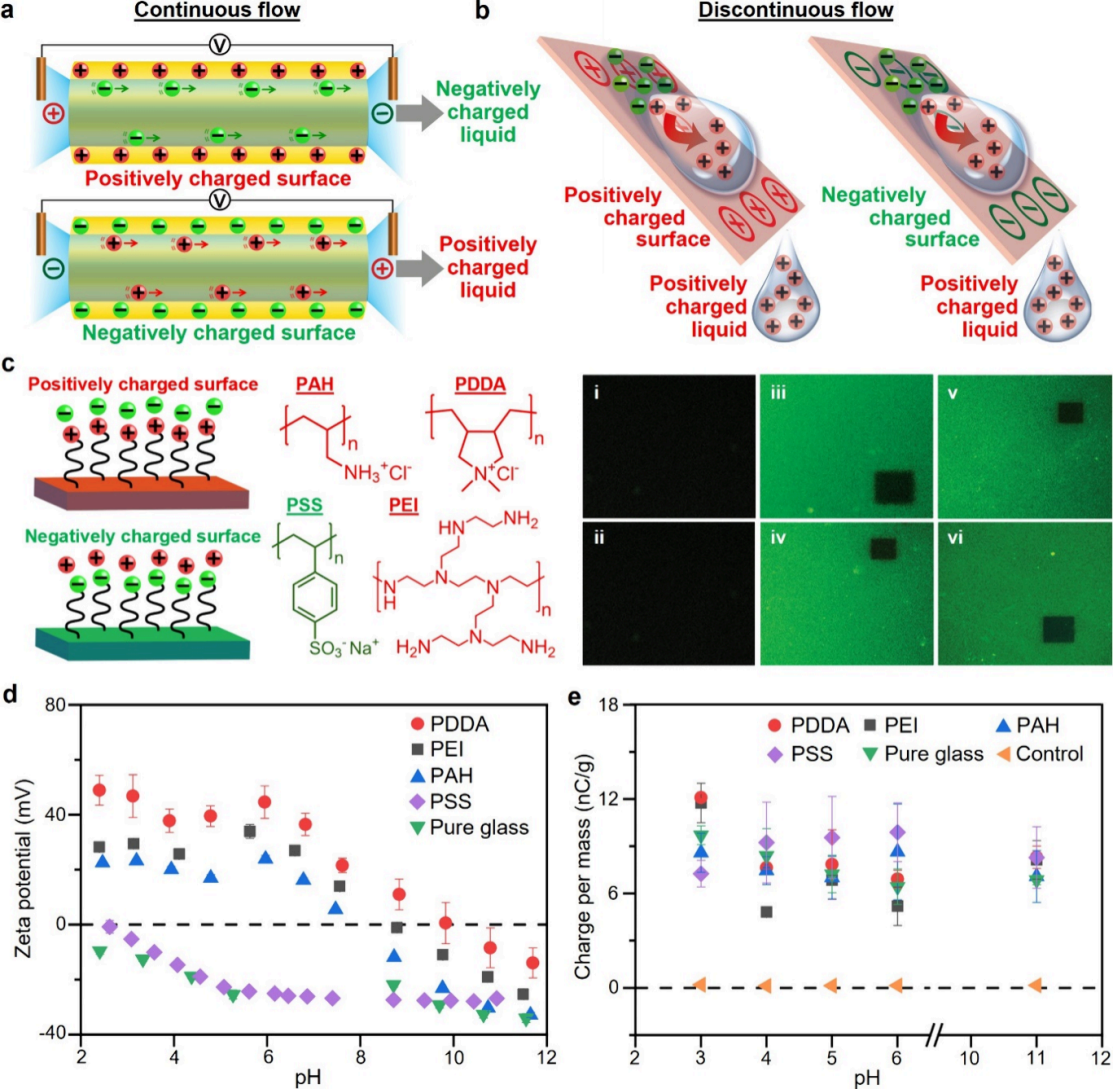
Flow with discontinuity produces polarity of charge that is different from continuous flow. (a) Streaming current by continuous flow charges liquid either positively or negatively. (b) Unique charging mechanism by discontinuous flow charges liquid only positively. (c) Scheme shows surfaces coated with polyelectrolytes. The polycation used was either poly­(ethylenimine) (PEI), poly­(allylamine hydrochloride) (PAH), or poly­(diallyldimethylammonium chloride) (PDDA). The polyanion used was poly­(sodium 4-styrenesulfonate) (PSS). Experimental images on the right show glass surfaces (i, ii) not coated, (iii, iv) coated with PEI, or (v, vi) coated with PAH labeled with a fluorescent dye. Surfaces (i, iii, v) before and (ii, iv, vi) after flowing water across them for 5 min. In each of the images shown in parts iii–vi, the sharp contrast in color between the manually bleached dark squarish region (i.e., via exposure to a high-intensity light) and the rest of the image showed that the surface was coated with the polyelectrolytes labeled with the fluorescent dye. (d) Zeta potential of the solid surfaces analyzed by flowing continuously solutions of different pH across uncoated or coated surfaces. (e) Charge per unit mass of solutions of different pH by having discontinuous flows across the same surfaces as (d). All error bars in this figure represent the standard deviation.

We then flowed continuous streams of aqueous solutions of different pH values across surfaces uncoated or coated with the different types of polyelectrolytes in an electrokinetic analyzer and measured the zeta potentials of the surfaces (Figure 4d). The polarity of the liquid at the outlet is opposite to that of the zeta potential of the surface due to the flow of the mobile counterions in the liquid to the outlet. Our measurements indicated that the different liquids of the continuous flows charged positively or negatively depending on the pH and type of surface as expected. In particular, the liquid was *negatively* charged in many cases, including those that involved the many types of positively charged surfaces (i.e., PEI, PDDA, and PAH; Figure 4c) and wide ranges of pH (i.e., ∼pH 2 to pH 8). Subsequently, we flowed the discontinuous drips of aqueous solutions with different pH (i.e., ∼pH 3 to pH 11) across the same uncoated or coated surfaces (Figure 4e). We performed a control experiment in which the discontinuous drips did not come into contact with any surface (“Control” in Figure 4e). Results showed that the discontinuous flow charged the liquid unexpectedly only *positively* for all the cases and more positively than the “Control”. This result showed that the mechanism of the discontinuous flow was fundamentally different from that of the continuous flow.

Because water charged only positively regardless of the type of surface, the charged species generated in the water is probably not related to the surface (e.g., the mobile counterions of the polyelectrolytes), but the water itself (Figure 5a). Previous studies have reported that aqueous OH^–^ ions preferentially adsorb over aqueous H^+^ ions onto solid surfaces.^34–36^ To investigate the role of OH^–^ ions, we measured the pH of basic (pH 9) aqueous solutions before and after flowing either the discontinuous drips or a continuous stream down an inclined V-shaped PTFE surface (Section S11). Results showed that pH decreased significantly (i.e., ΔpH ≈ 0.3) when the flow was discontinuous, but was negligible when the flow was continuous (Figure 5b). No change in chemical composition of the water was detected by NMR (Figure 5c). Similarly, no change in chemical composition of the surface of the FEP tube before and after flowing water was detected by Fourier-transform infrared spectroscopy (FTIR; Figure 5d) and X-ray photoelectron spectroscopy (XPS; Figure 5e), possibly because the changes were small. For higher sensitivity,^37^ we used time-of-flight secondary ion mass spectrometry (ToF-SIMS) to detect the adsorption of OH^–^ ions on the inner surface of the FEP tube before and immediately after flowing plug flows of water or fully continuous flow. The result of analyzing the surface after contacting the plug flow showed a strong peak that represented OH^–^ intensity at *m*/*z* = 17 appeared (“Plug flow” in plot II, Figure 5f). On the other hand, the OH^–^ intensities of the surfaces before contacting any flowing water (“Dry tube” in plot I, Figure 5f) and after contacting the full continuous flow (“Full flow” in plot III, Figure 5f) were similar and negligible. These results indicated that OH^–^ ions absorbed onto the inner surface of the FEP tube after flowing plug flow across the tube.

**Figure 5:**
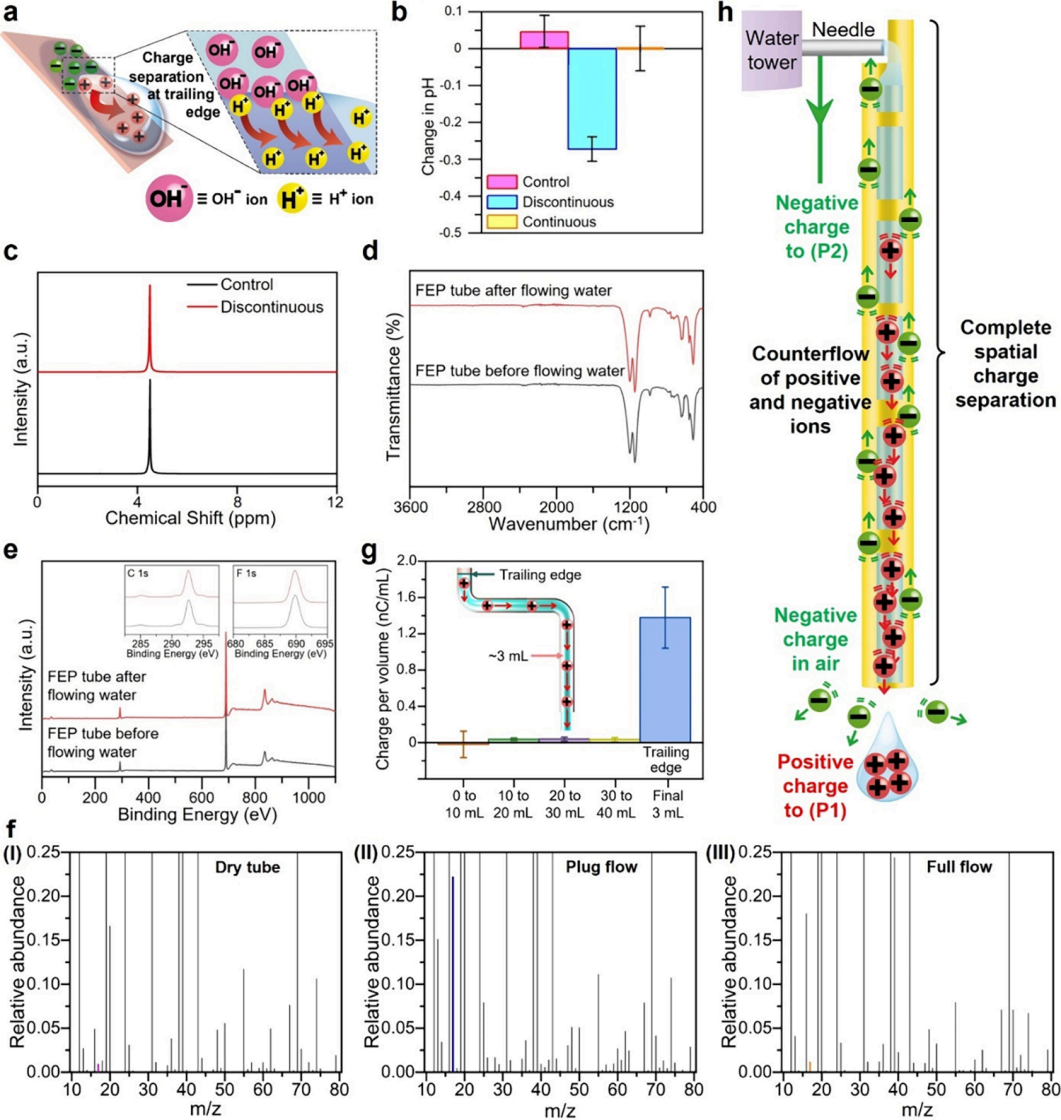
Discontinuity triggers unique molecular charge separation and charge transport. (a) The unique molecular mechanism of localized charge separation by discontinuous flows. (b) Change in pH of solution when it was not flowed across any surface (“Control”), flowed as discontinuous drips across a surface, or flowed as a continuous stream across a surface. (c) NMR spectra of the discontinuous drips of water down a FEP tube and discharged water without flowing through any tube (“Control”). (d) FTIR and (e) XPS survey spectra of the FEP tubes before (black line) and after (red line) flowing water through them. The insets show the high-resolution spectra of C 1s and F 1s. (f) Normalized time-of-flight secondary ion mass spectrometry (ToF-SIMS) spectra of the surfaces of the FEP tubes with the OH^–^ peak shown at *m*/*z* = 17 for (I) before flow (“Dry tube”; red bar), (II) after plug flow (“Plug flow”; blue bar), or (III) after full continuous flow (“Full flow”; brown bar). (g) Charges of different portions of a long discrete column of water that flowed through the tube. (h) Scheme illustrates the large-scale charge transport by the plug flow that involves the counterflow of the positive and negative charges throughout the tube. All error bars in this figure represent the standard deviation.

We investigated the location of charging at the solid–liquid interface by the plug flow. In this experiment, we flowed a single long column of water with a total volume of 43 mL through a relatively short FEP tube (i.e., the overall system had a volume of only 3 mL) (Section S12) only once. Measurements showed that the charge generated in the first 40 mL of the fully continuous flow through the tube was negligible (Figure 5g and Figure S4). As soon as the trailing edge appeared at the beginning part of the tube (i.e., the last 3 mL left in the system), however, the charge measured increased dramatically. Results showed that charge was located in the last 3 mL of the water that remained in the tube after the trailing edge appeared. The charge generated right at the trailing edge possibly dispersed rapidly into the bulk 3 mL of water due to the repulsive electrostatic forces among the charged species. Hence, this result indicated that charging did not occur at the leading edge or the continuous section of the plug flow, but at the trailing edge. We showed that these results were general for tubes of different sizes (Figure S5).

For this experiment that involved flowing a single column of water through the tube only once, we obtained a high 1.6 nC/mm of charge per unit length of the trailing edge. For the typical plug flow that involved passing through many discrete columns of water (i.e., Figure 3a), the charge per unit length of the trailing edge was 0.8 nC/mm.

We investigated the possibility of migration of ions on the inner surface of the FEP tube. Due to the constant flow of water through the tube, the surface of the tube is expected to be highly moisturized. We measured experimentally that the surface resistivity of a moist FEP surface was 10^9^ Ω/sq (Section S13), which corresponds to an overall surface resistance of the inner surface of the tube of 10^11^ Ω. Because the order of magnitude of this resistance is similar to the resistive loads used in the optimal system, it is possible for ions to migrate across the surface of the FEP tube.

Based on all these results, we propose a mechanism by which the plug flow through a tube separates charge effectively at the solid–liquid interface (Figure 5h). A mechanism that is not specific to the type of surface is the separation of H^+^ and OH^–^ ions of water. Water molecules undergo self-ionization to form H^+^ and OH^–^ ions. Previous studies have reported that OH^–^ ions of water have the tendency to preferentially adsorb over H^+^ ions at the solid–liquid interface. The phenomenon is general and occurs for both hydrophilic and hydrophobic surfaces (e.g., polymeric surfaces, vesicles, and self-assembled monolayers on substrates).^36,38–43^ Results from molecular dynamics simulations also showed that OH^–^ ions tend to adsorb preferentially (i.e., more than the hydronium ions) at the interface of water and solid.^34–36,44^ H^+^ ions, on the other hand, are known to have superior mobility in aqueous media compared to other types of ions.^45–50^

When the discrete column of water moves down the tube, its trailing edge retracts from the surface of the solid. The nonequilibrium dynamics at the contact line between water and the solid surface at the trailing edge pulls the highly mobile H^+^ ions away from the solid–liquid interface and into the bulk volume of water (i.e., and causes the pH to decrease), thus charging the liquid positively. The less mobile aqueous OH^–^ ions preferentially adsorb and remain on the surface, thus charging the surface negatively. The self-ionization of water generates around 10^–7^ M of OH^–^ ions. Based on a typical flow rate of 80 mL/min, we determined that the quantity of negative charge generated by the system per unit time is around more than 2 orders of magnitude less than the quantity of OH^–^ ions produced by the self-ionization flowing through the tube per unit time. Therefore, it is plausible that the negative charges generated by the system are due to the OH^–^ ions produced by the self-ionization of water. This mechanism allows water to charge positively regardless of whether the surface is composed of nonionic, ionizable, or ionic functional groups.

Importantly, the localized charge separation at the receding line needs to be coupled with large-scale charge separation. If the negative charge kept accumulating on the surface, saturation of the negative charge would reach; thus, subsequent columns of the plug flow would not charge anymore. We determined experimentally, however, that the negative charge did not accumulate much on the solid surface even as the water gained far greater amounts of positive charge (Section S14.1 and Figure S6). In fact, we measured a substantial amount of negative charge that migrated upstream to (P2). The dynamics of the plug flow in the tube thus produces the counterflow transport of ions for large-scale charge separation: the positive ions flow down the tube together with the liquid and negative ions migrate up the tube (e.g., along the surface) throughout the length of the tube (Figure 5h). The large chemical potential for localized charge separation at the receding line enables the positive and negative ions to remain separated even as they migrate in the counterflow manner. The full coverage of the columns of the plug flows across the entire circular circumference of the tube provides moisture for increasing surface conductivity, thus possibly allowing the negative ions to migrate rapidly upward the moist surface.

We found that negative charge dissipated into the surrounding air only when the flow was discontinuous (Section S14.2 and Figure S7). This dissipation thus accounted for the slightly smaller negative current at (P2) than the positive current at (P1) (Figure 1e).

The other flow patterns investigated have less localized and large-scale charge separation. The plug flow generated greater power than the plug-dripping and dripping flows probably because it has a larger solid–liquid contact for greater localized and large-scale charge separation. The amounts of power generated by the rivulet and full flow are much smaller possibly due to the lack of discontinuities (Section S15); thus, the receding line is not present for the unique localized charge separation with large chemical potential. The rivulet generated greater power than the full flow possibly due to the random fluctuations along the sides of the rivulet. Hence, irregularities of the flow pattern generate power more effectively.

### Applications of Electricity Generated by Plug Flow

We showed that the plug flow through four tubes connected electrically to an external circuit that increased the current while reducing the potential correspondingly generated electricity effectively for powering LEDs (Figure 6a; Section S16.1). Our results showed that 20 s of plug flow through the tubes lit up 12 LEDs brightly and constantly for >20 s; hence, the power supplied was continuous (Figure 6b; ; Movie S7).

**Figure 6:**
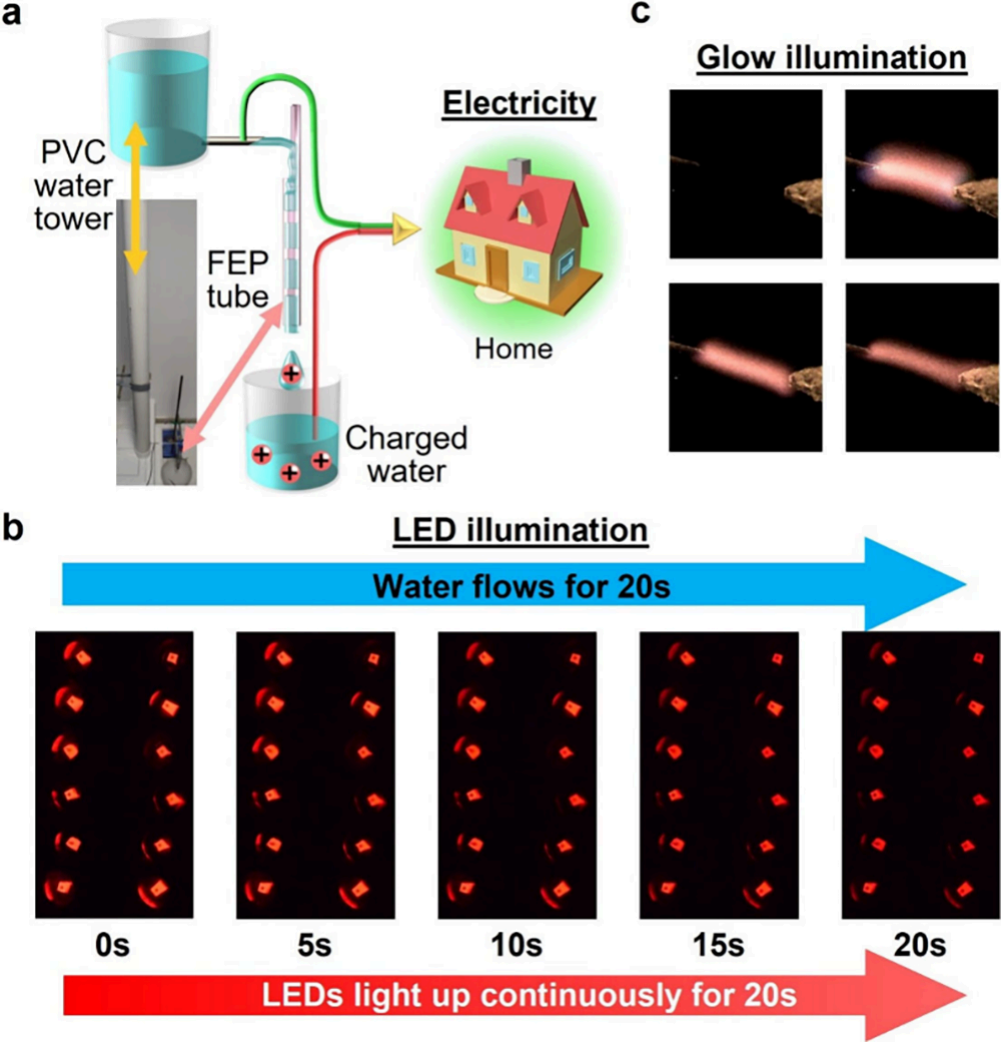
Plug flow of water in tubes generates renewable electricity for illumination. (a) Simple setup for generating electricity (i.e., without using any equipment). Plug flow produced electricity for (b) lighting up 12 LEDs continuously and (c) generating bright glow between two metallic needles.

In addition, we showed that the plug flow could be used to generate illumination based on the same fundamental mechanism as modern lamps (e.g., fluorescent lamps). We first aligned two metallic needles horizontally with a gap of ∼1 mm under a helium atmosphere (Figure 6c and Section S16.2). One needle was connected electrically to (P1), whereas the other was connected to (P2). When we flowed plug flow through two tubes, a bright glow between the needles was produced ( Movie S8).

We further showed that the electricity generated by the plug flow could be used directly in a variety of important applications (Figure 7a and Sections S16.3–S16.7). We first showed the application of performing chemical reactions. We brought the needle connected to (P1) close to an aqueous droplet that contained methylene blue. With the plug flow, the droplet changed from blue to colorless (Figure 7b). Analysis by the ultraviolet–visible spectroscopy showed that the methylene blue reacted completely. We then showed that the electricity produced a large amount of radicals for applications. We repeated the experiment by using a droplet that contained the radical scavenger 2,2-diphenyl-1-picrylhydrazyl (DPPH) dissolved in *N,N*-dimethylformamide (DMF). DPPH is a standard chemical used to detect the presence of radicals. With the plug flow, the droplet changed from its original purplish red to pale yellow (Figure 7c). Analysis by ultraviolet–visible spectroscopy showed that DPPH reacted completely, thus verifying that a large amount of radicals was produced.

**Figure 7:**
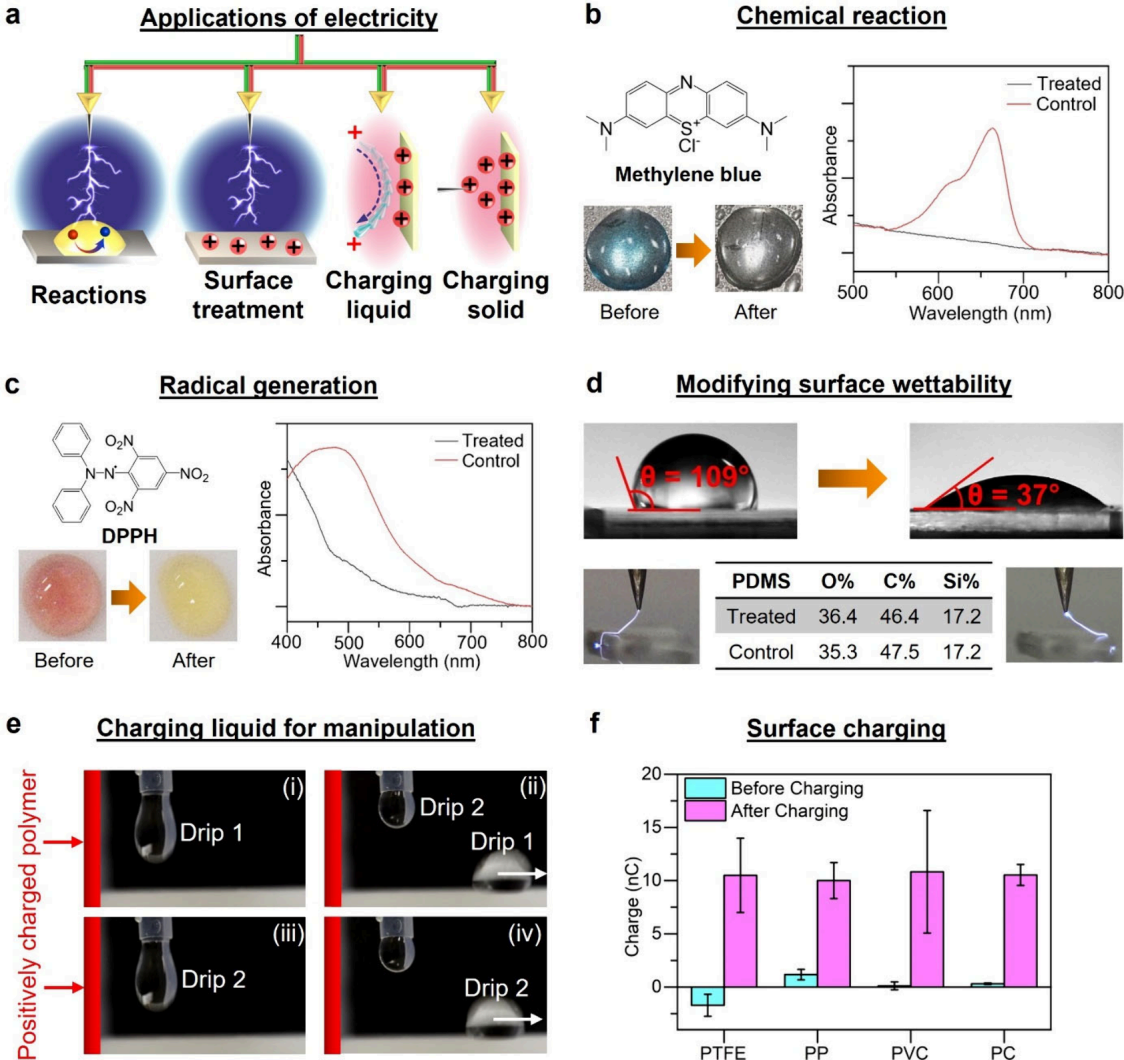
Practical applications of the renewable electricity produced by the plug flow of water in tubes. (a) Scheme shows the general applications. Plug flow produced electricity for (b) performing chemical reactions, (c) generating radicals, (d) modifying wettability of surfaces, (e) manipulating liquid, and (f) charging surfaces. The error bars represent the standard deviation.

We used the electricity for surface treatment. We brought the needle connected to (P1) close to the surface of polydimethylsiloxane (PDMS). With the plug flow, electric sparks were frequently produced and observed by the naked eye (Figure 7d; Movie S9). The contact angle of the PDMS changed greatly from 109° (i.e., hydrophobic) to 37° (i.e., effectively hydrophilic) after treatment due to the chemical functionalization of hydroxyl groups on the surface (Figure S9). This amount of change in wettability is comparable to standard methods (e.g., plasma or UV) that require the use of expensive equipment.

In addition, we found that the same method could effectively charge liquids and solid surfaces (Sections S16.6 and S16.7). Charged water and surfaces are important in many applications, including electrostatic separation, coating, and microfluidic manipulations.^22,51–55^ For charging water, we flowed drips of water through the tube. The water became highly positively charged and was found to repel rapidly away from a positively charged polymeric surface (i.e., charged by a simple electrostatic gun; Figure 7e; Movie S10). For charging surfaces, we brought the needle connected to (P1) close to surfaces of different materials. We found that the materials gained substantial amounts of electrostatic charge (Figure 7f) that were similar to those used in practical applications.^56^

## Discussion

Generating electricity based on harvesting charge separated constantly at the solid–liquid interface has not been considered as a viable source of power before. Previous studies that sought to use the phenomenon have obtained completely negligible amounts of electricity at the macroscale. For streaming current, its efficiency decreases rapidly with increasing channel size beyond the nanoscale Debye length and becomes completely negligible at the microscale. This surface phenomenon is fundamentally not practical for generating electricity due to the negligible interfacial area of macroscale channels.

We found that plug flow involves fundamentally a unique interfacial chemistry that has a huge chemical potential for charge separation. The huge chemical potential of separation gives rise to our result that plug flow in a macroscale (2 mm) tube generated electricity from the solid–liquid interface with a high efficiency of >10% and an average power density of ∼100 W/m^2^ per square meter of the possible horizontal catchment area of rain (i.e., based on the horizontal projected area for harvesting the energy of raindrops falling vertically downward). We found that plug flow generates electricity 5 orders of magnitude more than continuous flow (i.e., streaming current). This efficiency is many orders of magnitude higher than that possibly generated by streaming current using macroscale channels as reported theoretically and experimentally in previous studies. This result indicates that plug flow breaks the fundamental limit defined by the Debye length.

Importantly, the combination of high efficiency and use of macroscale tubes enables plug flow to harvest renewable and clean energy from nature freely, including directly from rain (Section S17) or other sources (e.g., rivers). Water can flow through millimeter-sized channels readily, but not smaller ones such as micro- or nanoscale channels which require pumping. The kinetic energy of falling raindrops is typically much larger than that needed to pass through the macroscale (i.e., 2 mm) tubes that we used in our experiments. Specifically, the speed of water through our tubes is around 0.4 m/s versus the speed of falling raindrops that is typically an order of magnitude larger.

It is unexpected that water flowing simply through a macroscale tube can generate electricity for practical applications. We showed that plug flow supplies substantial electricity for many important practical applications, including lighting up multiple LEDs continuously, modifying surfaces, and performing chemical reactions. Therefore, this is the first time that harvesting the constantly separated charge at the solid–liquid interface is shown to be a practically useful source of electricity.

Besides technologies that harvest charge separated constantly at the interface, the average power density of ∼100 W/m^2^ of plug flow is 2 to 3 orders of magnitude higher than that of the state-of-the-art technologies reported based on electrostatic induction of existing stable charge (i.e., not harvesting the charge directly as electricity).^21–25^ Plug flow provides continuous power instead of instantaneous power.

In addition, plug flow has many advantageous features. The setup is simple; no equipment is needed. Hence, it is inexpensive and environmentally friendly to install, operate, and maintain. The effect is additive; thus, scaling up can be achieved readily in three dimensions for large-scale harvesting of energy from nature (Section S18). It can be used anywhere, including in urbanized areas (e.g., rooftops). In comparison, hydroelectric power cannot harvest energy from rain directly, has severe requirements for its physical structure, is highly complex, and causes environmental damage. Hence, plug flow for harvesting renewable energy from nature is an effective and green source of electricity that has many advantages over other sources of alternative energy.

We found that the interfacial chemistry of plug flow is fundamentally different from streaming current even though both methods involve harvesting the charge separated constantly from the interface. Streaming current first requires establishing an electric double layer: an already-charged surface and a layer of free ions around the surface. The pressure-driven flow pushes the free ions across the length of the channel to the outlet. On the other hand, plug flow involves separation of charge only at the contact line of the receding edge of the discrete column of water. At the contact line, the separation of aqueous H^+^ and OH^–^ ions is due to the preferential adsorption of OH^–^ ions on the surface and preferential migration of H^+^ ions into the bulk water due to its higher mobility. Due to the different chemistries, both magnitude and polarity of charge generated by the two methods are different. Plug flow in a macroscale tube generates many orders of magnitude more electricity than theoretically possible for streaming current in a macroscale tube. Plug flow charges always positively regardless of the surface, whereas streaming current produces either positive or negative charge depending on the surface.

Streaming current is fundamentally limited by the Debye length because it requires the formation of the electric double layer. Plug flow is not limited by the Debye length because the electric double layer is not required. For plug flow, after separation of charge at the receding edge, the positive charge goes with the bulk liquid water down the tube and negative charge is left behind on the surface—the positive and negative charges are completely separated spatially. We found from our experiments that the charging efficiency is the highest when a single column of uncharged water passes only once through an initially uncharged tube. In this case, there is no negative charge at the interface between the bulk water and the surface (except the contact line at the receding edge). The positive charges are not constrained by the negative charges and are free to migrate in the bulk water; hence, the electric double layer is not present and plug flow is not limited by the Debye length. The complete spatial separation of the positive and negative charges gives rise to the large chemical potential of charge separation by plug flow.

We found that the fundamental principle to increase electricity generation is to introduce discontinuity. Discontinuity triggers the unique form of interfacial chemistry to occur as the charge separation happens only at the receding edge. We found that different extents of discontinuities provided by different types of flow patterns produce different amounts of power that range many orders of magnitude—thus, it is crucial to optimize flow pattern to increase electricity generation.

We found interestingly that switching fluid dynamics switches interfacial chemistry. We created a reversible flow transition that flexibly changes between continuous and discontinuous flow for switching the interfacial chemistry and generating the huge difference in power. This flexibility to change the interfacial chemistry is useful for applications: the continuous flow is useful for discharging surfaces (i.e., by carrying the charge away), whereas the discontinuous flow is useful for charging surfaces and liquids instead. Plug flow involves a unique form of charge transport: a counterflow of positive (downward) and negative (upward) charges across the length of the tube (32 cm) for separating charge effectively over large lengths. Charge needs to be separated at sufficiently large (e.g., macroscale) distances for tapping into the energy of the charge separated at the localized spot.

Flows of water with discontinuities and/or irregularities across surfaces are ubiquitous in nature and human activities. Hence, our results that involve the highly positively charged water and negatively charged atmosphere may provide the explanations for many unexplained observations of charged liquids (e.g., splashing) and charged atmospheres from nature and experiments reported in previous studies.^6,22,57–59^ Importantly, they explained the Lenard effect: the negative charge that is commonly detected in the atmosphere close to natural sources of flowing water, such as waterfalls, waves crashing onto shores, and rainstorms.^60^

## Methods

### Experimental Setup for the Plug Flow through a Tube

For systematic investigations of the phenomenon, we used a syringe pump for regulating carefully the flow of water. However, a pump was not necessary as described in the next section. A Terumo 3-Part 50 mL Luer Lock syringe (polypropylene) fitted with a stainless-steel gauge 18 needle (length, 15 cm; inner diameter, 0.838 mm) was mounted onto the syringe pump. The syringe was filled with deionized water. The stainless-steel needle had a sharp tip that tapered off asymmetrically on one side; the longest side of the tapered tip was 4 mm longer than the opposite shortest side. The needle was oriented horizontally. The tapered opening at the tip of the needle was oriented to face downward.

A FEP tube with a length of 32 cm and an inner diameter of 2 mm was used as the solid surface for contacting the flow of water. The tube was oriented vertically for allowing the water to flow down the tube naturally by its own weight. Half of the top 3 cm of the tube was cut vertically out; the remaining half of the top 3 cm of the tube provided the surface for the initial contact between the water and the solid tube. The tube was cleaned by rinsing it with ultrapure water. It was then dried by blowing the surface with a stream of argon gas and placed in an oven operated at 80 °C for 3 h. The tube was allowed to cool down at room temperature before use. The tube was measured to be uncharged (<0.005 nC/cm^2^) before use.

For generating power, the vertically oriented tube was fixed in position close to the tip of the metallic needle of the syringe. The tip of the needle was 1 cm vertically below the top of the tube and 1 cm horizontally away from the exposed inner wall of the remaining half of the FEP tube that the water first came into contact with. This configuration allowed air to be mixed into the flow for effectively generating the plug flow. When the flow of water comes out horizontally from the needle, it collides with the exposed inner wall of the half FEP tube. The impact of the collision between water and the solid surface breaks up the water flow. After the breakup of the flow, air tends to mix and trap inside the flow before the flow recombines in the tube, leading to the creation of the plug flow pattern. Then, 50 mL of deionized water was driven out of the needle by the syringe pump at a flow rate of 80 mL/min. The flow allowed the water to first come into contact with the inner wall of the remaining half of the tube; subsequently, the water flowed down the rest of the tube vertically after the initial contact. All experiments were conducted in the ambient atmosphere. The temperature was about 24 °C and the relative humidity was about 70%. The water that flowed out of the bottom of the tube was then collected by a stainless-steel cup placed below the FEP tube. Insulating materials were used to insulate the cup from the surroundings electrically. Before the experiment, the stainless-steel cup was filled with 10 mL of uncharged deionized water that was equivalent to ∼1 cm in height of water in the cup. One end of a wire was dipped into this water placed in the cup to maintain electrical contact (i.e., point (P1) as illustrated in Figures 1c and 1d). The other end of the wire was connected electrically to resistors (i.e., connected in series) with a total resistance of 61 GΩ, and then to ground. Another wire was connected to the metallic needle fixed on the syringe (i.e., point (P2) as illustrated in Figures 1c and 1d). Similarly, this wire was connected electrically to resistors (i.e., connected in series) with a total resistance of 41 GΩ, and then to ground. An electrometer (Keithley, model 6514) was used to measure the potential difference across a 1 GΩ resistor in each of these circuits. This method was verified to be able to accurately obtain the total voltage across the full set of resistors (Figure S10; Section S19). The electrometer was connected to a computer that allowed data acquisition to be done automatically through a LabVIEW program.

All the parameters of the experimental setup were optimized to achieve the highest power and efficiency. The parameters included the flow rate of water, length of tube, diameter of tube, resistances of the resistors used for both the cup at point (P1) and needle at point (P2), orientation of the needle, and distance between the needle and the tube. One critical point was the method by which the flow of water first contacted the tube. This optimized method was used to obtain the plug flow that consisted of water interspersed with air. Another important factor was the amount of resistance of the resistors connected to either the cup or the syringe needle. The resistance of the resistors connected to the cup was varied from 0 to 121 GΩ, whereas the resistance of the resistors connected to the syringe needle was varied from 0 to 81 GΩ. The results of these experiments are plotted in Figure 1g. The optimal power and efficiency were obtained when the resistance of the resistors connected to the cup was 61 GΩ and when the resistance of the resistors connected to the syringe needle was 41 GΩ. In addition, we found that the efficiency was optimal when the length of the tube was 32 cm. A smaller power was generated when the length was shorter; on the other hand, the power did not increase much beyond this length.

### Power Generation Using Water Towers

We used two types of simple makeshift water towers for harvesting the potential energy of water. The first water tower was a polyvinyl chloride (PVC) pipe (height, 150–165 cm; inner diameter, 8 cm) that had a custom-made cap that sealed the bottom end of the pipe (Figure 1d). A hole of 5 mm in diameter was made around the bottom of the pipe (i.e., 10 cm above the bottom end of the pipe); this hole served as the outlet for the water to flow out of the pipe. The second water tower consisted of six polyethylene terephthalate (PET) bottles joined together. These bottles were the typical 1.5 L plastic bottles that contained the bottled water commonly found in general stores. The bottles were cut and joined together by insulating tapes and silicone rubber (Ecoflex) to prevent water from leaking through the joints. After joining the bottles together, the water tower was 75 cm tall. A hole of 5 mm in diameter was made around the bottom of the joined bottles (i.e., 2 cm above the bottom end of the bottle); this hole served as the outlet for the water to flow out of the bottles. To direct the flow of water out of the towers specifically to the tube, the hole around the bottom of each of the two types of water towers was sealed with a connector; a metallic needle (length, 15 cm; inner diameter, 0.838 mm) was fixed onto the connector.

The hydrostatic pressure due to the water stored in the tower allowed the water to flow out of the tower, through the stainless-steel syringe needle at the outlet, and into the FEP tube. Because the flow of water out of the tower depended on the hydrostatic pressure, the flow rate was proportional to the height of the water level in the tower. Based on the heights of the water towers, the highest flow rate obtained from the tower made of the PVC pipe was 75–80 mL/min and the highest flow rate obtained from the tower made of the plastic bottles was 40 mL/min. The procedure for achieving the optimal flow pattern and optimal electric power was repeated as described in the previous section using these makeshift towers instead of using the syringe pump. The efficiencies were similar to that obtained by the syringe pump operated at the same flow rates (Figure S11). By using these towers, there was thus no need to rely on electricity (e.g., for operating the pump). Therefore, the harvesting of energy from the flow of water can be derived purely from natural sources of energy.

### Definition of Efficiency

We define the efficiency of the generation of energy (or power) in our system, ε_*ff*_, as the percentage of the electric energy generated divided by the loss in gravitational potential energy for a specific volume of water that flowed down the solid surface. We harvested the electric energy, *E*, via two sources in the experimental setup: (P1) the electric energy harvested from the electrical connection at the cup that collected the charged water after flowing across the solid surface, *E*_cup_, and (P2) the electric energy harvested from the electrical connection at the metallic syringe needle, *E*_needle_. On the other hand, the water lost gravitational potential energy, *P*, as it flowed down the whole tube vertically. This loss is expressed as *P* = *mgh*, where *m* is the total mass of water that flowed down the tube, *g* is the constant of gravitational acceleration, and *h* is the height of the tube. The efficiency of generation (i.e., in terms of either energy or power) can thus be expressed as eq 2.

S1 $$\varepsilon_{ff}=\frac{E}{P}=\frac{E_{cup}+E_{needle}}{P}=\frac{\int_{0}^{t_{f}}\left({V_{cup}\left(t\right)}^{2}/R_{cup}+{V_{needle}\left(t\right)}^{2}/R_{needle}\right)dt}{mgh}$$

where *t* is the time and *t*_*f*_ is the total duration of the experiment. *V*_cup_(*t*) and *V*_needle_(*t*) are the experimentally measured potential differences across the resistors of resistances *R*_cup_ and *R*_needle_, respectively, at the specific time *t*. Further discussion on the factors considered in this expression is in Section S3.

### Dripping-Rivulet Flow Transition Using a V-Shaped PTFE Channel

A polytetrafluoroethylene (PTFE) V-shaped channel was used for contacting the dripping flow or rivulet of water. The V-shaped channel was 50 cm long and 5 mm thick. Each of the two parts of the V-shape was 2.5 cm wide. The channel was cleaned and discharged by rinsing it with ultrapure water and ethanol, and then drying it by blowing a stream of nitrogen gas over the surface. The channel was fixed in position at an angle of 30° to the vertical. A syringe pump was used to regulate the flow of water down the channel for this controlled experiment. A 50 mL glass syringe was mounted onto the pump. It was fitted with a stainless-steel needle (length, 15 cm; inner diameter, 0.838 mm) and was filled with deionized water. The stainless-steel needle had a sharp tip that tapered off asymmetrically on one side; the longest side of the tapered tip was 4 mm longer than the opposite shortest side. The needle was oriented horizontally, and the opening of the tip faced upward. The needle was connected electrically to ground at (P2). The tip of the needle was placed close to the inclined surface of the V-shaped channel; it was pointing to, but without touching, the innermost surface of the valley of the V-shaped channel. The distance of separation between the tip of the needle and the innermost surface of the valley (at which the water first came into contact), *d*_sep_, was the parameter that we varied for obtaining the dripping-rivulet flow transition. When *d*_sep_ was very short at about 0.5 mm, we observed that the water flowed down the V-shaped channel as a rivulet. When *d*_sep_ was larger at about 2 mm, we observed that the water flowed down the V-shaped channel as a dripping flow. Section S9 discussed the fundamental mechanism behind this transition of flow patterns in more detail.

We used the pump to drive the water out of the needle at a constant flow rate of 15 mL/min. After the water flowed out of the needle, it flowed down the solid surface by gravity. After falling off the surface at the bottom end, the liquid was collected by an insulated stainless-steel cup placed at the bottom of the PTFE channel. The cup was connected electrically to multiple resistors at (P1) with a total resistance of 31 GΩ connected in series, and then to ground. An electrometer (Keithley, model 6514) was used to measure the potential difference across one of the resistors with a resistance of 1 GΩ at (P1). The electrometer was connected to a computer that allowed automatic data acquisition to be done automatically via a LabVIEW program.

We investigated the generality of the phenomenon by using different types of organic liquids instead of water. The organic liquids we used were dimethyl sulfoxide (DMSO), 1,3-dimethyl-2-imidazolidinone (DMI), and acetonitrile (ACN). The flow rates used were 1 mL/min for DMSO, 1 mL/min for DMI, and 5 mL/min for ACN. The same flow rates were used for each liquid for generating both the dripping flow and rivulet. For each flow rate, the liquid was pumped out of the syringe for 60 s.

## Supplementary Material













































## Data Availability

All data are available in the manuscript or the Supporting Information.

## Notes

Detailed description of the experimental methods of flow pattern control, charge measurements, XPS, FTIR, NMR, and ToF-SIMS analyses, and applications, supplementary discussions and figures for understanding the fundamental mechanisms, supplementary figures of generated powers and efficiencies of various experimental setups, list of materials used, and additional photos of the experimental setup (PDF)
Transparent Peer Review report available (PDF)
