# Supplementary material for: Plug Flow: Generating Renewable Electricity with Water from Nature by Breaking the Limit of Debye Length
Source: ACS Cent Sci. 2025 Apr 16;11(5):719–33. doi: 10.1021/acscentsci.4c02110 (PMC12164936; doi:10.1021/acscentsci.4c02110)
Supplement: Supplementary file 1 [file oc4c02110_si_001.pdf]

Supporting Information for

Plug Flow: Generating Renewable Electricity with Water from Nature by Breaking the Limit of Debye Length

Chi Kit Ao, Yajuan Sun, Yan Jie Neriah Tan, Yan Jiang, Zhenxing Zhang, Chengyu Zhang, and Siowling Soh*

Department of Chemical and Biomolecular Engineering, National University of Singapore, 4 Engineering Drive 4, Singapore 117585, Singapore

* To whom correspondence may be addressed: chessl@nus.edu.sg

The PDF file includes:

Detailed description of the experimental methods of flow pattern control, charge measurements, XPS, FTIR, NMR, and ToF-SIMS analyses, and applications, supplementary discussions and figures for understanding the fundamental mechanisms, supplementary figures of generated powers and efficiencies of various experimental setups, list of materials used, additional photos of the experimental setup.

Other Supporting Information for this manuscript includes the following:

Movie S1 to 10

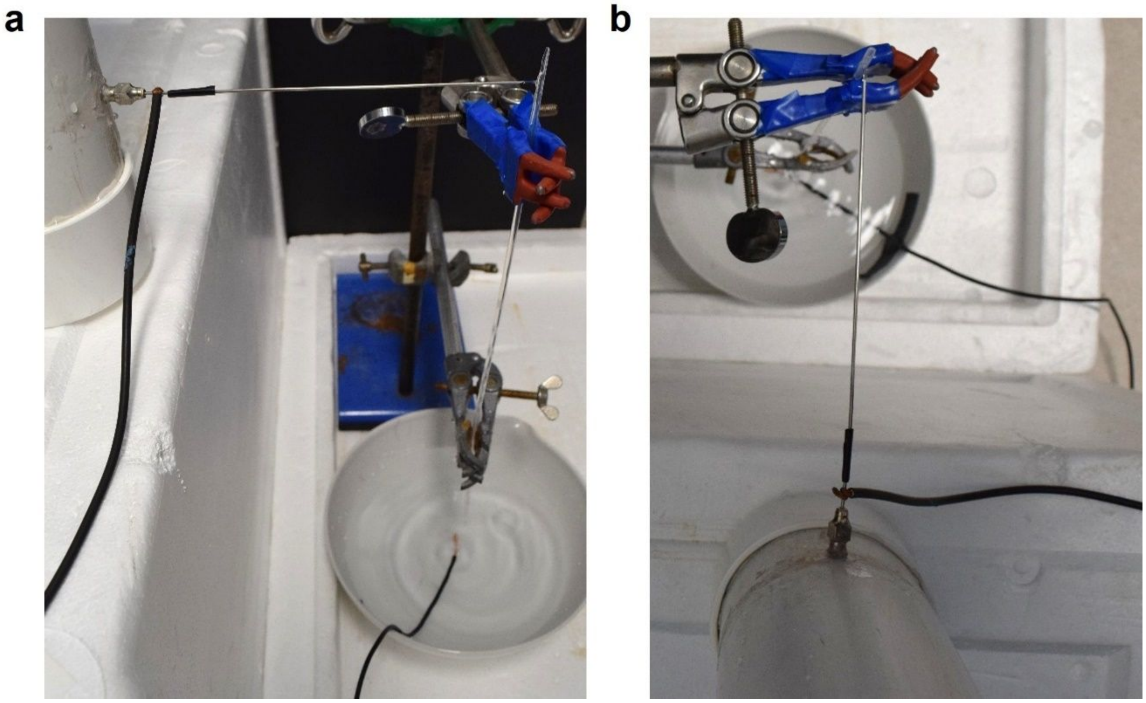

Figure S1. (a) Side view and **(b)** top view of the experimental setup for generating electricity by the plug flow of water.

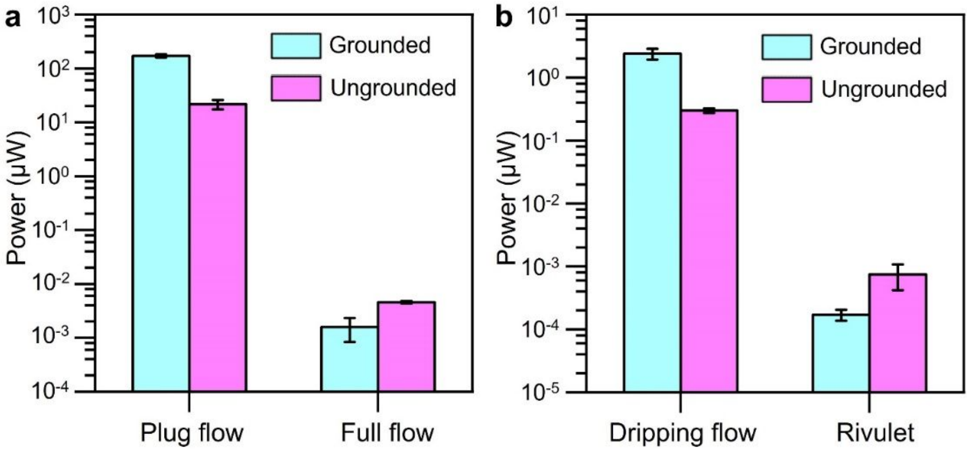

Figure S2. Comparison of the power generated when the metallic syringe needle at (P2) was electrically grounded or not grounded. Power obtained from flowing water down a **(a)** FEP tube with an inner diameter of 2 mm or **(b)** PTFE V-shaped channel. For each type of solid surface, two types of flow patterns (i.e., "Plug flow" and "Full flow" for **(a)**, and "Dripping flow" and "Rivulet" for **(b)**) were examined. These results showed that grounding (P2) or not did not affect the conclusion that changing flow pattern has a large effect on the generation of electrical power. The error bars represent the standard deviation.

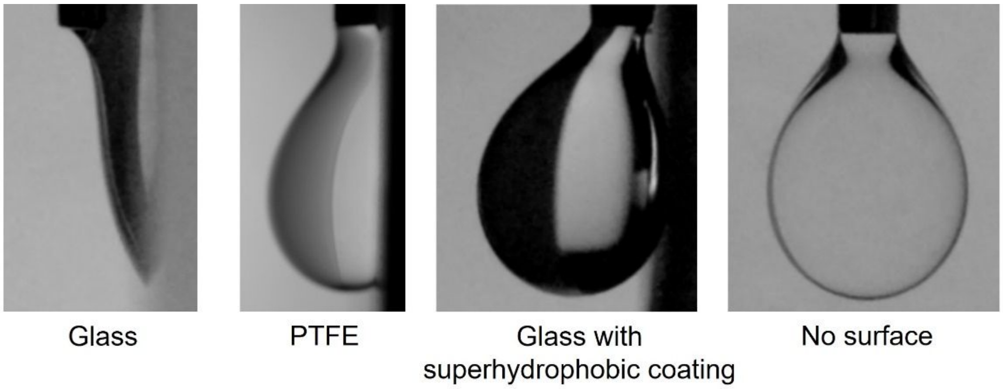

Material	Glass	PTFE	Glass with superhydrophobic coating	No surface
Contact angle (°)	30	100	150	180
Volume of detached drip (μL)	10.2	14.2	16.0	16.7

Figure S3. Fundamental mechanism of the dripping-rivulet flow transition. Images showing water drips hanging at the tip of a flat syringe needle either not close to any surface or close to surfaces of solids (i.e., glass, PTFE, or glass coated to be superhydrophobic). These images were taken at the moment just before the water drips detached from the needle as more water pumped gradually out of the syringe needle. The table at the bottom shows the volumes of the drip at the moment when they detached from the syringe needle (i.e., corresponding to the images on top) and contact angles of water on the different types of surfaces (i.e., measured when the surfaces were oriented horizontally). These results showed that the water has the tendency to spread onto surfaces. The contact with the surface allows the water drips to detach easier — with less volume — from the tip of the needle than when the water is not in contact with any surface.

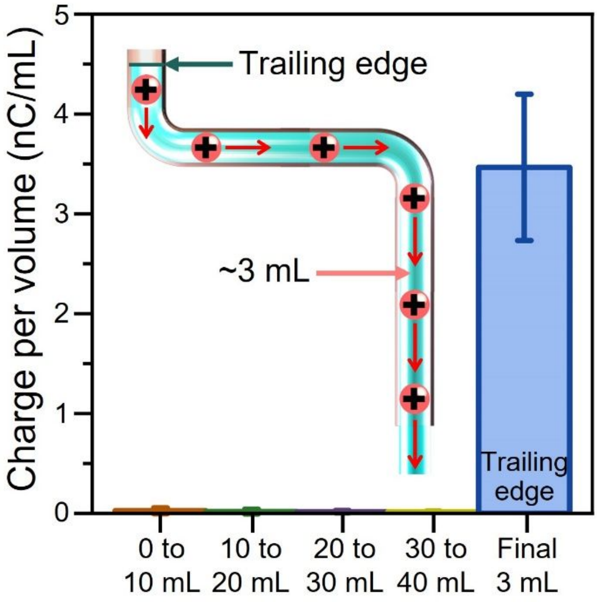

Figure S4. Charge per volume of different portions of a long discrete column of water that flowed through a 2 mm FEP tube. This experiment involved grounding electrically the connector between the syringe and the FEP tube. Figure 5g in the main text refers to the experiment in which the connector between the syringe and the FEP tube was insulated and not grounded. Charge was generated only when the trailing edge appeared. The conclusions of the three experiments showed in Figure 5g in the main text, this figure, and Figure S5 are the same. The error bars represent the standard deviation.

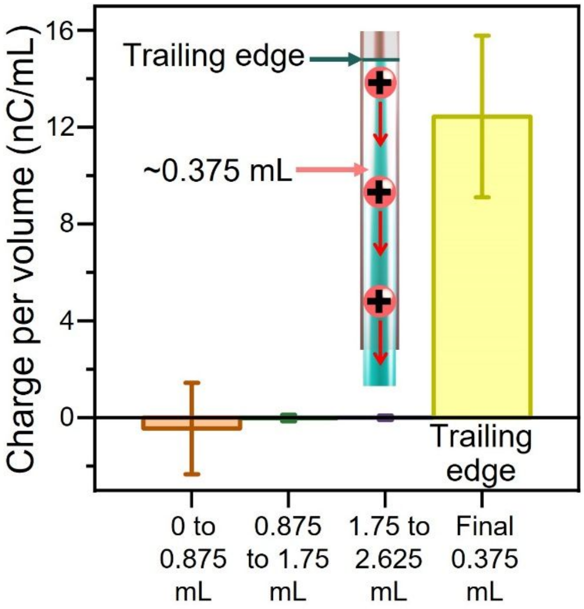

Figure S5. Charge per volume of different portions of a long discrete column of water that flowed through a 1 mm FEP tube. The connector between the syringe and the FEP tube was electrically insulated and not grounded; thus, this setup was the same as that reported in Figure 5g of the main text. Charge was generated only when the trailing edge appeared. The conclusions of the three experiments showed in Figure 5g in the main text, Figure S4, and this figure are the same. The error bars represent the standard deviation.

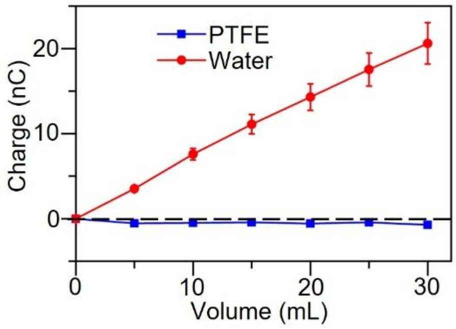

Figure S6. Negative charge did not accumulate substantially on the solid surface. An experiment was conducted in which a discontinuous flow was allowed to come into contact with a single piece of PTFE and fall off the surface one after another sequentially. Plot shows the measurements of the charge of the piece of PTFE and accumulated charge of the discontinuous flow of water after contacting the surface of PTFE. After flowing 30 mL of water across the solid surface, the total charge of the water accumulated to a positive amount of around +20 nC. On the other hand, the charge of the PTFE quickly saturated at a negative charge of only around -1 nC after coming into contact with a small volume of water. Hence, the piece of PTFE did not charge significantly even as the flow of water continued to charge highly positively. The error bars represent the standard deviation.

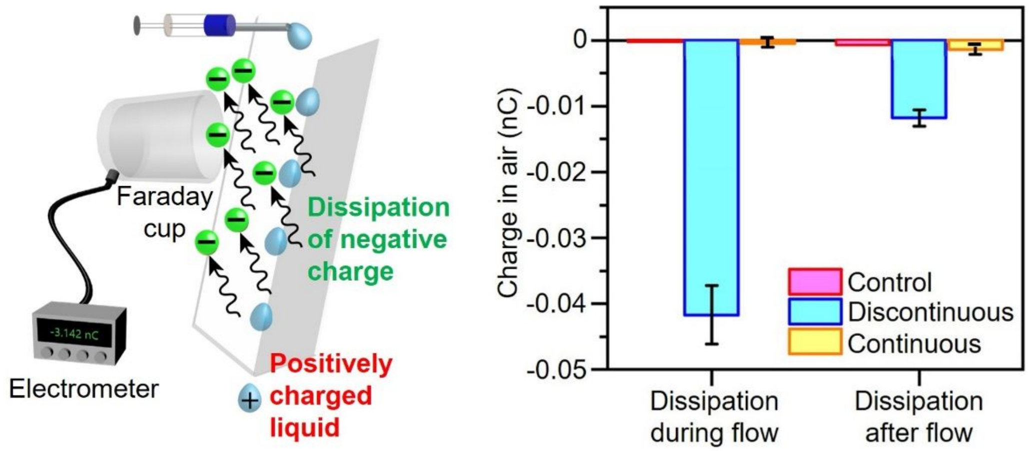

Figure S7. Negative charge dissipating into air by the flow with discontinuity. Scheme on the left illustrates the experimental setup for detecting the negative charge in the atmosphere surrounding the flow. Plot on the right shows the measurement of charge in the atmosphere when there was no flow (“Control”), discontinuous flow (“Discontinuous”), or continuous flow (“Continuous”) for two cases: during flow (“Dissipation during flow”) or after the flow stopped (“Dissipation after flow”). The error bars represent the standard deviation.

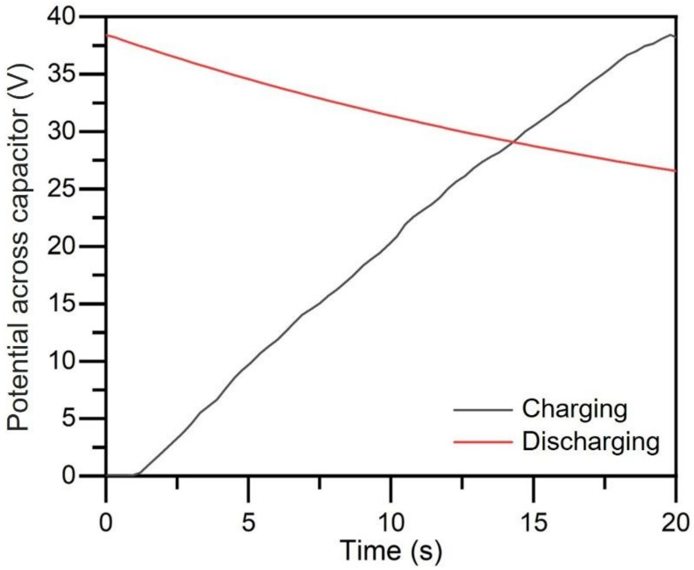

Figure S8. Electricity generated by plug flow powered the lighting of LEDs continuously by charging and discharging capacitors. The plot showed the potential difference across one of the capacitors. For 20 s, the water flow charged up the capacitor to close to 40 V. When used to light up the 12 LEDs for 20 s, the capacitor discharged from close to 40 V to around 26 V. Hence, the capacitors had two-thirds of their charge remained stored even after lighting up the 12 LEDs continuously for 20 s.

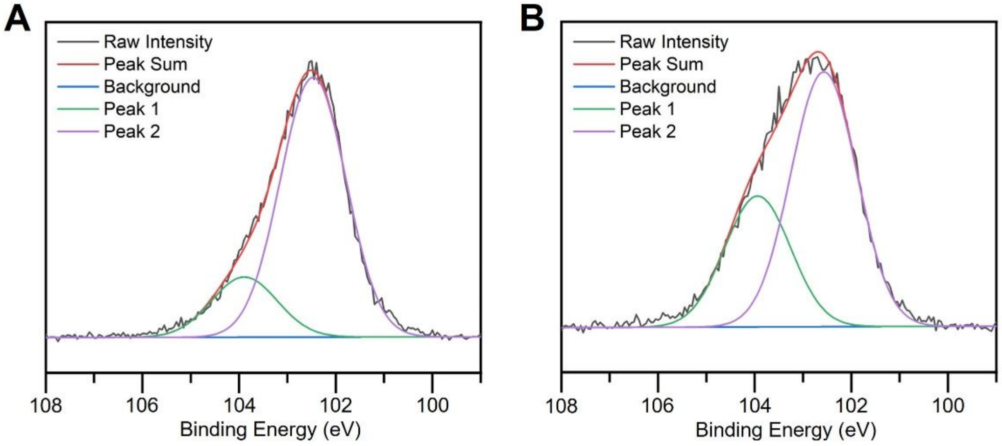

Figure S9. High resolution XPS spectra of Si 2p of the surfaces of PDMS. (A) before and **(B)** after treatment by the electric power generated by the plug flow of water down a FEP tube. This result suggested that the chemical mechanism involved first the cleavage of the Si-CH_3 bonds on the surface of PDMS and then the formation of the hydrophilic Si-OH groups that replace the Si-CH_3 groups. The hydrophilic Si-OH groups allowed the wettability of PDMS to be increased.

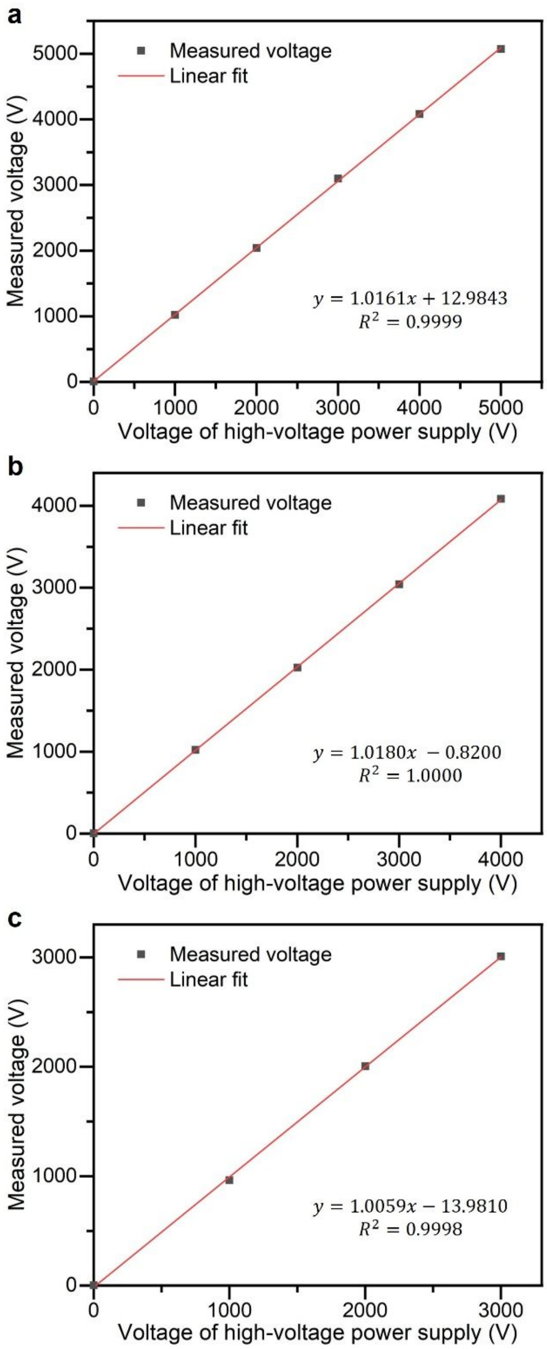

Figure S10. Calibration of the electrical circuit. A high-voltage power supply was used to pass electricity through resistors with a total resistance of **(a)** 61 GΩ, **(b)** 41 GΩ, or **(c)** 31 GΩ and then to ground. The potential difference across the resistors supplied by the plug flow of water (*y*-axis) matched the potential supplied by the high-voltage power supply (*x*-axis).

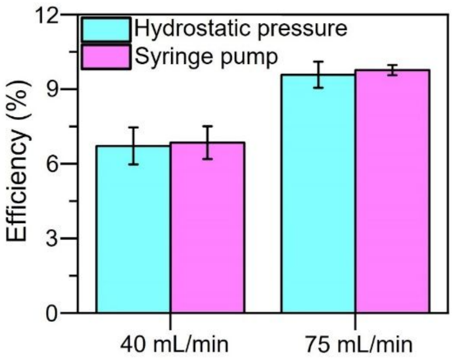

Figure S11. Highly effective charging by the optimal plug flow obtained by an extremely simple experimental setup. Comparison of efficiencies by using the water tower ("Hydrostatic pressure") and syringe pump operated at the same flowrates. The error bars represent the standard deviation.

	F%	C%	O%
FEP tube before flowing water	63.8	35.7	0.5
FEP tube after flowing water	63.6	35.8	0.6

Table S1. Compositions of fluorine, carbon, and oxygen on the surfaces of FEP tubes before and after flowing water across them by the elemental quantitative analysis of XPS. The results showed that the tubes were made of fluorinated ethylene propylene (FEP). The chemical composition of the tube did not change after extensive amounts of water flowed through it.

Flow pattern	Efficiency (%)	Power (μW)	Power/Power by plug flow
Plug flow	4.0	170	1
Plug-dripping flow	1.5	63	0.4
Dripping flow	0.40	17	0.1
Dripping flow (10 cm)*	0.13	1.7	0.01
Rivulet (10 cm)*	0.027	0.35	2×10^{-3}
Full flow	3.8×10^{-5}	1.6×10^{-3}	9×10^{-6}
Control (no tube)	4.0×10^{-6}	1.7×10^{-4}	1×10^{-6}

Table S2. Power generated and efficiency at (P1) by different flow patterns. All the setups involved tubes of 32 cm in length, except for the two rows indicated by the asterisks *. In these cases, tubes that had a length of 10 cm were used. The reason for using a shorter tube was because it was the maximum length that the rivulet could be obtained robustly. A longer length of the tube led to the Plateau–Rayleigh instability that had the tendency to break up the rivulet into discrete drips. For comparing with the rivulet, the experiment involving the dripping flow was performed using also the shorter tube of 10 cm. This comparison using the tubes of the same length showed that the power and efficiency generated by the dripping flow were much greater than that of the rivulet.

	C	N	O	Si
Uncoated glass after piranha treatment	7.0%	0.7%	54.3%	38.0%
PEI-coated glass	30.1%	6.2%	37.7%	26.0%
PDDA-coated glass	24.9%	2.0%	42.5%	30.6%
PAH-coated glass	19.6%	2.6%	47.5%	30.3%

Table S3. XPS analysis of the chemical composition of the surface of glass uncoated or coated with PEI, PDDA, or PAH. The analysis showed larger amounts of nitrogen compared to the surface of the bare uncoated glass, thus indicating the successful coating of the polyelectrolytes onto the glass surface.

	C	S	O	Si
Uncoated glass after piranha treatment	12.7%	0.3%	52.7%	34.3%
PSS-coated glass	26.0%	5.0%	43.3%	25.7%

Table S4. XPS analysis of the chemical composition of the surface of glass uncoated or coated with PSS. The analysis showed a larger amount of sulfur compared to the surface of the bare uncoated glass, thus indicating the successful coating of PSS onto the glass surface.

	Water contact angle (°)
Uncoated glass after piranha treatment	5.3 ± 2.0
PEI-coated glass	44.5 ± 1.7
PDDA-coated glass	31.6 ± 2.1
PAH-coated glass	55.8 ± 2.4
PSS-coated glass	21.2 ± 0.3

Table S5. Contact angle of water on glass surface uncoated or coated with polyelectrolytes. The significant differences in the contact angles between the uncoated and coated surfaces showed that the surfaces were coated.

Supplementary Text

S1. Analyzing the Chemical Composition of the Tube Before and After Flow

We analyzed the chemical composition of the fluorinated ethylene propylene (FEP) tubes that we purchased commercially and used in our experiments for generating power before and after flowing water. The surfaces of the tubes were analyzed by Fourier-transform infrared spectroscopy (FTIR), X-ray photoelectron spectroscopy (XPS), and Time-of-Flight Secondary Ion Mass Spectromety (Tof-SIMS). The FTIR spectra were recorded by a Vertex 70 spectrometer (Bruker, USA) using the Attenuated Total Reflectance (ATR) mode. The XPS spectra were recorded on a Kratos AXIS Ultra^{DLD} system (Kratos Analytical, UK) with Al Kα excitation radiation (1486.71 eV). The pressure in the analysis chamber was maintained at 10^{-6} Pa during the measurement. All spectra were referenced to the C 1s hydrocarbon peak at 285.0 eV to compensate for the effect of surface charging. ToF-SIMS analysis was carried out using a Time-of-Flight Secondary Ion Mass Spectrometer (ToF-SIMS 5 IONTOF) equipped with a Bi_1^+ liquid-metal primary ion source. Primary ion bombardment was done by 30 keV Bi_1^+ ions with a pulsed current of 1 pA. An analysis area of 100 × 100 μm with raster pixels of 128 by 128 was scanned and at least three different spots were analyzed. The total number of cycles for acquisition was fixed at 200. Electron flood gun was used to compensate the positive charging of the FEP sample by the primary ion beam.

Results from the analysis by FTIR showed the characteristic CF_2 symmetric stretching peaks at 1201 and 1146 cm^{-1}, C-CF_3 side chain stretching peak at 982 cm^{-1}, CF deformation peaks at 637 and 627 cm^{-1}, and CF_2 bending peaks at 554 and 511 cm^{-1} (Figure 5d).[1, 2] Besides these signals, we did not obtain any other signal (e.g., no C-O stretching bond was detected). XPS detected only C 1s (285 eV, 293 eV) and F 1s (690 eV), together with a negligible amount

of O 1s (532 eV) (Figure 5e). The peak at 293 eV from the C 1s spectrum represents the C-F bond. The peak at 690 eV from the F 1s spectrum also represents the C-F bond. The peak at 285 eV from the C 1s spectrum may represent the C-C bond of the material and/or any adventitious carbon on the surface. Quantitative elemental analysis of the surfaces was also performed by XPS. The elemental ratio of C to F is around 1.8 (Table S1); this ratio is commonly reported when solids made of FEP or PTFE are analyzed by XPS.[3, 4] Based on the results from all these analyses, we can conclude that the tubes we used for generating power are made of the FEP polymer.

We further determined whether the chemical composition of the tube changed after flowing water through it and drying it. For this analysis, we flowed an extensive amount of water through the tube and kept it in a humid condition for 3 days. We then dried the tube by a stream of argon gas for 1 min and analyzed its chemical composition by FTIR and XPS. The results from FTIR and XPS showed that there is no difference between the tubes that had water flowed through them or not.

We performed ToF-SIMS for three cases: before flowing water, after plug flow, and after full continuous flow. For comparing the intensities of the peaks across different spectra from different cases, normalization of the intensities was performed to eliminate the systematic differences generated by ToF-SIMS for each separate analysis.[5] The intensities of the peaks were normalized by the intensity of the peak CF^- (m/z = 31). We considered the intensity of the peak CF^- as the normalization factor because it is the main chemical moiety in FEP. For the normalization, we divided the intensities of all the peaks in the spectrum by the intensity of the peak CF^- for comparing the intensities of the peaks across different analyses.

The results of the analyses showed that the normalized intensity of OH^- on the inner surface of the FEP tube after flowing plug flow was strong. Negligible intensities were found for the surface before flowing water and the surface after full continuous flow. The trend was clear and remained the same using other normalization factors such as F^- (m/z = 19).

S2. Decrease in Efficiency with Increasing Load Resistance Beyond Optimal Resistance

As described in the main text, there is an optimal efficiency of power generation when we vary the load resistance of the external circuit connected to point (P1) and point (P2) (Figure 1f). We found from our experiments that the efficiency decreases when the resistance increases beyond around 40 – 60 GΩ. The decrease in efficiency after the optimal load resistance seems to prevent the system from attaining a higher maximum efficiency of power generation from the system.

On the other hand, the resistance of 40 – 60 GΩ is very high. It is typically challenging to work with systems with high load resistance as dissipation and/or recombination of charge becomes largely inevitable. Hence, the decrease in power when load resistance is high is a widely reported phenomenon in previous studies that discuss power-generating systems. For example, a decrease in power when load resistance is high is described in devices based on electrostatic induction (i.e., triboelectric nanogenerators)[6-10] and systems based on streaming current.[11] In this section, we first discuss three observations that we made while performing experiments to explain the phenomenon.

The first observation involves measuring the charge in the cup after collecting the charged water that just flowed out of the FEP tube. When the cup was connected to an external circuit (i.e., at point (P1)) with a low load resistance, we found that the water in the cup was not charged; the charge in the water flowed out of the cup rapidly as soon as it was collected due to

the low resistance of the external circuit. However, when we increased the load resistance to around 40 – 60 GΩ (i.e., when the maximum efficiency occurs), we found that (positive) charge accumulated in the cup. The experiment involved the typical setup, including flowing a plug flow of water at a rate of 80 mL/min through a 2 mm diameter FEP tube and connecting the point (P1) to an external circuit with a specific resistive load. (P2) was electrically grounded. In addition to the typical setup, we placed a Faraday cup connected to an electrometer 10 cm below the outlet of the FEP tube. A 1000 mL glass beaker was placed inside the Faraday cup for collecting the water that flowed out of the tube. For this experiment, we flowed a total of 50 mL of water through the tube and into the beaker in the Faraday cup. Flowing the charged water into the Faraday cup enabled us to measure the charge of the water in the beaker. The first experiment involved using a small resistive load of 1 GΩ (i.e., a relatively low resistance compared to 40 – 60 GΩ when we obtained the maximum efficiency). In this case, we measured only a negligible amount of charge of 0.25 nC in the glass beaker when the water flowed into the glass beaker. When the load resistance was increased to 61 GΩ (i.e., when the maximum efficiency occurs), charge of 15 – 20 nC was measured. Hence, these results showed that when the resistance of the load was small, charge flowed out readily from the glass beaker; hence, there was no charge in the beaker. On the other hand, when the resistance was high, charge accumulated in the cup as it was difficult to flow out of the external circuit.

The second observation is that charge of the charged water in the cup dissipates spontaneously and rapidly even when the cup is not connected electrically to any external circuit. The experiment involved the typical setup, including flowing a plug flow of water at a rate of 80 mL/min through a 2 mm diameter FEP tube. In this experiment, however, the water that flowed out of the FEP tube into the cup (i.e., point (P1)) was not connected to an external circuit. In

addition, to eliminate any undefined conductive paths that may lead to dissipation of charge in the glass beaker, we performed a controlled experiment in which we placed a 1000 mL glass beaker into a thick electrically insulating Styrofoam cavity. The glass beaker was under the outlet of the FEP tube for collecting the charged water that flowed out of the tube. We flowed 50 mL of water through the tube and into the glass beaker and immediately measured the charge of the water using a Faraday cup connected to an electrometer. We measured that the initial charge of water in the beaker was about 500 nC. We then waited 20 s and measured the charge of water in the beaker again. We found that only ~30 nC remained in the beaker after 20 s. This result showed that charge in the water in the cup dissipates into the surrounding readily and rapidly even when the water is not connected to an external circuit. This result suggests that due to the high resistance of the resistive load at maximum efficiency, charge in the water finds other routes to dissipate simultaneously in addition to flowing through the external circuit (e.g., via solid surfaces even for insulating materials due to the high moisture involved in our experiment).

The third observation is that we measured the dissipation of charge from the charged water into air in our manuscript (e.g., Figure S7). Hence, charge may dissipate both through the solid surfaces and into air.

Another explanation is that when the positive charge accumulates in the cup, it may recombine with the negative charge present in the system. For streaming current, it is well established that a maximum efficiency for generating power is reached when the external load resistance matches the electrical resistance of the bulk liquid.[12-14] Increasing the load resistance further causes the generated charge to flow back into the system and recombine with the opposite charge in the system instead of flowing out of the system. In our case, the accumulation of the positive charge in the cup (i.e., instead of flowing out of the system due to the high load

resistance) produces a large electric potential for flowing back into the system (e.g., back into the tube) and recombining with the negative charge present in the system. This recombination of positive and negative charges results in a lower efficiency of power generation with increasing load resistance. To conclude, the decrease in power due to high load resistance is a general phenomenon that occurs in many systems and is not particular to our system. With a high resistive load, dissipation of charge via other routes becomes inevitable (e.g., through surfaces or air) and recombination of charge becomes significant.

S3. Energy Balance of the System for Power Generation

We obtained the optimal power and efficiency based on the experimental setup that involved the plug flow of water at a flowrate of 80 mL/min through a FEP tube (i.e., a diameter of 2 mm and a length of 32 cm). The efficiency is defined mathematically in Methods. This definition of the efficiency of the system requires the consideration of the different types of energies that flow into and out of the system. This section discusses the energy balance of the system — in particular, we discuss the involvement (or the lack) of the kinetic energy of water that flows into and out of the system.

We first define our system. As discussed in Methods, the vertically oriented tube had two distinctly different sections. The first section was the top 3 cm of the tube. After cutting vertically out half of the tube, the remaining half of the tube served as the receiving end for allowing the stream of water that flowed out of the syringe needle to come into initial contact with the surface of the tube. The second section was the rest of the tube below the top 3 cm that remained full and uncut. To understand the significance of the top 3 cm of the tube, we prepared a surface that was composed of only the top 3 cm of the tube that was cut vertically into half; that

is, there was no more tube below this top section of 3 cm. After flowing water through this first section of the tube, we found that the power generated was negligible; hence, the top 3 cm of the tube did not contribute to the generation of power. Therefore, we consider our system for the generation of electric power to be only the second section that involves the full tube.

Although kinetic energy flows through the system, we determined that its contribution to the generation of electric power is negligible. To understand its contribution, we determined the amount of kinetic energy that flowed through the system by measuring the velocities of the flow of water into and out of the full tube experimentally. The experiment involved driving the flow of water by a pump operated at the rate of 80 mL/min. The FEP tube used had a diameter of 2 mm and a length of 32 cm. The water flowed down the tube that was oriented vertically. Videos of the flow through the full tube were taken by a Nikon D5300 camera equipped with a Nikon AF-S DX Nikkor 18-140 mm f/3.5-5.6G ED VR lens and operated at a rate of 50 frames per second. Using this setup, we measured the velocity of water just before it flowed into the full tube and the velocity of water just after it flowed out of the full tube. The velocities were determined by analyzing the distance traveled by the water between the frames of the videos using an image processing software (ImageJ; http://imagej.nih.gov/ij/). From this experiment, we found that the velocity just before it flowed into the full tube was ~0.35 m/s, and just out of the full tube was ~0.4 m/s.

The kinetic energy of water through the system can be neglected based on two main reasons. First, we found that the amount of kinetic energy is negligible compared to the amount of gravitational potential energy of the water. The kinetic energy of the flow of water at the inlet of the full tube is $K_{in} = \frac{1}{2}mv^2$, where m is the total mass of the water that flows through the tube and v is the velocity of the flow. The gravitational potential energy lost by the water as it flows

down the full tube vertically is $P = mgh$, where g is the constant of gravitational acceleration and h is the height of the tube. For comparing the two amounts of energies, we can take their ratio: $\frac{K_{in}}{P} = \frac{\frac{1}{2}v^2}{gh}$. Because v = 0.35 m/s and h = 32 cm, the ratio is only ~0.02. This analysis showed that the kinetic energy was negligible compared to the gravitational potential energy. Second, the velocity at the outlet of the tube (~0.4 m/s) is actually slightly higher than that at the inlet of the tube; this increase in velocity is presumably due to gravity. Because there is no loss in kinetic energy in the system, the electric energy was generated purely by the gravitational potential energy only. Because the kinetic energy is maintained at a similar amount as it flows in and out of the system, it can subsequently be used for flowing water into another tube for further generation of power in a second stage. In addition, we performed a control experiment in which we oriented the tube horizontally instead of vertically. To facilitate the flow, the tip of the syringe needle was placed 5 mm inside the inlet of the full tube. For this experiment, we measured the velocity of the water through the tube and found that it was similar to that when the tube was oriented vertically at ~0.4 m/s. However, the flow was observed to be a full flow (i.e., the flow filled the whole tube without any air observed). The power measured was negligible at 5×10^{-5} μW. Hence, the kinetic energy was not responsible for the generation of power. Because kinetic energy can be neglected, we did not include it in our calculation of the efficiency of the power generation.

S4. Mechanism of Continuous Power Generation by Discontinuous Plug Flow

In the main text, we described that the power generated from our system was continuous. We measured experimentally that the electricity produced was continuous with time without any interruptions (i.e., breaks with no current). On the other hand, the flow pattern (i.e., plug flow)

was discontinuous. In this section, we studied the reason for the production of continuous power from a discontinuous flow pattern.

When charged water flows out of the tube, we obtain electricity. However, plug flow consists of alternating short columns of water and air. When it is time for air to flow out of the tube, we probably should not get any electricity. We calculated the average duration of air that flows out of the tube in between two charged columns of water. After quantifying the plug flow in our system experimentally, we found that the average height of a single column of air in the tube was around 5 mm. The velocity of flow inside the tube (for both water and air) was around 0.35 m/s. Therefore, we calculated that the duration of air that flows out of the tube per air gap in between two columns of water is ~0.015 s. This is a very short time of air flowing out during which there should be no electricity generated.

Subsequently, we examined the flow of electricity after we stopped the plug flow. In this experiment, we used the typical setup for obtaining the optimal electricity, including having a resistive load of 61 GΩ at (P1) and 41 GΩ at (P2). We flowed a finite amount of water (i.e., 50 mL in this case) through the tube until there was no more water. At the same time, we measured and monitored the electricity generated continuously. Our results showed that when the water stopped flowing in the system, the generation of electricity did not stop instantaneously; instead, we found that the electricity decreased gradually with time (see Figure 2a for (P1) and Figure 2b for (P2) in the main text). The results showed that electricity continued to flow even after a relatively long time, on the order of magnitude of ~1 s. This time is relatively much longer than the time when air flows out of the tube and no electricity is generated (i.e., ~0.015 s). Therefore, the electricity can be continuously supplied.

To gain a fundamental understanding of the continuous power, we examined why the electricity did not stop flowing even after water stopped flowing out of the tube for ~1 s. Based on our experimental results, it seemed that it was due to the high resistive loads at (P1) and (P2) that allowed charge to remain in the system for some time before flowing out of the system. To test this mechanism, we investigated different amounts of resistive loads at (P1) and (P2). Our experimental results showed that the duration that electricity continued to flow decreased with decreasing resistive loads. When no resistors were connected at (P1) and (P2) (i.e., both (P1) and (P2) were connected directly to ground), the flow of electricity stopped instantaneous at <0.1 s after the water stopped flowing in the system. These results indicated that due to the high resistive loads used, charge remained in the system and did not flow out immediately. When the water stopped flowing, the charge that remained in the system then gradually flowed out and caused electricity to continue to flow. The supply of electricity from the system fluctuated with time possibly due to this phenomenon but remained continuous throughout time.

We further provide an order-of-magnitude analysis of the amount of charge remaining in the system that drives the continuous flow of electricity even when air was flowing out of the tube instead of charged water. We first assume that the rate of charge generated by our system per unit time is constant at steady state (i.e., due to the same chemistry of charge separation across a constant length of the tube over time). For this analysis, we performed experiments and found that there was more charge per unit time that flowed out of the system when the resistive load was reduced. At (P1), when the resistive load was 61 GΩ, the rate of charge that flowed out of the system was ~70 nC/s. When the resistive load was only 1 GΩ, the rate of charge that flowed out of the system was ~110 nC/s. If we consider 1 GΩ to be negligible (i.e., relative to the optimal resistance of 61 GΩ) for this discussion, we could conclude from this experimental

result that the system inherently was able to generate charge at a rate of 110 nC/s. On the other hand, we only obtained ~70 nC/s when a high resistive load was used. Therefore, based on our assumption of constant rate of charge generation in the system, the difference of around 40 nC/s is the rate of charge that remained in the system due to the high resistive load.

We compare this rate of charge that remained in the system with the rate of charge needed to continuously supply electricity even when air is flowing out of the system instead of charged water. Each column of air flowed out of the tube with a short duration of ~0.015 s. There were roughly on average 15 columns of air in one second. Therefore, there was around 0.2 s of air flowing out of the system per second. The rate of charge typically flowing out of the system with the optimal high resistive load was ~70 nC/s. Hence, the rate of charge needed to continuously supply electricity at the same rate was around 15 nC/s. Compared to the 40 nC/s remained in the system, there is thus sufficient amount of charge in the system for supplying electricity continuously.

S5. Measuring Charge Generated by Plug Flow

To show that the electricity generated by our system was harvested directly from the separation of charge at the solid-liquid interface, we measured the charge generated by the system directly. The experiment involved a plug flow of 5 mL of deionized water through a 2 mm FEP tube at a flowrate of 80 mL/min. After flowing through the tube, the water was collected by a Faraday cup, which was connected to an electrometer for measuring the charge of the water. No external circuit was connected to the water (i.e., (P1)) collected in the Faraday cup. A resistive load of 41 GΩ was used at (P2) (i.e., the same resistive load used at (P2) for generating the optimal efficiency). Results showed that the charge per unit mass of water collected in the Faraday cup

was measured to be around 60 nC/g. On the other hand, when we used the typical experiment for generating power at the optimal efficiency, we could convert the current generated into charge per unit mass of water at 62 nC/g. Therefore, the charge measured directly by the Faraday cup at 60 nC/g matched the amount generated by the typical experiment for generating optimal power. We thus showed that the power generated was due to the charge generated by the plug flow.

S6. Highly Efficient Power Generation under Different Environmental Conditions

In the main text, we showed that our system can generate power with an optimal efficiency of 10.4% using a plug flow of deionized water with a flowrate of 80 mL/min through a FEP tube. We investigated the influences of different environmental conditions on efficiency, including flowrate, water quality, and temperature of water.

To study the influence of flowrate on efficiency, we performed experiments with four different flowrates: 20 mL/min, 40 mL/min, 60 mL/min, and 80 mL/min. We found that more than 90% of the optimal efficiency of the system can be achieved within a large range of flowrates from 40 mL/min to 80 mL/min (Figure 2c in the main text).

To study the influence of quality of water on efficiency, we performed experiments with different types of liquids, including deionized water (i.e., the typical case used in our study), tap water, and solutions of sodium chloride (NaCl) with a concentration of 0.1 mM or 10 mM. Results showed that the experiments using tap water and NaCl solutions had more than 85% of the optimal efficiency of the system using deionized water (Figure 2d in the main text). Although adding a small amount of salt reduced efficiency by a bit, adding more salt did not reduce the efficiency further.

To study the influence of temperature on efficiency, we performed experiments with three different temperatures of water: 4°C, 25°C (typical case), and 50°C. These temperatures cover the typical range of temperatures of water in practical settings. Results showed that the effect of different temperatures was not statistically significant (Figure 2e in the main text). The pairwise p-values from Student's t-tests of 4°C and 25°C, 25°C and 50°C, and 4°C and 50°C were 0.782, 0.487, and 0.507 respectively. These p-values were all much higher than the standard p-value of 0.05. Hence, the efficiencies generated by experiments using water at different temperatures were statistically similar.

These experiments showed that the system provided consistently high efficiency of power generation under different conditions.

S7. High Efficiency Maintained After Repeated and Prolonged Use

The ability of an energy-harvesting system to operate consistently for long periods of time is important in practical applications. To test whether our system could perform well after repeated use, we performed two experiments as follows.

The first experiment involved flowing plug flows of water continuously for different durations of 1 min, 1 h, or 2h. The experiment involved using the water tower of 1.65 m. The water tower was filled with water and the water was allowed to enter an FEP tube (inner diameter: 2 mm and length: 32 cm) at a flowrate of 75 mL/min (i.e., same flow rate used in Figure S11). We used a resistive load of 61 GΩ at (P1) and 41 GΩ at (P2). Results showed that the amounts of power generated at (P1) and (P2) were similar for all three durations of flowing water from the water tower (Figure 2f in the main text). Hence, the overall efficiencies of the system combining the power generated at (P1) and (P2) were similar for all durations.

The second experiment involved flowing plug flows of water at a flowrate of 80 mL/min through the same FEP tube (inner diameter: 2 mm and length: 32 cm) five times each day, 1 min each time, for seven days in a row. We used a resistive load of 31 GΩ at (P1) and grounded (P2). After each run, compressed air was blown through the FEP tube for 10 s to remove any stuck water in the tube. Results showed that the efficiency of power generation by the system was consistent throughout the 7 days of test (Figure 2g in the main text).

The results from both experiments showed that the system can consistently produce power with high efficiency after using it repeatedly for long durations of time.

S8. Obtaining Different Flow Patterns with the Same Flowrate

We investigated the relationship between the power generated and the pattern of flow of water down the tube. For investigating this relationship, we needed to obtain different types of flow patterns and determine the power generated by each flow pattern. Through varying the experimental setup, we obtained five different flow patterns (as shown in Figure 3a of the main text): the plug flow, plug-dripping flow, dripping flow, rivulet, and full flow in a tube. For a fair comparison among the different types of flow patterns, important parameters were kept the same for the different experimental setups. Specifically, each experiment involved 50 mL of deionized water that flowed at a rate of 80 mL/min. A syringe pump was used for regulating the flow of these controlled experiments. The syringe was fixed to a stainless-steel needle that had a sharp tip. The tip of the stainless-steel needle tapered off asymmetrically on one side; the longest side of the tapered tip was 4 mm longer than the opposite shortest side. It was connected electrically to ground at (P2). (A discussion of the effect of grounding the stainless-steel needle or not is included at the end of this section.) When operating the pump, the water was driven out of the

syringe, via the stainless-steel needle, and into a tube. Tubes made of fluorinated ethylene propylene (FEP) were used for all the experiments. The tube was oriented vertically. The water that flowed vertically down the tube by gravity was then collected by a stainless-steel cup placed at the bottom of the FEP tube. The cup was electrically insulated from the surroundings by covering its exterior with insulating materials. The cup was connected electrically to resistors at (P1) with a total resistance of 31 GΩ connected in series, and then to ground. An electrometer (Keithley, model 6514) was used to measure the potential difference across the 1 GΩ resistor among the series of resistors at (P1). The electrometer was connected to a computer that allowed data acquisition to be done automatically through a LabVIEW program. These aspects of the experimental setup mentioned above were the same for all the different types of flow patterns. The slight differences in the experimental setups for generating the different flow patterns are described as follows.

The experimental setup used for generating the plug flow was similar to that described in the Methods. The syringe needle fixed onto the syringe was oriented horizontally and the tapered opening at the tip of the needle faced downward. A FEP tube with a length of 32 cm and an inner diameter of 2 mm was used. Half of the top 3 cm of the tube was cut out vertically and was placed close to the metallic needle of the syringe. Specifically, the tip of the needle was 1 cm vertically below the top of the tube and 1 cm horizontally away from the exposed inner wall of the remaining half of the FEP tube that the water first came into contact. Whenever water was pumped out of the syringe and flowed down the FEP tube, power was generated (Table S2). The plug flow consisted of short discrete cylindrical columns of water that filled the whole diameter (i.e., 2 mm) of the tube interspersed with air in between (Figure 3a(I) in the main text and Movie S1).

The experimental setups for obtaining the plug-dripping flow and dripping flow were the same as described for obtaining the plug flow, except that the FEP tubes used had larger diameters. For the plug-dripping flow, a FEP tube with an inner diameter of 3 mm was used. For the dripping flow, a FEP tube with an inner diameter of 6 mm was used. Because the tubes used were larger, the water was not able to fill the whole inner circumference of the tube. Hence, the dripping flow only consisted of discrete and separated water drips flowing down a portion of the inner surface of the tube (Figure 3a(III) in the main text and Movie S3). The flow was random and swirling as it flowed downward. The plug-dripping flow was an intermediate case between the plug flow and the dripping flow due to the medium size of the tube (i.e., 3 mm). The plug-dripping flow was observed to consist of frequent repeated changes between the plug flow and the dripping flow (Figure 3a(II) in the main text and Movie S2).

By varying the experimental setup slightly, we were able to generate two more types of flow patterns: the rivulet and full flow. For generating these flow patterns, the metallic syringe needle connected to the syringe pointed vertically downward. For obtaining the rivulet, a FEP tube with a large inner diameter of 6 mm and a shorter length of 10 cm was used. A shorter length was used because it was the maximum length at which the rivulet could be obtained robustly; due to the Plateau–Rayleigh instability, the rivulet had a tendency to break up into discrete drips beyond this length. The shortest side of the tapered tip of the syringe needle was 1 mm vertically below the top of the tube and 1 mm horizontally away from the nearest inner wall of the tube that the water first came into contact. This rivulet was observed to be a wavy continuous column of water that flowed down a specific portion of the inner wall of the tube that had a large diameter (i.e., 6 mm) (Figure 3a(IV) in the main text and Movie S4).

Besides this rivulet, we obtained the power generated for all the other types of flow patterns by using the tube with a length of 32 cm. For comparing the rivulet with the dripping flow, we repeated the same experiment with the dripping flow except that we used a tube with a length of 10 cm as well (Table S2).

For obtaining the full flow, a FEP tube with a small inner diameter of 1 mm and length of 32 cm was used. The tip of the metallic syringe needle was inserted 2 cm into the small tube from the top downward. To ensure a tight fit between the syringe needle and the tube for generating the full flow, a gauge 19 syringe needle (i.e., outer diameter 1.067 mm and inner diameter 0.686 mm) was used. Hence, the outer surface of the needle was in tight contact with the inner wall of the tube. The full flow was observed to fill the whole tube completely without any air in the tube as the water flowed down the tube (Figure 3a(V) in the main text and Movie S5).

As a control experiment, the same procedure was repeated except that the tube was removed; thus, the drips of water that were ejected by the syringe simply fell down vertically through air and into the cup. The power generated from the cup at (P1) was measured to be negligible at ~10^{-4} μW (Table S2). Using the same setup, we also measured that the power generated from the needle at (P2) was similarly negligible at ~10^{-4} μW. The currents were both positive in these two measurements.

Comparing our results for the different flow patterns (Table S2), the power generated clearly followed the trend: plug flow > plug-dripping flow > dripping flow > rivulet > full flow > control without the tube. Importantly, the differences in the power generated among the different flow patterns were dramatically large and involved many orders of magnitude (see Figure 3b in the main text and Table S2). For the optimized plug flow, a power of 170 μW was obtained at

(P1). Therefore, the power generated by the plug flow was 5 orders of magnitude larger than that generated by the full flow and 6 orders of magnitude larger than that generated by the control without the tube.

One factor that affected the efficiency of power generation was the length of the tube. We used the tube with a length of 32 cm because this length gave us the optimal efficiency of power generation for the plug flow. Any length shorter or longer produced lower efficiency. Because we could only use the tube with a length of 10 cm for the rivulet (i.e., due to the Plateau–Rayleigh instability), we also used a tube with a length of 10 cm for the dripping flow for comparison. This comparison showed that the power and efficiency generated by the dripping flow are greater than that of the rivulet. On the other hand, the rivulet clearly had a larger power and efficiency generated than that of the full flow despite flowing down a shorter tube.

The trend observed in Table S2 was probably due to two main reasons. The first reason involved the area of contact between the water and solid (per unit volume of water). In particular, the discrete columns of water generated by the plug flow filled the whole inner circumference of the tube, whereas the discrete water drips generated by the dripping flow flowed down only one side of the tube. Therefore, the water generated by the plug flow had a larger area of contact with the solid surface per unit volume than the water generated by the dripping flow. This larger area of contact per unit volume of water possibly generated the larger amount of power. Because the plug-dripping flow switched intermittently between the plug flow and the dripping flow, the power generated was in between the two types of flows.

The second reason involved the discontinuity of the flow as discussed in the main text. In general, the continuous flow patterns (i.e., the rivulet and the full flow) had much lesser power generated than the discontinuous flow patterns. Another important point to note is that the rivulet

generated a greater amount of power than the full flow. This result suggested that the slight and random waviness of the edges of the rivulet allowed the power generated to be larger. This result indicated that any type of slight perturbations that produce any disruption to the continuity of the flow can cause large differences in the amount of power produced by the flow across the solid surface.

The metallic syringe needle used in the experiments was connected electrically to ground. The reason for connecting the needle to ground was because it gave a higher power and efficiency for the plug flow (i.e., the optimal type of flow for generating power in this study). To clarify this point, we determined the power generated by the plug flow when the needle was not grounded. In this case, we found that the power generated was 8 times lesser than when the needle was grounded (Figure S2a). An important point to note is that the grounding did not need to be at the metallic syringe needle. We tested and found that we could generate similar amounts of power if we changed the grounding at the metallic syringe needle to other positions, including around the water stream at the top of the FEP tube. We found that as long as the water was electrically grounded and the grounding wire did not interfere with the flow pattern, the power generated was similar. Subsequently, we compared the power generated by the full flow when the needle was grounded and not grounded. Results showed that the power generated by the full flow was larger when the needle was not grounded than when it was grounded. Both amounts of the power generated, however, were on the same order of magnitude — hence, the power was negligible regardless of whether the needle was grounded or not. In conclusion, the power generated by the optimal plug flow (i.e., with grounding) was larger than the full flow by approximately the same orders of magnitude regardless of whether the needle was grounded or not.

We further verified these results by flowing water across another type of solid surface: the PTFE V-shaped channel. We first determined the power generated by flowing the dripping flow down the V-shaped channel inclined at 30° to the vertical. We compared the power generated when the metallic syringe needle was grounded and not grounded. The result showed again that the power generated when the metallic syringe needle was grounded was larger than when it was not grounded (Figure S2b). We repeated the experiment by flowing a rivulet down the V-shaped channel. The result was again similar to the case when the FEP tubes were used. The power generated was larger when the needle was not grounded; however, both the amounts of power generated were on the same order of magnitude. In general, the difference between grounding and not grounding the needle was small relative to the difference between continuous and discontinuous flow patterns. Hence, the conclusions of this study remained the same regardless of whether the syringe needle was grounded or not.

S9. Dripping-Rivulet Flow Transition for Investigating the Effect of the Flow Pattern on the Power Generation

We showed that the amount of separation of charge was highly dependent on the pattern of the flow based on varying different types of flow patterns and measuring the power generated. Those experiments, however, involved various aspects of the setup that were changed for varying the pattern of flow, including the tube diameter and the orientation of the syringe needle. To establish an even clearer relationship between flow patterns and the amount of separation of charge, we performed an experiment in which only one minor parameter of the experiment was varied: the distance of separation between the tip of the syringe needle and the solid surface. This series of experiments is divided into three parts. In the Methods section, we described the

experiment that involved inducing the transition with a V-shaped PTFE channel. This section contains the other two parts. The first part describes the experiment that involved inducing the transition with a glass tube. The second part discusses the fundamental mechanism of the dripping-rivulet flow transition.

S9.1. Dripping-Rivulet Flow Transition using a Glass Tube

We further investigated the generality of the phenomenon by using a different solid material (i.e., instead of the V-shaped PTFE channel). In this experiment, we used a glass tube with an inner diameter of 4 mm and length of 20 cm. The dripping-rivulet flow transition was induced by changing the distance of separation between the tip of the needle and the inner surface of the glass tube onto which the water first landed.

The glass tube was first washed and discharged with ultrapure water and ethanol, and dried by blowing a stream of nitrogen gas across the surface. It was then tilted to an angle of 30° to the vertical and fixed in position. A syringe pump was used to regulate the flow of water down the glass tube for this controlled experiment. The tip of the needle of the syringe was oriented horizontally. It was placed above the top opening and within the circular top of the tube. Specifically, the tip was positioned at a horizontal distance of 1 mm away from the specific part of the inner wall of the glass tube that the water first came into contact. Water was ejected into the inner wall of the glass tube at a constant flowrate of 20 mL/min. For creating the dripping flow, the tip of the needle was placed at a height of 2 mm above the top opening of the glass tube. For creating the rivulet, the tip was placed at the same height as the top opening of the glass tube.

S9.2. Fundamental Mechanism of the Dripping-Rivulet Flow Transition

Transition from dripping flow to rivulet is usually achieved by major changes in the experiment; examples include a different flowrate of the liquid (i.e., a larger flowrate for obtaining a rivulet and smaller flowrate for obtaining a dripping flow) and a different solid surface (e.g., the different diameters of the tubes used as described in Figure 3a of the main text). However, major changes in the experiment may also affect the process of generation of electricity. Hence for a fair comparison, we induced the dripping-rivulet flow transition by simply varying d_{sep}, while keeping all the other parameters of the experiment — including the important parameters such as flowrates and the type of solid surface used — the same. We showed that this method for inducing the flow transition is general by using two types of solid surfaces (i.e., the V-shaped PTFE channel and the glass tube). The ability to induce the transition via this simple parameter d_{sep} without any other changes in the experimental setup was initially unexpected. Changing the parameter d_{sep} basically allows the amount of interaction between the water that is being pumped out of the syringe needle and the solid surface to be varied. Specifically, the smaller the d_{sep}, the more interaction between the water and the solid surface. Hence, the adhesion of water onto the surface may have allowed the water to spread and form a rivulet when d_{sep} is smaller.

To understand the fundamental mechanism of the flow transition, we performed a set of conceptually simpler experiments as shown in Figure S3. The experiment involved a needle (tip diameter ~0.6 mm) with a flat tip that pointed vertically downward. A syringe pump was used to regulate the flow of water out of the needle carefully at a low flowrate of 0.05 mL/min. Without any solid surface nearby (i.e., similar to the case when d_{sep} is large), the process was similar to that of a "pendant droplet": as the liquid was pumped out at a slow flowrate, the drip hung onto

the end of the tip of the needle due to surface tension. As the mass of the drip increased due to the gradual flow of water out of the needle, a sufficient amount of mass of the liquid, m_{crit}, was reached when the drip detached from the needle and fell under its own weight. This critical mass, m_{crit}, is determined by the balance of the force due to surface tension around the tip of the needle and the weight of the drip. Through balancing the forces, the critical mass can be expressed as $m_{crit} = 2\pi R\gamma/g$, where R is the diameter of the tube, γ is the surface tension of the liquid, and g is the gravitational acceleration. Therefore, the water that flowed out of the needle did not immediately flow away from the tip as a rivulet. The process allowed the water to accumulate its mass as a drip at the tip and then fall off as a discrete drip. When we used the V-shaped PTFE channel for performing the dripping-rivulet flow transition, we did observe that the water flowed out of the needle as discrete drips when d_{sep} was large. They remained as discrete drips as they flowed down the valley of the channel. Similarly, discrete drips were observed for this conceptually simpler experiment when water flowed out of a needle that was pointing vertically downward. For determining the m_{crit}, videos of the flow of the water out of the needle were taken by a Nikon D5300 camera equipped with a Nikon AF Micro-NIKKOR 200mm f/4D IF-ED lens. The frame rate was 50 frames per second. The sizes of the drips that fell off the tip of the needle were determined by multiplying the number of frames between two falling drips and the flowrate of the water per frame (1.67×10^{-5} mL/frame).

To investigate the flow when a solid surface was involved, we used three types of surfaces with very different wettability: glass, PTFE, and glass coated with a layer of superhydrophobic coating (Neverwet®). The contact angles of water on the (horizontal) surfaces were determined to be about 30°, 100°, and 150° for glass, PTFE, and glass coated with the superhydrophobic coating respectively (Figure S3). For this experiment, one of the surfaces was

oriented vertically and placed close to the tip of the needle as shown in Figure S3. This setup was thus similar to the case when the d_{sep} between the V-shaped PTFE channel and the needle was small for inducing the rivulet. When water was pumped out of the tip of the needle, we observed that the water interacted with the solid surface placed close to it. When more water was pumped out of the needle, the drip of water detached and fell off the tip of the needle under its own weight. The experimental images presented in Figure S3 showed the moment just before the water drip fell off the tip of the needle when they interacted with different types of surfaces. Two observations can be made from this set of experimental images. First, the water spread more onto the surfaces that were less hydrophobic. Second, the set of images presented in Figure S3 showed that the volume of water was in the following order: water on glass < water on PTFE < water on surface with superhydrophobic coating < water not in contact with any surface. Hence, a smaller volume of water detached from the tip of the needle to the surfaces that were less hydrophobic. These two observations suggested that the tendency of water to spread onto solid surfaces allows the water drips to detach easier — with less volume — from the tip of the needle than when the water is not in contact with any surface. This phenomenon is the mechanism that underlies the dripping-rivulet flow transition when the water flowed down the V-shaped PTFE channel with varying d_{sep}. When d_{sep} is smaller, the water comes into contact with the surface; hence, the water spread out more and detached faster from the tip of the needle. Therefore, the water forms a rivulet on the surface more readily.

S10. Flow with Discontinuity Charges Against a Surface Only Positively

Our results showed that the amount of power generated by the plug flow was five orders of magnitude more than that generated by the full flow. Besides the amount of power generated, we

investigated the polarity of charging of the two different types of flow to understand the fundamental mechanism better. For this investigation, we coated surfaces with either polycations or polyanions and flowed water (i.e., either continuous flow or flow with discontinuity) across the surfaces. This section is divided into three parts. The first part describes the preparation of the surfaces coated with the polyelectrolytes. The second part describes the characterization of the surfaces after coating. The third part describes the experiments for determining the polarity of charge of water that flowed either continuously or discontinuously across the coated surfaces.

S10.1. Preparation of the Surfaces Coated with Polyelectrolytes

Four types of polyelectrolytes were used. They were poly(ethyleneimine) (PEI), poly(allylamine hydrochloride) (PAH), poly(diallyldimethylammonium chloride) (PDDA), and poly(sodium 4-styrenesulfonate) (PSS). Each of these polyelectrolytes was coated on the surface of glass using an established procedure commonly reported in previous studies.[15-19] First, glass coverslips were cleaned by washing them with ethanol and ultrapure water, and drying them in an oven for 1 h. They were then further cleaned with the piranha solution. The piranha solution (28 mL) consisted of 21 mL sulfuric acid (98% w/v) and 7 mL hydrogen peroxide (35% w/v). For treating the coverslips with the piranha solution, the glass coverslips were first placed into an empty beaker. The solution that contained only the hydrogen peroxide was poured into the beaker. The sulfuric acid was then poured slowly into the beaker. The glass coverslips were left in the solution for 30 min. (*Attention: The piranha solution is dangerous and should be handled with care.*) After the treatment, the glass coverslips were then washed with ultrapure water and dried by a stream of nitrogen gas.

An aqueous solution that consisted of one of the polyelectrolytes (e.g., PEI, PDDA, PAH, or PSS at 5 wt%) and deionized water was prepared. The aqueous solution was adjusted to pH 4 by adding hydrogen chloride (HCl) to the solution. The cleaned pieces of glass coverslips were placed in the aqueous solution containing the polyelectrolytes for 3 h. After coating the coverslips with the polyelectrolyte, they were washed with ultrapure water and dried by a stream of nitrogen gas. They were further dried in an oven at 80 °C for 1 h.

S10.2. Characterization of the Surfaces Coated with Polyelectrolytes

We verified that the surfaces of glass were successfully coated with the polyelectrolytes via three types of analyses: confocal microscopy, X-ray photoelectron spectroscopy (XPS), and measurement of contact angle. Each of these analyses will be discussed sequentially in the following paragraphs.

For verifying if the polyelectrolytes were successfully coated onto the surface by confocal microscopy, a fluorescent marker, FITC, first needed to be deposited onto the surface. The FITC solution was prepared by mixing 10 mg of FITC in 100 mL of DMSO. To label the surface with FITC, we coated the surface of glass with either PEI or PAH using the procedure described in the above section. These coated surfaces were then immersed in solutions containing the FITC for 12 h. After reacting with FITC, the surfaces were washed with DMSO and ethanol, and dried by blowing the surfaces with a stream of nitrogen. We observed the surfaces using confocal microscopy after labeling them with FITC. We observed the surfaces using confocal microscopy after labeling them with FITC (Figure 4c(iii, v)). We found that the glass surfaces were homogeneously coated with the FITC attached to the polyelectrolytes. In addition, we performed the experiment in which we flowed water across the glass surfaces for 5

min and observed the surfaces using confocal microscopy again. Similarly, the surfaces appeared homogeneously coated with the FITC attached to the polyelectrolytes (Figure 4c(iv, vi)). Hence, the flow of water did not disrupt the coating of polyelectrolytes on the surface of glass.

As a control experiment, we repeated the procedure for a piece of glass that was not coated with the polyelectrolytes. Specifically, the bare piece of glass was first washed in the same way as the piece of glass coated with the polyelectrolytes by ethanol, ultrapure water, and piranha solution. After washing, the surface of glass was deposited with FITC using the same method described above. The surface was then washed similarly with DMSO and ethanol, and dried with nitrogen. In this case, however, we were not able to observe the FITC on the surface of glass without the layer of polyelectrolytes coated on the surface by confocal microscopy (Figure 4c(i)). We were also not able to observe anything on the surface of glass after flowing water across its surface for 5 min (Figure 4c(ii)). Therefore, these results indicated that the surface of glass needed to be coated with both the polyelectrolyte and FITC for the fluorescent signal to be observable by confocal microscopy. Hence, the observation of the fluorescent signal indicated that the surfaces of glass were coated with polyelectrolytes.

The chemical compositions of the surfaces coated with the polyelectrolytes were analyzed using X-ray photoelectron spectroscopy (XPS). XPS spectra were recorded on a PHI-5000C ESCA system (Perkin-Elmer, USA) with Al Kα excitation radiation (1486.6 eV). The pressure in the analysis chamber was maintained at 10^{-6} Pa during the measurement. All spectra were referenced to the C 1s hydrocarbon peak at 285.0 eV to compensate for the effect of surface charging.

Table S3 and S4 show the chemical compositions of five types of samples analyzed by XPS. The first rows of Table S3 and S4 show the analyses of pieces of bare glass not coated with

the polyelectrolytes. The subsequent rows show the analyses of glass coated with the respective polyelectrolytes as indicated. For a fair comparison, the pieces of bare glass were subjected to the same process of cleaning as the coated glass. Specifically, the pieces of glass were washed with ethanol and ultrapure water, dried in an oven for 1 h, and then treated with the piranha solution. After cleaning, the surfaces were analyzed (i.e., the results shown in the first rows of Table S3 and S4). For analyzing the coated surfaces, the surface of glass was cleaned in the same way and coated with the respective polyelectrolytes as indicated in the tables. The analyses of the surfaces of glass coated with PEI, PDDA, PAH showed larger amounts of nitrogen compared to the surface of the bare uncoated glass (Table S3). The analysis of the surface of glass coated with PSS showed a larger amount of sulfur compared to the surface of the bare uncoated glass (Table S4). These results thus showed that the surfaces were successfully coated with the respective polyelectrolytes.

The contact angles of water on surfaces coated with the different types of polyelectrolytes (i.e., PEI, PDDA, PAH, and PSS) were measured (Table S5). For comparison, the contact angle of water on the bare uncoated surface of glass was also measured. This surface of the bare glass was washed with ethanol and ultrapure water, dried in an oven for 1 h, and then cleaned with piranha solution before the analysis (i.e., the same treatment as the surfaces coated with the polyelectrolytes before the measurement). The significant differences in the contact angles between the uncoated and coated surfaces suggested that the surfaces were coated.

S10.3. Determining the Polarity of Charge

An electrokinetic analyzer (SurPASS, Anton Paar, Graz, Austria) was used to determine the polarity of the water after charging the water against surfaces coated with polyelectrolytes by a

continuous stream. The standard operation of the analyzer involved the following procedure. Two flat pieces of glass (each with dimensions of 20 mm × 10 mm and thickness of 0.13–0.16 mm) coated or uncoated with polyelectrolytes were placed vertically in parallel in the cell in the analyzer. The cell had a feature that allowed the horizontal distance of separation between the two flat pieces of glass to be adjusted between 100–150 μm. This gap created by the separation of the two pieces of glass formed the micro-channel through which the solution flowed. Each solution (with or without 0.01 M NaCl) of a specific pH was then pumped through this gap continuously using a syringe pump. 0.01 M NaCl solutions (adjusted to the desired pH) were used according to the ISO and ASTM standards;[20, 21] at the same time, we also used deionized water without the NaCl (adjusted to the desired pH) for our investigations. The solutions were made acidic by adding hydrochloric acid or basic by adding sodium hydroxide. Electrodes were placed at the inlet and outlet of the micro-channel for measuring the streaming current. After measuring the streaming current, the zeta potential of the surface of the solid substrate was calculated using the Helmholtz-Smoluchowski equation.

Results from the analyzer by flowing a continuous stream of deionized water (i.e., without any adjustment in pH) across the surfaces of glass coated with PEI, PDDA, and PAH showed that the zeta potentials of all these surfaces were positive (Figure 4d in the main text). On the other hand, the zeta potential measured by flowing a continuous stream of deionized water across the surface of glass coated with PSS was negative. These results are expected because the PDDA, PAH, and PEI are polycations, whereas PSS is a polyanion at neutral pH. The measurement of the zeta potential of the solid surfaces was repeated for aqueous solutions of different pH that ranged from pH 2 to pH 12 (Figure 4d in the main text). Results showed that the zeta potentials were positive for a wide range of conditions, including pH of the solutions

that ranged from ~2 to ~8 for surfaces of glass coated with either PEI, PDDA, or PAH. The zeta potential measured by the analyzer is based on driving the mobile counterions close to the solid surface by the continuous flow of water toward the outlet of the channel. Because the measurement involved the quantification of the current of the flow of the mobile counterions, the polarity of the aqueous solution at the outlet of the flow is opposite to the zeta potential of the solid surfaces (e.g., as illustrated in Figure 4a in the main text). Therefore, the polarity of the continuous flow of water at the outlet of the channel was *negative* for the wide range of conditions that produced positive zeta potential (i.e., the flowing of solutions with pH from ~2 to ~8 across surfaces of glass coated with either PEI, PDDA, or PAH). Solutions with or without the NaCl showed similar results.

We then investigated the flow with discontinuity across the solid surfaces. All the solid surfaces (5.0 cm × 2.4 cm × 0.13 mm) coated with the same types of polyelectrolytes were first washed with ultrapure water, dried with nitrogen gas, and discharged using an antistatic (Zerostat) gun. 20 drips (30 µL each) of aqueous solution were allowed to slide at a flowrate of 1 mL/min down the solid surface that was tilted at an angle of 20° to the vertical over a distance of 5 cm. After they fell off the surface, they were collected in the Faraday cup for measuring their charges by the electrometer. Since the solid surface was 2 cm above the Faraday cup, the total vertical height that the liquid fell was ~7 cm. The experiment was performed for aqueous solution of different pH and different types of solid surfaces, including uncoated glass or glass coated with either PEI, PDDA, PAH, or PSS. In addition, we performed a control experiment in which we simply dropped the drips from the same height of ~7 cm directly into the Faraday cup without allowing them to come into contact with any solid surface. All the results based on using these different conditions surprisingly showed that the discontinuous liquid charged only

positively (Figure 4e in the main text). These results included the charging of the liquid flow with discontinuity against all types of solid surfaces uncoated or coated with the different types of polyelectrolytes for all the pH investigated. In all cases, the drips that contacted the surfaces charged more positively than without contacting any surface (i.e., the control experiment). On the other hand, we discussed that the charging of the continuous stream of water across the surfaces coated with the polyelectrolytes generated negatively charged liquid under a wide range of conditions. Therefore, these results indicated definitely that the charging of flow with discontinuity is fundamentally different from charging by the continuous flow.

S11. Molecular Species for Generating Positively Charged Water by Flow with Discontinuity

Our experimental results (as discussed in Section S10) showed that the flow with discontinuity charged water positively against solids regardless of the type of surface (e.g., surfaces that were coated with either polycations or polyanions). In this section, we describe our experiments for investigating the molecular species that generates the positively charged water by the flow with discontinuity. We performed two types of chemical analyses of the water before and after flowing the water across a surface: we determined the change in pH of the liquid and analyzed the chemical composition of the liquid by NMR.

We investigated the change in pH of the liquid because of the following reasons. First, the result that water charged only positively against different types of surfaces suggested that the charge species separated in the liquid is not due to the surface — but due to the water itself. Second, previous studies have reported (i.e., including results from both experiments and simulation) that there is preferential adsorption of OH^- ions from the water onto the surfaces at

the solid-liquid interface. To verify if the preferential adsorption of OH^- ions onto surfaces is the molecular mechanism for generating the positively charged water, we measured the change in pH of the liquid before and after flowing it across a solid surface.

Specifically, the experiment involved flowing the liquid down a V-shaped PTFE channel. The channel had a length of 33 cm. Each of the two parts of the V-shape was 2.5 cm wide. To investigate the adsorption of OH^- ions, we used a basic aqueous solution at pH 9; the solution consisted of only deionized water and NaOH. 25 mL of the basic aqueous solution was first placed in a 40 mL glass vial. The initial pH of the solution was measured. It was then transferred into a 50 mL syringe. The syringe was connected to a metallic syringe needle that was electrically grounded for first grounding the liquid. The syringe needle was then removed and the basic aqueous solution was pumped directly (i.e., via a syringe pump) onto the V-shaped channel. We used a slow flowrate of 5 mL/min for creating the flow with discontinuity. After flowing down the V-shaped channel, the solution was collected by a 40 mL empty glass vial. The final pH of the solution in the glass vial was measured after collecting all the 25 mL of the solution in the vial. We then determined the change in pH of the solution by taking the difference of this final pH after flowing the solution down the channel and the initial pH of the solution before flowing the solution down the channel (i.e., labeled "Discontinuous" in Figure 5b in the main text). We repeated the experiment described above except that we used a high flowrate of 60 mL/min for creating the continuous flow (i.e., labeled "Continuous" in Figure 5b in the main text). For a separate control experiment, we flowed the same volume (i.e., 25 mL) of the basic aqueous solutions out of the syringe and directly into the 40 mL empty glass vial without any contact with the solid surface and measured the difference between the initial pH and the final pH after pumping the solution out and collecting it in the glass vial (i.e., labeled "Control" in

Figure 5b in the main text). All experiments were performed under an inert (i.e., nitrogen) atmosphere to eliminate any influence of the surrounding atmosphere on the pH of the solution. Our results showed that there was a significant decrease in pH (i.e., a reduction in pH of ~0.27) of the solution when it flowed down the channel discontinuously (Figure 5b in the main text). On the other hand, the results obtained from the continuous flow of the solution and the control experiment did not show any decrease in pH.

We analyzed the chemical composition of the deionized water that was either discharged or positively charged. The positively charged water was obtained by flowing uncharged water through a discharged FEP tube. NMR spectra were recorded on a Bruker Avance 400 MHz NMR Spectrometer (DRX400). The results of the analyses showed that there was no difference in the chemical compositions of the discharged water and the positively charged water as analyzed by NMR (Figure 5c).

In conclusion, the results of all these experiments suggested that the molecular species of the positive charge in water separated by the flow of water with discontinuity across a solid surface is H^+ ions. When water molecules come into contact with a solid surface, previous studies reported that there is preferential adsorption of OH^- ions over H^+ ions on the solid surface. When the finite volume of water moves away from the surface (i.e., because the flow is discontinuous), the OH^- ions are left behind — hence, there is an excess amount of H^+ ions left in the water. This excess amount of H^+ ions may be the molecular species of the positively charged water regardless of the type of surface that it flows across. By not detecting any differences in the chemical composition by NMR, this result further suggested that the positive charge is not due to other types of molecular species; instead, the species is present in the water itself (i.e., the H^+ ions).

S12. Location of Charging is at the Trailing Edge of the Flow with Discontinuity

Our experiments showed that the charging of the flow of water with discontinuity across a surface was fundamentally different from the charging of continuous flow of water across the surface — flow with discontinuity produced only positively charged water and an amount of charge that was far larger than that of the continuous flow. To understand the fundamental mechanism, we determined the location of charging in the water as the flow of water with discontinuity moved through the tube. The typical experiment for obtaining the large amount of charging involved the plug flow in which short discrete columns of water were separated by pockets of air. However, it is challenging to determine the location of charging if the discrete columns of water were short. Hence, our investigation for determining the location of charging involved a long column of water. Because a longer column of water was needed, we used a longer tube for this investigation. Details of the experiment are as follows.

A syringe pump was used for carefully regulating the flow of water through the tube. We used a long FEP tube with an inner diameter of 2 mm (i.e., the tube that provided the highest efficiency of power generation) and a total length of 70 cm. We made a connector (length: 3 cm) using the same FEP polymer for connecting the outlet of the plastic syringe mounted on the pump to the FEP tube. The connector was wrapped with layers of parafilm to prevent any leakage. The long FEP tube was bent gently (i.e., the angles of bending were gradual for allowing smooth flow of water across the bent portions) twice. Hence, the tube consisted of three sections: an initial vertical section of 7.5 cm, a horizontal section of 30 cm, and a final vertical section of 32.5 cm. The final vertical section was intended to mimic the typical experimental setup for producing the highest efficiency using a vertical FEP tube (2 mm) that had a length of 32 cm. A Faraday cup connected to an electrometer was placed below the outlet of the tube for

measuring the charge of the water as it flowed out of the tube. A 100 mL glass beaker was placed inside the Faraday cup for collecting the water that flowed out of the tube. The Faraday cup was placed far away from the outlet of the tube (~30 cm below the outlet) to prevent the charge of the tube from influencing the measurement of the charge of the water in the Faraday cup.

The experiment involved pumping 43 mL of pre-discharged deionized water at a flowrate of 125 mL/min through the tube. The whole setup was electrically insulated (i.e., including the insulating FEP connector that was not grounded). Immediately following the flow of 43 mL of water, 7 mL of air was pumped through the tube. The pumping of air immediately after flowing the 43 mL of water allowed all the water to be driven out of the tube. In general, this operation allowed a single discrete column of water to flow through and leave the tube as a whole into the Faraday cup. The column of water was continuous and filled the whole inner circumference of the tube completely (i.e., no air pockets within the column of water). The electrometer was able to measure the charge within the Faraday cup rapidly at a rate of 40 times per second. Therefore, this setup was able to determine the gradual accumulation of charge in the Faraday cup in real time as the water gradually accumulated in the Faraday cup. The electrometer was operated throughout the whole process; hence, charge was measured from the beginning when the water first flowed into the Faraday cup to the end when all the water flowed out of the tube. The experiment was performed in triplicate.

We found from our measurements that the charge of the whole column of water was mainly located at the last 3 mL of the water at the trailing edge and was negligible in the first 40 mL. To show this result, we plotted the charge per unit volume of five different portions of the discrete column of water: (1) the first 10 mL, (2) 10 mL – 20 mL, (3) 20 mL – 30 mL, (4) 30 mL – 40 mL, and (5) the final 3 mL of the column of water (Figure 5g in the main text). The

final 3 mL had a different volume than the other four portions. Hence, for a fair comparison among the different portions of water of different volumes, we plotted our measurements in terms of charge per unit volume of each portion of water. Our results clearly showed that the charge was located mainly at the final 3 mL of the water — the charge was mainly concentrated around the trailing edge of the water column.

We note that in a typical experiment in this study, we connected the stainless-steel needle fixed to the syringe electrically to ground. We described in Section S8 that grounding the needle while the water flowed out of the syringe pump allowed the charge separation to be enhanced. Hence, as a control experiment, we repeated the experiment described above except that we grounded the FEP connector. For this experiment, we inserted an additional piece of aluminum foil halfway into the inner wall of the connector; thus, half of the piece of aluminum foil was exposed to the outside. The portion of the aluminum foil exposed to the outside was connected electrically to ground. The connector was then wrapped with extra layers of parafilm again after connecting to the ground to prevent any leakage caused by the addition of the aluminum foil. We similarly pumped 43 mL of pre-discharged deionized water followed by 7 mL of air at a flowrate of 125 mL/min through the tube. Our results showed the same trend (Figure S4): the charge was mainly located in the last 3 mL of the water.

A plausible explanation why the charge was located only in the last 3 mL of the discrete column of water is that the separation of charge may be occurring right at the very trailing edge of the water column — charge separation occurs only at the receding line of contact between the liquid and solid as the water retracts from the solid surface at the trailing edge. When the charged ions of the same polarity (i.e., positive) are separated at the receding line of contact, the repulsive electrostatic forces drive the ions away from one another; hence, the ions migrate rapidly away

from the receding line of contact and disperse into the bulk volume of the water. This dispersion of ions probably allows the charge to be measured in the last 3 mL of the bulk volume of the water around the trailing edge (i.e., not just at the very end of the trailing edge). In any case, it can be concluded that substantial charge separation does not occur at the leading edge or in the middle section of the column of water due to the lack of charge measured in the first 40 mL of the discrete column of water.

One experimental observation supported the mechanism that charge separation happened only right at the trailing edge: the charge was detected by the electrometer only when the trailing edge appeared visually in the tube. For the purpose of this discussion, we first quantify the volume of the system that needed to be filled with water for this experiment performed for locating the charge in the water column. When water flowed, it filled (i) into the tube and (ii) the space between the bottom outlet of the tube and the Faraday cup (i.e., before the charge of water is measured). The tube had a diameter of 2 mm and a total length of 70 cm. The space between the bottom outlet of the tube and the Faraday cup was around 30 cm. The diameter of the column of water as it flowed out of the tube was approximately the same as in the tube (i.e., 2 mm). Hence, the total volume that water needed to fill into this system was ~3 mL. On the other hand, we flowed a total volume of 43 mL of water through the system in this experiment; hence, the total volume flowed was far larger than the volume of the water filled in the system (i.e., the column of water that flowed through the tube and into the Faraday cup). Because more water was used, the trailing edge did not appear initially in the experiment.

When the experiment first started, the pump started to drive water into the FEP tube. This initial flow of water into the tube allowed us to observe the leading edge of the column of water. As the water continued to be pumped out, we observed that the leading edge moved gradually

across and out of the tube. The leading edge vanished after the water entered the Faraday cup. After observing that the leading edge vanished, we observed a continuous stream of water flowing through the tube and into the Faraday cup. This continuous stream of water that fully filled the tube was observed for some time. In this process, negligible amounts of charge were measured as mentioned. After flowing all the 43 mL of water out of the syringe, we then pumped air to drive the water completely out of the tube. At this moment, the tube and the space between the outlet of the tube and the Faraday cup was fully filled with 3 mL of water. At the same time, we observed the trailing edge of the column of water at the beginning end of the FEP tube. As soon as we observed the appearance of the trailing edge of the column of water at the beginning of the FEP tube, we observed that the measurement of charge increased rapidly. Therefore, the creation of the trailing edge directly caused the increase in charge to occur — the substantial amount of charge generated was because of the trailing edge. Once the trailing edge appeared at the beginning of the FEP tube, charge separation occurred. The repulsive electrostatic force then allowed the ions to migrate rapidly through the tube and into the Faraday cup for the measurement of the rapid increase in charge. The rapid increase in charge was observed to continue until all the last 3 mL of water flowed out of the system and into the Faraday cup. This experimental observation thus indicated that the charging occurred right at the trailing edge.

To investigate whether the results were general, we repeated the experiment with a tube of a different size. For this experiment, we used a FEP tube with an inner diameter of 1 mm and a total length of 30 cm. To control the flow of water through the tube precisely, we ensured that there was a tight fit between the syringe needle and the tube. The tight fit was obtained using the same setup that was used for obtaining the full flow (Section S8). The setup involved a gauge 19 syringe needle (i.e., outer diameter 1.067 mm and inner diameter 0.686 mm) that was fitted

inside the 1 mm FEP tube. The Faraday cup connected to an electrometer was placed 20 cm below the outlet of the tube for measuring the charge of the water as it flowed out of the tube. A 50 mL glass beaker was placed inside the Faraday cup for collecting the water that flowed out of the tube. 3 mL of water followed by 1 mL of air at a flowrate of 25 mL/min was pumped through the tube. The whole setup was electrically insulated. The experiment was performed in triplicate.

We found from our measurements that the charge of the whole column of water was mainly located at the last 0.375 mL of the water and was negligible in the first 2.625 mL. To show this result, we plotted the charge per unit volume of four different portions of the discrete column of water: (1) the first 0.875 mL, (2) 0.875 mL – 1.75 mL, (3) 1.75 mL – 2.625 mL, and (4) the final 0.375 mL of the column of water (Figure S5). We quantified the volume of water in the system. The tube had a diameter of 1 mm and a total length of 30 cm. The space between the bottom outlet of the tube and the Faraday cup was around 20 cm. The diameter of the column of water as it flowed out of the tube was approximately the same as in the tube (i.e., 1 mm). Hence, the total volume that water needed to fill into this system was ~0.375 mL — this volume of the system again corresponded to the volume of water that the charge was located. This analysis further verifies that the charge separation happened only right at the trailing edge.

S13. Surface Conduction of OH^- Ions Up the FEP Tube

In the main text, we proposed a mechanism in which there is charge separation at the receding edge of the plug of water; the separated H^+ ions follow the water down the tube, whereas the OH^- ions migrate up the surface of the inner wall of the tube. We measured a continuous negative current at the top of the tube (P2) with a power generated that is almost similar to the power generated by the positive current at the bottom of the tube at (P1). We also determined that the

tube was negatively charged after the plug flow stopped and the tube did not contain any water in it. These results indicated that the negatively charged ions were generated on the surface of the tube and then migrated upward to the top of the tube.

In general, migration of ions on dry hydrophobic surfaces is difficult and slow. On the other hand, our system involves an extremely high amount of moisture due to the constant flow of water through the tube. Previous studies have determined that water molecules from a humid environment can adsorb onto hydrophobic surfaces such as PTFE and FEP.[22, 23] Previous studies have also determined that this adsorption of water molecules onto hydrophobic surfaces can increase their surface conductivities.[24-26]

We showed experimentally that the surface conductivity of FEP greatly reduced in a humid environment, thus facilitating the migration of ions on the surface. To show this point experimentally, we measured the surface resistivity of FEP surfaces using a standard Resistivity Test Fixture (Keithley Model 8009) connected to an electrometer (Keithley Model 6517B). To simulate the humid environment, we placed FEP sheets 15 cm on top of the outlet of a commercial humidifier and operated the humidifier for 30 s. The surface conductivity of the moist FEP sheet was then measured immediately. The analysis showed that the surface resistivity of the moist FEP sheet was on the order of 10^9 Ω/sq. The unit Ω/sq refers to the surface resistance of a surface with an aspect ratio of one (i.e., the same length and width). The surface resistivity of the original piece of FEP sheet before moisturizing it with the humidifier was on the order of 10^{16} Ω/sq. Therefore, the surface resistivity decreased by 7 orders of magnitude when the surface of FEP was moist.

Based on the measured surface resistivity, we calculated the overall surface resistance of the FEP tube. The conversion was performed by multiplying the surface resistivity with the

length of the surface (i.e., the length of the FEP tube, 32 cm) and dividing by the width of the surface (i.e., the perimeter of the circle, 6.28 mm). Therefore, the inner wall of the FEP tube has an overall surface resistance of around 100 GΩ. This amount of resistance is on the same order of magnitude as the resistive load used in our system (e.g., the typical 60 GΩ or 40 GΩ). Therefore, it is possible for OH^- ions to migrate across the inner surface of the FEP tube.

The analysis described above is for the case when the surface is exposed to air. On the other hand, when water flows down the tube as plug flows, the inner surface of the tube is exposed alternately to air and water. Water conducts ions readily. For a demonstration, we used an FEP tube of 32 cm (i.e., the tube that we use typically in our experiments) and filled it fully with water. We then measured the bulk resistance of the tube filled with water by attaching an electrode on each end of the tube. The resistance was measured to be about 0.1 GΩ. This resistance is much smaller than the resistive loads used in our typical setup; hence, ions can migrate much faster whenever water is on the surface of the FEP tube.

S14. Dissipation of Negative Ions into the Atmosphere

The experimental setup that generated the optimal amount of power and efficiency involved the plug flow of water down the FEP tube. We measured that the water that flowed out of the tube charged highly positively. When we connected the cup at (P1) that collected the positively charged water electrically, we obtained a positive electric current. At the same time, when we connected the metallic needle of the syringe at (P2) electrically, we obtained a negative electric current. The amount of positive current, however, was substantially larger than the negative current. By the law of conservation of charge, the separation of charge at the solid-liquid interface should produce an equal amount of positive charge and negative charge. Hence, the imbalance between the positive and negative charge indicated that an amount of negative charge

(i.e., equivalent to the difference between the measured positive current and the negative current) must have traveled elsewhere. There are two possibilities for accounting for the difference in negative charge: the accumulation of the negative charge on the surface of the solid and the dissipation of the negative ions into the atmosphere. We will discuss these two possibilities in the subsequent two sections respectively as follows.

S14.1. Solid Surface Did Not Charge Highly Negatively

In this section, we examined the possibility that the difference in the positive current and negative current was due to the continuous accumulation of the negative charge on the surface of the solid with time. We performed an experiment that involved a V-shaped PTFE channel of 5 cm long and 1 mm thick for investigating if the negative charge accumulated on the surface after contacting with the discontinuous flow. Each of the two sides of the V-shaped channel was 1 cm. The channel was first cleaned and discharged by rinsing it with ultrapure water and ethanol and drying it via blowing a stream of nitrogen gas over the surface. The channel was fixed in position at an angle of 30° to the vertical. A syringe pump was used to regulate the flow of water down the channel. A 50 mL glass syringe was mounted onto the pump. It was fitted with a stainless-steel needle (length: 15 cm and inner diameter: 0.838 mm) and was filled with deionized water. The stainless-steel needle had a sharp tip that tapered off asymmetrically on one side; the longest side of the tapered tip was 4 mm longer than the opposite shortest side. The needle was oriented horizontally and the opening of the tip faced upward. The needle was connected electrically to ground. The tip of the needle was placed 2 mm horizontally away from the innermost surface of the valley of the V-shaped channel to obtain the discontinuous flow. We used the pump to drive the water out of the needle at a constant flowrate of 15 mL/min. The water flowed a distance of

4.5 cm down the PTFE surface by gravity. After falling off the surface at the end, the water was collected in the Faraday cup for measuring their charges by the electrometer (Keithley, model 6514). The electrometer was connected to a computer that allowed the automatic acquisition of data via a LabVIEW program. The electrometer thus measured the accumulated charge of all the water that was collected in the cup. The measurement of charge was made for every 5 mL of water that flowed into the cup. At the same time that the measurement of charge was made for the charged water, the charge of the PTFE channel was also measured by another set of Faraday cup connected to another electrometer (Keithley, model 6514). Therefore, this experimental procedure allowed the charge of the accumulated water in the cup and the charge of the solid to be measured at the same time.

Our results showed that the flow of water with discontinuity was always charged positively to similar amounts throughout the whole experiment; hence, the total amount of charge measured in the Faraday cup increased approximately linearly with the volume of the accumulated water (Figure S6). After flowing 30 mL of water across the solid surface, the total charge of the water accumulated to a positive amount of around +20 nC. On the other hand, the piece of PTFE did not charge significantly even though it came into contact with the discontinuous flow that charged highly positively. After coming into contact with a small volume of water, the charge of the PTFE quickly saturated at a negative charge of around -1 nC (Figure S6). Further contact with the water did not charge the PTFE more although the discontinuous water flow continued to gain positive charge. These results showed that the solid surface was not able to accumulate negative charge — the large imbalance of positive charge and negative charge was not due to the accumulation of negative charge on the solid surface.

S14.2. Detecting the Negative Ions in the Atmosphere

We detected negative ions in the atmosphere surrounding the discontinuous water flow across the surface of a solid. In this experiment, we used a V-shaped PTFE channel that was 50 cm in length and 5 mm thick. Each of the two parts of the V-shape was 2.5 cm wide. It was first discharged by washing it with ultrapure water and ethanol thoroughly; it was then dried by a stream of nitrogen gas. The solid channel was verified to be discharged (i.e., < 0.1 pC/cm^2) before the experiment by measuring its charge with a Faraday cup connected to an electrometer (model 6514, Keithley). The PTFE channel was oriented almost vertically (i.e., slightly tilted at an angle of 5° to the vertical) and fixed in position. A syringe pump was used to drive 15 mL of deionized water at a flowrate of 15 mL/min onto the innermost surface of the valley of the V-shaped PTFE channel for 1 min. The water flowed a distance of 45 cm down the PTFE channel (Figure S7). We investigated two types of flows: the dripping flow and the rivulet. The method for obtaining these flow patterns was the same as described in Section S8. For obtaining the dripping flow, the horizontal distance of separation, d_{sep}, between the tip of the needle fixed onto the syringe and the innermost surface of the valley was 2 mm. For obtaining the rivulet, d_{sep} was 0.5 mm. For detecting the ions in the atmosphere, a Faraday cup with an inner cup diameter of 6.35 cm (2.5 inch) was used. By orienting the opening of the Faraday cup horizontally and bringing it close to the flow of water on the V-shaped channel, it would detect the ions in the atmosphere produced by the flow. This method of detection, however, is not effective because the Faraday cup would measure not only the ions in the atmosphere but also the charge of the solid surface and the liquid water. Hence, an alternative method of detection was needed for accurately sensing the ions in the atmosphere.

For avoiding the detection of the charge of the solid surface, we performed the sequence of experiments as follows. We first placed the V-shaped PTFE channel far away from the Faraday cup (i.e., with its opening oriented horizontally); at this state, we allowed the Faraday cup connected to an electrometer to measure the charge of only the air surrounding the cup for 30 s (i.e., the "initial state"). The amount of charge that we measured was negligible; hence, the charge from the background atmosphere was not significant at all. We then brought the PTFE channel close to the Faraday cup. Specifically, the middle of the top 10 cm of the channel (i.e., the top region onto which water initially contacted the solid surface) was aligned with the center of the opening of the horizontally oriented Faraday cup. The innermost surface of the valley of the V-shaped PTFE channel was at a horizontal distance of 7.5 cm away from the opening of the inner cup of the Faraday cup. The water then flowed across the solid surface as described in the previous paragraph. After the flow stopped, we removed the PTFE channel to a position far away from the Faraday cup and measured the charge of the atmosphere again (i.e., the "final state"). The amount of charge in the atmosphere was determined by taking the difference between the charges measured at the "final state" and the "initial state". Because the PTFE channel was far away from the Faraday cup in both these two states, the measurements of charge were not influenced by the charged surface of the PTFE channel — the difference between the charges measured at the "final state" and the "initial state" represented only the charge dissipated into the atmosphere by the flow of water across the solid surface. The amounts of negative ions in the atmosphere were measured for both the cases when water flowed down the channel as dripping flow and rivulet. When water flowed down as dripping flow, our results showed that even for the relatively low amount of water that flowed across the surface, a substantial amount of negative charge was measured in the atmosphere ("Dissipation during flow" in Figure S7). This amount

of charge was far larger than that generated by the rivulet. As a control experiment, we repeated the same procedure except that we did not flow any liquid down the PTFE channel. In this case, the amount of charge measured was similar to the case when the rivulet flowed down the channel. These results indicated that negative ions were released into the atmosphere when the water flowed across the surface.

When we detected the negative ions in the atmosphere, the experiment involved placing the Faraday cup close to the flow of water down the solid surface. In all cases, the dripping flows of liquid were always positively charged; hence, the positively charged water would not contribute to the amount of negative charge detected by the Faraday cup and the electrometer. In order to completely eliminate the possibility that the water may influence the measurement of negative charge by the Faraday cup, we performed another experiment as follows. The experiment first involved placing the Faraday cup far away from the V-shaped PTFE channel. 15 mL of water at a flowrate of 15 mL/min (i.e., either the dripping flow or the rivulet) then flowed down the V-shaped PTFE channel. After the flow stopped, we measured the charge of the atmosphere by the Faraday cup connected to the electrometer when the V-shaped PTFE channel was still at a location that was far away from the Faraday cup (i.e., charge at the "initial state"). After recording the initial charge, we then moved the charged PTFE channel close to the Faraday cup. The channel was left close to the Faraday cup for 90 s. Importantly, the Faraday cup was placed close to the solid surface that did not have any water flowing across it; this case was different from the previous experiment in which the Faraday cup was placed close to the solid surface with water flowing across it. After that, we removed the channel again to a location far away from the Faraday cup and measured the charge of the atmosphere again (i.e., charge at the "final state"). The amount of charge in the atmosphere was determined by taking the difference

between the charges measured at the “final state” and the “initial state”. Results showed again that a significant amount of negative charge was detected in the atmosphere when water flowed down discontinuously (“Dissipation after flow” in Figure S7). This amount of charge generated was again far larger than that generated by the rivulet or when we did not flow any liquid down the surface. From all these results of our experiments, we conclude that when water flowed across the solid surface, the surface became negatively charged. After the flow stopped, the negative charge then dissipated from the surface into the surrounding atmosphere; this dissipation of negative charge into the atmosphere was the charge detected by the Faraday cup in this experiment. Therefore, negative ions dissipated continuously into the atmosphere when discontinuous liquid flowed across the solid surface.

S15. Influence of Separated Droplets or Continuous Stream before Entering the Tube for Fully Continuous Flow

When the flow is fully continuous in the tube, we found that the power generated is low. Because the fully continuous flow enables the liquid in the tube to be electrically connected continuously to the metallic needle and to the external circuit at point (P2), one concern is that any charge separated in the tube may flow to the metallic needle and discharge via the external circuit. In this case, this discharge at point (P2) reduces the ability of the system to generate electricity. Hence, perhaps the low power generated by the fully continuous flow in the tube may be due to this type of discharge and not due to its specific flow pattern that we discussed in the manuscript.

To study this concern, we performed the experiment as follows. First, the setup still involved flowing water fully continuously through the tube. On the other hand, when the water flowed out of the metallic needle and before it entered the top of the tube, we separated the

stream of water into discrete droplets. This separation of the stream into discrete droplets enabled the continuity to be broken between the metal needle and top of the tube. In this case, any charge separated in the tube is disconnected electrically to point (P2) and would not be discharged.

To achieve this effect, we modified our experimental setup. First, to ensure that the stream of water was in the form of separated drops when it flowed out of the metallic needle, we pumped water out of three syringes instead of one and used metallic needles with a larger diameter so that the outlets of the syringes were larger. By using three syringes, the flowrate out of each syringe was reduced by one-third. The smaller flowrate enabled the water to become separated drops instead of a continuous stream when the water flowed out of the metallic needle. The larger outlet of the metallic needle that served as the outlet of the syringe (i.e., a stainless-steel tube with a large inner diameter of 3 mm) allowed discrete drops to form more readily. To re-unite the separated drops, a funnel was placed on top of the FEP tube. In this way, the separated drops of water that flowed out of all the three syringes were collected simultaneously in the funnel. By combining the three separated streams of drops together, the flowrate in the tube increased; hence, the water flowed as a continuous flow through the FEP tube (i.e., instead of as separated drops). In this experiment, we flowed water at a flowrate of 165 mL/min through a 2 mm FEP tube. A high flow rate of 165 mL/min was required to obtain the fully continuous flow in the FEP tube (i.e., our typical experiment using 80 mL/min produced a discontinuous plug flow due to the relatively low flowrate). Using this setup, we measured the power generated in the cup at the bottom of the tube and at the needle.

Results showed that negligible amounts of power were generated using this setup. The power generated at the metallic needle was a small 6.6×10^{-3} μW (or an efficiency of 5.4×10^{-5} %) and from the water in the cup was a small 4.3×10^{-5} μW (or an efficiency of 8.1×10^{-7} %).

These amounts were similar to that generated by case (V) in which a fully continuous stream of water flowed down the 1 mm tube. Compared to the total amount of 440 μW with an efficiency of 10.4% that we obtained using our typical experimental setup via plug flow, these amounts of power and efficiency are negligible. These results showed that the power generated is negligible as long as the flow is continuous in the tube, regardless of whether the water is separated into discrete drops or not before entering the tube. In other words, it does not matter if the water is electrically connected between the top of the tube and point (P2) for the fully continuous flow in a tube.

S16. Procedures for the Applications of the Electricity Generated by Plug Flow

We showed that flowing a plug flow of water across a solid surface generates a substantial amount of power. In this section, we showed that this highly effective natural phenomenon for generating electricity can be used for many different types of practical applications. One important category of applications involves the use of the electricity generated by the plug flows in tubes. The generated electricity can be used in many applications. Here, we showed that the phenomenon could be used in Sections S16.1 – S16.5 and Section S16.7 six different types of applications. The electricity generated can also be converted by inverters (i.e., the equivalent of transformers that involve direct-current supply) to the power supply with a potential that is commonly used in households and industry while conserving its overall energy. Modern inverters are highly efficient in conserving energy with efficiencies of around ~98%.[27] Because of the significant efficiency of the generation of electricity by the plug flow of >10% and the high efficiencies of the inverters that conserve energy, power for common households can potentially be generated by this simple method. In addition, the simple method can conveniently

be scaled up to involve wide-area harvesting of the potential energy of water from natural sources (e.g., rain or rivers) for powering common households.

The direct use of the charged liquid are also important for a diverse range of applications, including electrostatic coating, manipulation of charged substances in microfluidic channels (e.g., for droplet flow chemistry),[28-30] ink-jet printing,[31, 32] and mass spectrometry.[33] The charge in liquids is also the basis for driving many types of electrostatic devices (e.g., indirectly via electrostatic induction of the charge for producing power).[7, 34-37] In Section S16.6, we showed the application of charged water for the manipulation. Importantly, the manipulation of charged liquid by an electric field is the fundamental ability that is needed for many of the applications of charged water (i.e., including the many applications listed above). Importantly, all these applications are achieved only by the flow of water down simple tubes due to its own weight without the need for any other devices or sources of energy. The energy-harvesting setup of our experiments only involved the water towers for storing the water, FEP tubes, containers for collecting the water after flowing through the tubes, water, and wires. No equipment, device, or electricity was needed for the operation. Hence, the operation can be performed conveniently in any circumstances.

S16.1 Continuous illumination of LEDs

We showed experimentally that the electricity generated by the plug flow of water down the tubes can effectively maintain illumination of light-emitting diodes (LEDs) continuously. Because the generation of energy involves simply allowing the water to fall down the tube by its own weight, the process can be driven by natural sources, such as rain or rivers. Therefore,

illumination can be obtained easily anywhere for practical purposes by simply flowing water down a tube.

The procedure for generating the plug flow of water down the FEP tubes was similar to that described in the Methods. We used two syringe pumps with four 50-mL syringes mounted on them and four FEP tubes in this experiment. Water towers were tested to perform similarly in this demonstration. The syringe pumps were operated at a rate of 80 mL/min. Because four syringes and four tubes were used, four streams of water flowed through the tubes separately. The water that flowed out of each of the outlets of the four tubes was collected by a separate glass beaker (100 mL). Before the experiment, each glass beaker was prefilled with 30 mL of uncharged deionized water. A wire was immersed in the prefilled water in each of the four beakers to maintain electrical contact (i.e., point (P1) as illustrated in Figure 1c). The other end of each of these wires was connected electrically to 50 capacitors (i.e., connected in series on a breadboard) and then to ground. Each capacitor had a capacitance of 39 nF. A similar set of connections was made at point (P2) as illustrated in Figure 1c. Specifically, a wire was connected separately to each of the four metallic needles fixed on the syringes. The other end of each of the four wires was connected electrically to 50 capacitors (i.e., connected in series on a breadboard) and then to ground. Each capacitor also had a capacitance of 39 nF. 27 mL of water was flowed through each FEP tube to charge up the capacitors for a duration of 20 s (i.e., with a flow rate of 80 mL/min). After the water stopped flowing, the configuration of the capacitors was then switched from series to parallel for increasing the current for lighting up the LEDs. The capacitors in parallel were connected in series to 12 LEDs and resistors with a total resistance of 1.6 MΩ. The charging of capacitors by the plug flow for 20 s was found to light up the 12 LEDs brightly for at least 20 s. Because it took the same amount of time to light up the LEDs and to

flow the plug flow through the tube, this experiment showed that the power generated by the plug flow can light up the LEDs continuously. In fact, we found that about only one-third of the charge stored in the capacitors generated by the plug flow discharged after lighting up the 12 LEDs brightly for 20 s. As shown in Figure S8, the plug flow charged up the capacitors from zero to 38.4 V in 20 s, whereas the voltage of the capacitors only dropped from 38.4 V to 26.4 V when discharging and lighting up the LEDs for 20 s. The two-third of the charge left indicated that there was more power available in the capacitors after lighting up the LEDs.

S16.2 Glow illumination

We also showed experimentally that the electricity generated by the plug flow of water down a tube effectively produces illumination via the mechanism used by common types of lamps (e.g., fluorescent lamps). The procedure for generating the plug flow of water down the FEP tubes was similar to that described in the Methods. To obtain a more stable illumination, we used two water towers (i.e., two PVC pipes sealed on one end) and two FEP tubes. The water towers were filled to a height of 165 cm for allowing water to flow out of the towers at a rate of 80 mL/min. Each water tower allowed water to flow into one of the tubes separately. The water that flowed out of both the outlets of the two FEP tubes was collected by a single glass beaker (1000 mL). Before the experiment, the glass beaker was filled with 100 mL of uncharged deionized water.

The experimental setup for generating the electricity involved two metallic tips of two syringe needles that faced each other as illustrated in Figure 6c in the main text. Each of the tips tapered off asymmetrically on one side; the longest side of the tapered tip was 6 mm longer than the opposite shortest side. These two metallic tips served as the electrodes for generating the electricity. One end of a wire was connected to one of the metallic tips that served as the positive

electrode, whereas the other end of this wire was immersed in the deionized water in the 1000 mL glass beaker. One end of another wire was connected to the other metallic tip that served as the negative electrode; the other end of this wire was connected electrically to both the needles that were fixed to the two outlets of the water towers for directing the flow out of the water towers and into the tubes. Based on this energy-harvesting setup, the tips of the two syringe needles were placed 0.8 mm apart and in an atmosphere filled with helium gas. When the water flowed, we observed a relatively stable glow across the tips of the two syringe needles (Figure 6c in the main text and Movie S8). This glow can serve as a source of illumination.

S16.3 Performing Chemical Reactions in a Liquid Medium

Electricity can be used to perform chemical reactions in a liquid medium; the reactions include the decomposition of organic molecules for important applications such as contaminated soil, wastewater treatment, and air purification.[38-40] However, the use of a power supply is energy-intensive and requires the use of an equipment. We showed experimentally that the electricity produced by the plug flow of water down a tube is also able to perform chemical reactions. This method is simple to perform and does not require any other supply of electricity or equipment.

For this experiment, we chose the molecule, methylene blue, for performing the reaction. It was previously shown that an aqueous solution that contained methylene blue tended to decolorize when exposed to electricity; hence, the progress of the reaction can be conveniently monitored by the change in color. An aqueous droplet (15 μL) that was composed of 10 mg/L of methylene blue dissolved in deionized water was placed on a stainless-steel plate. For performing the reaction that involved the methylene blue, we applied the electricity generated by the plug flow down a FEP tube onto the aqueous droplet. The electricity was generated by

flowing water from one water tower (i.e., PVC pipe) into a FEP tube at a rate of 80 mL/min (i.e., as described in Methods). The FEP tube had a length of 32 cm and a diameter of 2 mm. The water that flowed out of the FEP tube was collected by a plastic tray (10 L). Before the experiment, the tray was filled with 500 mL of uncharged deionized water. One end of an electrical wire was immersed in the deionized water in the tray, whereas the other end of the wire was connected to a metallic syringe needle (inner diameter of 0.4 mm). The tip of this metallic syringe needle was placed 1 mm above the top of the center of the aqueous droplet that contained methylene blue. The metallic syringe needle that served as the outlet of the water from the water tower was electrically connected to the stainless-steel plate that the aqueous droplet rested on. The experiment was performed under ambient air conditions.

When water flowed through the tubes and into the tray, we observed frequent perturbations to the shape of the droplet during the process. The droplet was initially blue due to the methylene blue. After two hours, we found that the droplet was completely decolorized (Figure 7b in the main text). A control experiment was performed with exactly the same setup except that water did not flow through the FEP tube. In this case, the droplet was observed to remain blue after two hours as expected. The aqueous solutions from both the actual experiment with the flow of water and the control experiments were diluted to 300 μL by deionized water (i.e., to obtain the sufficient volume for analysis) and analyzed by ultraviolet–visible spectroscopy (UV-Vis). The analysis of the solution obtained from the control experiment (i.e., when water did not flow through the FEP tube) showed that the absorption peak of MB^+ at 664 nm was present. However, the analysis of the solution obtained from the actual experiment (i.e., when water flowed through the FEP tube) showed that the absorption peak completely

disappeared. These results showed that the methylene blue was completely reacted due to the application of the electricity generated by the plug flow of water down the tube.

S16.4 Generation of Radicals

Radicals are highly reactive species that are involved in a wide range of reactions, both in synthetic chemistry and in nature. Many important applications and useful reactions are based on the high reactivity of radicals; for example, they are used for initiating chemical reactions (i.e., for producing useful molecules), polymerizations, and decomposition of chemicals.[39-42] In addition, radicals are useful for medical treatment because of their ability to damage pathogens and cancer cells.[43, 44] Common methods of generating radicals include electromagnetic irradiation, redox reaction, and enzymatic reactions. These methods, however, have the disadvantages that they require substantial amount of energy, specialized chemicals, materials, and/or biological enzymes. In contrast, the phenomenon that involves the plug flow of water down the tubes does not require any external input of energy (i.e., the energy can be obtained freely from nature, such as rain or river) or special types of substances. In addition, the method is simple and convenient to operate.

In this experiment, we used 2,2-diphenyl-1-picrylhydrazyl (DPPH) as the radical scavenger for detecting the production of radicals. DPPH reacts readily with radical species and forms the reduced form of the molecule (i.e., DPPH-H). The lightening of the color during the reduction of DPPH is a standard method for detecting radical species in the solution. We first prepared a solution of DPPH dissolved in *N,N*-dimethylformamide (DMF) at a concentration of 500 μM. A droplet of 5 μL of the DPPH solution was placed on a stainless-steel plate. A needle

with a sharp tip was oriented vertically and placed 1 mm above the center of the droplet containing DPPH.

For the energy-harvesting components, we used two water towers (i.e., two PVC pipes) and two FEP tubes as described in Section S16.2. The water towers were filled to a height of 165 cm for allowing water to flow out of the towers at a rate of 80 mL/min. Each water tower allowed water to flow into one of the tubes separately. The water that flowed out of both the outlets of the FEP tubes was collected by a plastic tray (10 L). Before the experiment, the tray was filled with 500 mL of uncharged deionized water. One end of an electrical wire was immersed in the deionized water in the tray, whereas the other end of the wire was connected to the stainless-steel plate that the droplet of DPPH solution rested on. On the other hand, the needle with the sharp tip placed 1 mm above the top of the center of the droplet of DPPH solution was electrically connected to the metallic syringe needles that served as the outlets of the water from the water towers. When water flowed from the water towers through the tubes and into the tray, we observed frequent perturbations to the shape of the droplet. The experiment was conducted for 4 h under ambient air conditions. A control experiment was performed by repeating the procedure with the same setup except that no water flowed through the FEP tubes (i.e., the droplet of DPPH solution also rested on the stainless-steel plate for 4 h). The droplets of DPPH solution were diluted to 250 µL (i.e., by adding more DMF) and analyzed by ultraviolet–visible spectroscopy (UV-Vis).

The droplet of DPPH solution was originally purplish red (Figure 7c in the main text). The treatment caused the droplet to change its color gradually. After 4 h, the droplet became pale yellow. For the control experiment, the droplet of DPPH solution remained purplish red. Hence, the colors were distinctly different for the treated and untreated droplets of DPPH solution. This

change in color from purplish red to pale yellow is indicative that the reduction of the DPPH radical occurred. The results of the analysis by the UV-Vis showed accordingly that the spectra obtained for the treated and untreated droplets of DPPH solution were very different. The characteristic absorption peak at ~480 nm obtained for the untreated droplet of DPPH solution disappeared when the droplet of DPPH solution was treated with the electricity generated by the plug flow of water down the tubes. Previous studies reported that the spectrum obtained from our analysis for the treated droplet of DPPH without the peak at ~480 nm indicated that DPPH had reacted and reduced to DPPH-H completely.[45, 46] For example, the analyses performed by the previous studies involved the measurements of the absorbance of the pure reduced form of DPPH-H and the complete reaction of the DPPH into DPPH-H by an excess amount of cysteine. In these cases, the authors obtained spectra that are similar to our result for the treated droplet of DPPH solution. Therefore, the simple phenomenon of having the plug flow of water down a simple tube is able to generate a substantial amount of radicals.

S16.5 Increasing Surface Wettability of Polymer

Increasing the wettability (i.e., hydrophilicity) of the surface of polymers is important in many industries (e.g., medical and textile).[47-49] Common methods for increasing the wettability of surfaces of polymers including plasma treatment, corona discharge, UV irradiation, chemical vapor deposition, silanization, grafting of polymers, and coating a layer of hydrophilic material.[48, 50, 51] However, these processes usually involve large amount of energy, chemicals, and/or materials. In addition, they often require specialized equipment, high vacuum (e.g., ~0.1 mbar), power supply, or the handling of dangerous chemicals.

We showed experimentally that the plug flow of water down a FEP tube can increase the wettability of polymers effectively. This simple phenomenon does not require any input of artificial sources of energy, chemicals, or specialized types of materials. It also does not require any equipment, special conditions, or complicated procedures. The energy can be obtained freely from natural sources such as rain or rivers for driving the process.

We chose PDMS for our demonstration because it is a very common type of elastomer that has many desirable properties such as biocompatibility, non-toxicity, ease of fabrication, chemical inertness, high transparency, good softness, elasticity, and low cost.[52, 53] Because of its many desirable properties, it has been used in many important applications, including biomedical devices and fluidic systems (e.g., microfluidics). Despite the usefulness of PDMS, the hydrophobicity and low surface energy of PDMS have limited the applicability of the polymer. For example, the hydrophobicity of PDMS has made the fabrication of microfluidic devices that are based on aqueous solutions difficult. Another example involves the adsorption of protein or analyte onto the hydrophobic surface of PDMS that can lead to the considerable loss of molecules; this loss is a severe problem for the transport of solutions and the determination of concentrations.[50, 54] Therefore, it is important to modify the surface of PDMS from hydrophobic to hydrophilic for many important applications.

The PDMS was prepared by first mixing the pre-polymer liquid (Sylgard 184) and curing agent vigorously in a weight ratio of 10:1 respectively in a Petri dish. This liquid in the Petri dish was placed in a vacuum environment for 45 min to remove any air bubbles trapped in the liquid. 3 g of the liquid mixture was then placed on a spin coater. The spin coater was operated at 400 rpm for one minute. The liquid was allowed to polymerize in an oven at 70 °C for 1.5 h. The polymerized layer of PDMS had a thickness of 200 μm. The PDMS was washed by immersing it

in triethylamine (TEA) for 10 h, followed by immersing it in deionized water for another 10 h. Washing of the PDMS was needed to remove any uncrosslinked or low molecular-weight polymer.[55] It was dried in an oven at 90 °C overnight. Finally, we cut the film of PDMS into smaller pieces of 2 mm by 2 mm for the experiment.

The film of PDMS was placed on top of a stainless-steel plate. The surface of the stainless-steel plate was covered with layers of insulating tapes except an exposed region that was slightly larger than the film of PDMS (i.e., 2 mm by 3 mm). The film of PDMS was placed at the center of the exposed region of the stainless-steel plate. A needle with a sharp tip was oriented vertically and placed 1 mm above the center of the film of PDMS.

For the energy-harvesting components, we used two water towers (i.e., the PVC pipes) and two FEP tubes as described in Section S16.2. The water towers were filled to a height of 165 cm for allowing water to flow out of the towers at a rate of 80 mL/min. Each water tower allowed water to flow into one of the tubes separately. The water that flowed out of both the outlets of the FEP tubes was collected by a plastic tray (10 L). Before the experiment, the tray was filled with 500 mL of uncharged deionized water. One end of an electrical wire was immersed in the deionized water in the tray, whereas the other end of the wire was connected to the stainless-steel plate that the film of PDMS rested on. On the other hand, the needle with the sharp tip placed 1 mm above the center of the film of PDMS was electrically connected to the two metallic syringe needles that served as the outlets of the water from the water towers. When water flowed through the tubes and into the tray, frequent electric sparks were observed to be emitted from the needle with the sharp tip and onto the surface of the PDMS (Figure 7d in the main text and Movie S9). We found from our investigations that it is advantageous to have the sharp tip in close proximity to the film (i.e., 1 mm). When the distance was farther than 1 mm,

the occurrence of the sparks was observed to be less frequent. The surface of the PDMS was treated by this method for 1 h.

After treatment, the contact angle of water of the surface of PDMS was measured. The measurement involved a small sessile drop of ~1 μL of water placed on the surface of the treated PDMS. A microscope (Leica M205C equipped with a 2.0× achromatic objective and a Leica MC170 HD camera) was used to capture the images of the drop of water resting on the surface. The contact angle was then determined by using an image processing software (ImageJ). As a control experiment, we prepared a fresh piece of PDMS and measured its contact angle of water (i.e., a piece of PDMS that was not treated). Our results showed that the contact angle of water decreased from 109° for the untreated piece of PDMS to 37° for the treated piece of PDMS — a large decrease in the contact angle. Hence, the surface of PDMS had changed effectively from hydrophobic to hydrophilic. This large difference in contact angle of water is comparable or better than many other common and standard methods for treating surfaces, including corona discharge,[56] plasma cleaning,[47, 55] UV treatment,[57] polymer coating, grafting of polymers (e.g., poly(ethylene glycol)),[58-61] and surface functionalization (e.g., silanization).[54] Comparing to these methods that expend substantial amounts of energy, chemicals, and/or materials, it is thus surprising that this simple phenomenon of the plug flow of water down a simple tube is effective for increasing the wettability of surfaces.

The chemical compositions of the treated and untreated pieces of PDMS were analyzed by X-ray photoelectron spectroscopy (XPS). XPS spectra were recorded on a Kratos AXIS UltraDLD system (Kratos Analytical, UK) with Al Kα excitation radiation (1486.71 eV). The pressure in the analysis chamber was maintained at 10^{-6} Pa during the analysis. Survey spectrum and high-resolution scans of C 1s, O 1s, and Si 2p were generated for quantitative analyses. After

treating the surface of PDMS, the analysis showed that the composition of oxygen increased from 35.3% to 36.4%, carbon decreased from 47.5% to 46.4%, whereas silicon remained unchanged (Figure 7d in the main text). The high-resolution scan of Si 2p of the untreated piece of PDMS showed the two typical peaks that represented the bonding of one silicon with four oxygen ($SiO_{4/2}$) at 103.9 eV (i.e., labeled as "Peak 1" in Figure S9a) and the bonding of one silicon with two oxygen ($(CH_3)_2SiO_{2/2}$) at 102.5 eV (i.e., labeled as "Peak 2" in Figure S9a).[56, 57] After treatment, the analysis showed a significant increase in the proportion of ($SiO_{4/2}$) compared to ($(CH_3)_2SiO_{2/2}$) (Figure S9b). Therefore, these results showed that the increase in oxygen content after treatment was due to the increase in the number of oxygen that bonded with silicon. Many previous studies that used common methods (e.g., corona discharge and plasma treatment) for increasing the wettability of PDMS reported similar results. The chemical mechanism discussed in these previous studies involved first the cleavage of the Si-CH_3 bonds on the surface of PDMS and then the formation of the hydrophilic Si-OH groups that replace the Si-CH_3 groups. It is the hydrophilic Si-OH groups that allow the wettability of PDMS to be increased.[47, 56, 62, 63]

S16.6 Manipulation of Charged Liquid

Applications that require the manipulation of charged liquid always rely on using electrical power supplies. The operation usually involves charging the liquid by one electrical power supply and charging an electrode separately by another electrical power supply. The electrode is then used to manipulate flexibly the charged liquid. This operation, however, requires the equipment, power, and dangerous handling of the electrical power supply. For our demonstration, we showed that the liquid could be charged highly for manipulation without the

use of any equipment or power. In particular, no power supply is needed for both the charging of the liquid and the electrode for manipulation. The ability to perform manipulation using a simple and convenient method without any equipment or supply is desirable for many applications (e.g., low-cost diagnostic devices).

We charged the liquid highly by using a procedure that was similar to that described in the Methods. A polyvinyl chloride (PVC) pipe with an inner diameter of 8 cm was used as the water tower. It was erected vertically. The bottom end of the pipe was sealed with a custom-made cap that fitted perfectly with the pipe and prevented any water from flowing out of the pipe at the bottom. A hole of 5 mm in diameter was made at the bottom of the pipe (i.e., 10 cm above the bottom end of the cap); this hole served as the outlet for the water to flow out of the pipe. To direct the flow of water out of the pipe specifically to the tube, a metallic needle (length: 15 cm and inner diameter: 0.838 mm) was fixed onto the hole via a connector. We used an L-shaped needle (i.e., both sections of the L-shape were almost equal in length) for directing the flow of water; hence, the water initially flowed horizontally out of the pipe but changed to flowing vertically downward after the L-shaped elbow. The water then flowed vertically into the top of a FEP tube. The vertically oriented FEP tube had an inner diameter of 2 mm and a length of 32 cm. We filled the PVC pipe to a height of only 12 cm with deionized water for flowing water at a slow rate of 1 mL/min. Due to the low flowrate, the plug flow was obtained as the water flowed down the tube. The metallic syringe needle that was fixed to the outlet of the pipe was connected electrically to ground.

A slab of solid surface (7.6 × 2.6 × 0.3 cm) that was placed horizontally and 8 mm vertically below the bottom end of the FEP tube served as the platform for the motion of the charged water drips. The surface of the platform was coated with a layer of hydrophobic film

(Neverwet®); this coating prevented water from adhering onto the surface. In a separate step, a slab of poly(methyl methacrylate) (PMMA; 7.6 × 2.6 × 0.3 cm) was charged positively to around 3 – 4 nC by an antistatic (Zerostat) gun. This positively charged PMMA was erected vertically. It was placed adjacent to one of the edges of the horizontal platform as showed in Figure 7e in the main text and 3 mm horizontally away from the outlet of the FEP tube. Whenever the charged water drips fell out of the FEP tube, we observed that they were quickly repelled away from the positively charged PMMA and moved in the opposite direction (Figure 7e and Movie S10). The manipulation was achieved continuously and repeatedly for the constant stream of water drips that flowed out of the tube. This experiment thus showed that the fundamental mechanism of charging water by a plug flow could effectively charge water highly for manipulation. In addition, it was able to generate a continuous supply of highly charged drips for manipulation. This experiment showed that no power supply was needed for both the charging of the liquid and the electrode used for the manipulation (i.e., the piece of polymer charged by a simple Zerostat gun).

S16.7 Charging Materials

Polymers with charged surfaces (e.g., electrets) are commonly used in a wide range of applications, including energy harvesting, transducers, transistors, sensors, air-filters, electrostatic separators, and coating.[64, 65] Common methods for charging the surfaces of materials (e.g., polymers) include corona discharge, electron beam injection, and thermal charging.[64] We showed experimentally that the power generated by the plug flow of water down a tube can be used to charge the surfaces of materials. This simple process does not require any equipment, power supply, or complicated operation.

The procedure for generating the plug flow of water down the FEP tube was similar to that described in the Methods. This experiment was performing by using the water tower made of the PVC pipe and a FEP tube. The water tower was filled to a height of 165 cm for allowing water to flow out of the tower at a rate of 80 mL/min. The metallic needle fixed at the outlet (i.e., bottom) of the water tower was grounded electrically. The water flowed out of the tower and into the FEP tube via the metallic needle. The water that flowed out of the outlet of the vertically oriented tube was collected by a glass beaker (250 mL). Before the experiment, the glass beaker was filled with 20 mL of uncharged deionized water.

We charged surfaces of materials using the plug flow of water down a tube for four types of polymers: PTFE, polypropylene (PP), polyvinyl chloride (PVC), and polycarbonate (PC). They were thin rectangular slabs of polymers with dimensions of 7.6 × 2.6 × 0.3 cm. These polymers were selected because they are common materials used in a diverse range of applications. In addition, they have important applications when charged. For example, charged PP is used as electrostatic fillers in surgical and N95 masks; these electrostatic fillers enhance the performance of the masks by allowing more particles to be trapped in the layers by electrostatic forces.[66, 67] On the other hand, charged PC is commonly used as the material for air filters.[68] Charged PTFE is commonly used in many electrostatic-based devices.[65]

For charging the surface of the polymer, the polymer was first placed horizontally on top of the surface of a large stainless-steel plate. This stainless-steel plate was electrically grounded. A stainless-steel needle with a sharp tip was oriented vertically and placed 1 mm above the center of the slab of polymer. One end of an electrical wire was immersed in the 250 mL glass beaker that was pre-filled with water. The other end of this electrical wire was connected to the

stainless-steel needle placed 1 mm above the slab of polymer. A total volume of 50 mL was allowed to flow from the water tower through the tubes for charging the slab of polymer.

The charges of the polymers before and after charging by the power produced by the plug flow of water down the tube were measured by a Faraday cup connected to an electrometer (Keithley, model 6514). Results showed that our method was able to charge all the four types of polymers from almost uncharged or slightly negatively charged to substantial amounts of positive charge of around +10 nC — these amounts of charge were generated after flowing only 50 mL of water through the tube (Figure 7f). Importantly, the plug flow of this small volume of water down the tube was able to charge the materials to amounts that were comparable to other commonly used methods. For example, multiple studies have reported the charging of materials with similar amounts of charge densities as our method using corona discharge generated by electrical power supplies.[69, 70] These similar amounts of charge reported in these previous studies are useful in practical applications such as sensors, filters, and electret microphones.

S17. Charge Generation by a Small Volume of Water Representative of a Rain Drop

Rain contains a massive amount of energy; however, that energy is currently largely untapped in the world. In the main text, we showed that a continuous stream of plug flow that flowed at a rate of 80 mL/min through the FEP tube generated a substantial amount of power. To show that our system can harvest the energy of rain drops, we repeated the experiment using only a very small volume of water of 15 μL instead of a steady continuous stream of plug flow. This small volume of water is representative of a single rain drop with a diameter of 3 mm. In this experiment, we regulated the velocity of the small volume of water in a 2 mm FEP tube (length of 32 cm) via a stream of compressed air at 0.6 m/s (which is around the same order of magnitude as the 80

mL/min that we typically used in our experiments). This velocity is highly conservative for showing the feasibility of our technology for harvesting rain energy; the typical velocity of rain drops (i.e., at terminal velocity) is widely known to be more than an order of magnitude higher at ~9 m/s. We found that the charge per mass generated by this small volume of water through the tube was 63.5 nC/g; this amount is similar to that generated by our typical setup that consisted of the continuous stream of plug flow passing through the tube at a flow rate of 80 mL/min at 62 nC/g. Hence, we showed that a small volume of water typical of a rain drop can also generate the same amount of power per mass of water as the continuous plug flow.

S18. Harvesting Large-Scale Energy from Nature by Scaling Up

Practically useful amounts of electricity can be obtained readily by scaling up our system that involves plug flow in a tube in three dimensions. As an illustration, we discuss the generation of 5W of electricity using our technology. For generating 5W, we could conceptually construct a setup that consists of 20 vertical stages; each stage consists of a bundle of 500 tubes packed horizontally. We note that because our system involves very small 2 mm tubes, the overall lateral size of the setup is small even when many tubes are bundled together. Assuming a 1 mm wall thickness for each 2 mm inner diameter tube and square-packing structure, the bundle of 500 tubes covers only a very small lateral area of 7 cm × 7 cm. The tubes are each 32 cm long. Hence, the total height of 20 stages will be around a practically reasonable height of 7 m. With this setup, the water flow rate required is only 40 L/min (i.e., water flowing into 500 tubes at a flow rate of 80 mL/min into each tube)—hence, a practically useful power of 5W can be generated by only a flowrate of 40 L/min using this conceptual setup.

Following the discussion above, large amounts of electricity can be generated by constructing a correspondingly large physical structure using this technology. This approach to harvesting large amounts of energy is thus very similar to that of other types of renewable energy, such as hydroelectric power or solar panels, in which scaling up is needed for harvesting large amounts of energy.

S19. Verifying the method of quantification of power

In our experiments, we quantified the amount of power generated by the plug flow of water by measuring the potential difference across resistors connected to the system. Specifically, we determined that the plug flow generated a potential difference of 4 – 5 kV across resistors with a total resistance of 61 GΩ (e.g., Figure 1e). To verify whether this measurement was accurate, we compared this measurement with a known potential source via using a high-voltage power supply (Gamma High Voltage, RR30-5R/230). We used the high-voltage power supply with a pre-determined potential to generate electricity across resistors with total load resistances of either 31 GΩ, 41 GΩ, or 61 GΩ (i.e., the resistances that we typically used in our experiments), and then to ground. We systematically varied the potential of the power supply from 0 – 5 kV and measured the potential difference (i.e., via an electrometer) across the resistors. In other words, we used exactly the same electrical circuit and method of measurement that we used for our experiments involving the plug flow of water, except that instead of using the electricity generated by the plug flow of water, we used the high-voltage power supply. Results of this calibration showed that the potential difference of the electrical circuit supplied by the plug flow of water matched exactly the pre-determined potential supplied by the high-voltage power supply (Figure S10). Specifically, when we determined a potential difference of 5000 V in the electrical

circuit with resistance of 61 GΩ supplied by the plug flow of water, we also needed to supply 5000 V of potential by the high-voltage power supply. Therefore, our measurements were accurate.

S20. Materials

Dimethyl sulfoxide (DMSO), 1,3-dimethyl-2-imidazolidinone (DMI), hydrogen peroxide (H_2O_2; 35%), sodium hydroxide (NaOH), sodium chloride (NaCl), poly(ethyleneimine) (PEI), poly(allylamine hydrochloride) (PAH), poly(diallyldimethylammonium chloride) (PDDA), poly(sodium 4-styrenesulfonate) (PSS), fluorescein isothiocyanate isomer I (FITC), methylene blue (MB), triethylamine (TEA), 2,2-diphenyl-1-picrylhydrazyl (DPPH), and *N,N*-dimethylformamide (DMF) were purchased from Sigma-Aldrich. Acetonitrile (ACN), ethanol (analytical grade and HPLC grade), and hydrogen chloride (HCl) were purchased from Fisher Scientific Pte Ltd. Sulfuric acid (H_2SO_4; analytical grade) was purchased from VWR Chemicals. Sylgard 184 silicone elastomer kit was purchased from Dow Corning Co. (US). This kit was used for preparing the poly(dimethylsiloxane) (PDMS). Ecoflex™ silicone rubber curing kit was purchased from Smooth-On. Neverwet® superhydrophobic coating was purchased from Rust-Oleum. Fluorinated ethylene propylene (FEP) tubes and polytetrafluoroethylene (PTFE) V-shaped channels were purchased from Latech Scientific Supply Pte Ltd. Slabs of PTFE, polypropylene (PP), polyvinyl chloride (PVC), polycarbonate (PC), and stainless-steel plates were purchased from Microtech Engineering Pte Ltd. Slabs of poly(methyl methacrylate) (PMMA) were purchased from DAMA Trading Pte Ltd. Glass tubes were purchased from United Scientific Equipment Pte Ltd. Corporation. PVC pipes (each 150 – 165 cm long) were purchased from a local hardware store in Singapore. Glass coverslips (24 mm × 50 mm and

thickness of 0.13-0.16 mm) were purchased from Sigma-Aldrich. Argon, nitrogen, oxygen, and helium gas were purchased from Air Liquide Singapore Pte Ltd. Ultrapure deionized water with a resistivity of 18 MΩ·cm was used in all experiments.

Captions of Movies

Movie S1. Plug flow in a 2 mm FEP tube.

Movie S2. Plug-dripping flow in a 3 mm FEP tube.

Movie S3. Dripping flow in a 6 mm FEP tube.

Movie S4. Rivulet in a 6 mm FEP tube.

Movie S5. Full flow in a 1 mm FEP tube.

Movie S6. Dripping-rivulet flow transition.

Movie S7. Continuous illumination of LEDs by plug flows of water down four 2 mm FEP tubes.

Movie S8. Bright glow across the tips of two needles generated by plug flows of water down two 2 mm FEP tubes.

Movie S9. Frequent sparks emitted from a metallic tip for the surface treatment of PDMS by plug flows of water down two 2 mm FEP tubes.

Movie S10. Water drips that charged effectively positively by the plug flow down a 2 mm FEP tube repelled rapidly away from a vertically erected slab of positively charged polymer (located on the left side of the video).

References

(1) do Nascimento, F. H.; Moraes, A. H.; Trazzi, C. R. L.; Velasques, C. M.; Masini, J. C. Fast Construction of Polymer Monolithic Columns Inside Fluorinated Ethylene Propylene (FEP) Tubes for Separation of Proteins by Reversed-Phase Liquid Chromatography. *Talanta* **2020**, *217*, 121063.

(2) Jung, M. R.; et al. Validation of ATR FT-IR to Identify Polymers of Plastic Marine Debris, Including Those Ingested by Marine Organisms. *Mar. Pollut. Bull.* **2018**, *127*, 704–716.

(3) Morra, M.; Occhiello, E.; Garbassi, F. Contact Angle Hysteresis in Oxygen Plasma Treated Poly(tetrafluoroethylene). *Langmuir* **1989**, *5*, 872–876.

(4) Busscher, H. J.; Stokroos, I.; Van Der Mei, H. C.; Rouxhet, P. G.; Schakenra Ad, J. M. Preparation and Characterization of Superhydrophobic FEP-Teflon Surfaces. *J. Adhes. Sci. Technol.* **1992**, *6*, 347–356.

(5) Kim, Y.-P.; Hong, M.-Y.; Kim, J.; Oh, E.; Shon, H. K.; Moon, D. W.; Kim, H.-S.; Lee, T. G. Quantitative Analysis of Surface-Immobilized Protein by ToF-SIMS: Effects of Protein Orientation and Trehalose Additive. *Anal. Chem.* **2007**, *79*, 1377-1385.

(6) Ye, C.; et al. A Hydrophobic Self-Repairing Power Textile for Effective Water Droplet Energy Harvesting. *ACS Nano* **2021**, *15*, 18172–18181.

(7) Lin, Z.; Cheng, G.; Lee, S.; Pradel, K. C.; Wang, Z. L. Harvesting Water Drop Energy by a Sequential Contact-Electrification and Electrostatic-Induction Process. *Adv. Mater.* **2014**, *26*, 4690–4696.

(8) Jeon, S.-B.; Kim, D.; Yoon, G.-W.; Yoon, J.-B.; Choi, Y.-K. Self-Cleaning Hybrid Energy Harvester to Generate Power from Raindrop and Sunlight. *Nano Energy* **2015**, *12*, 636–645.

(9) Lai, Y.; Hsiao, Y.; Wu, H.; Wang, Z. L. Waterproof Fabric-Based Multifunctional Triboelectric Nanogenerator for Universally Harvesting Energy from Raindrops, Wind, and Human Motions and as Self-Powered Sensors. *Adv. Sci.* **2019**, *6*, 1801883.

(10) Chen, K.; et al. Fabric-Based Teng Woven with Bio-Fabricated Superhydrophobic Bacterial Cellulose Fiber for Energy Harvesting and Motion Detection. *Adv. Funct. Mater.* **2023**, *33*, 2304809.

(11) van der Heyden, F. H.; Bonthuis, D. J.; Stein, D.; Meyer, C.; Dekker, C. Power Generation by Pressure-Driven Transport of Ions in Nanofluidic Channels. *Nano Lett.* **2007**, *7*, 1022–1025.

(12) Burgreen, D.; Nakache, F. R. Efficiency of Pumping and Power Generation in Ultrafine Electrokinetic Systems. *J. Appl. Mech.* **1965**, *32*, 675–679.

(13) van der Heyden, F. H.; Bonthuis, D. J.; Stein, D.; Meyer, C.; Dekker, C. Electrokinetic Energy Conversion Efficiency in Nanofluidic Channels. *Nano Lett.* **2006**, *6*, 2232–2237.

(14) Chang, C.-C.; Yang, R.-J. Electrokinetic Energy Conversion in Micrometer-Length Nanofluidic Channels. *Microfluid. Nanofluid.* **2009**, *9*, 225–241.

(15) Vaccaro, A.; et al. Structure of an Adsorbed Polyelectrolyte Monolayer on Oppositely Charged Colloidal Particles. *Langmuir* **2009**, *25*, 4864–4867.

(16) Hierrezuelo, J.; Szilagyi, I.; Vaccaro, A.; Borkovec, M. Probing Nanometer-Thick Polyelectrolyte Layers Adsorbed on Oppositely Charged Particles by Dynamic Light Scattering. *Macromolecules* **2010**, *43*, 9108–9116.

(17) Yang, M.; Lu, S.; Lu, J.; Jiang, S. P.; Xiang, Y. Layer-by-Layer Self-Assembly of PDDA/PWA–Nafion Composite Membranes for Direct Methanol Fuel Cells. *Chem. Commun.* **2010**, *46*, 1434–1436.

(18) Singh, S.; et al. Neutron Reflectometry Characterization of PEI–PSS Polyelectrolyte Multilayers for Cell Culture. *Soft Matter* **2012**, *8*, 11484–11491.

(19) Zhang, S.; et al. Nanopapers of Layer-by-Layer Nanotubes. *J. Mater. Chem. B* **2016**, *4*, 7651–7661.

(20) ISO. Optical Methods. *ISO 13099-2:2012*; Geneva, Switzerland, 2012.

(21) ASTM International. ASTM E2865-12(2018); West Conshohocken, PA, USA, 2018.

(22) McCarty, L. S.; Whitesides, G. M. Electrostatic Charging Due to Separation of Ions at Interfaces: Contact Electrification of Ionic Electrets. *Angew. Chem., Int. Ed.* **2008**, *47*, 2188-2207.

(23) Rezende, C. A.; Gouveia, R. F.; da Silva, M. A.; Galembeck, F. Detection of Charge Distributions in Insulator Surfaces. *J. Phys.:Condens. Matter* **2009**, *21*, 263002.

(24) Galembeck, F.; Burgo, T. A. L.; Balestrin, L. B. S.; Gouveia, R. F.; Silva, C. A.; Galembeck, A. Friction, Tribochemistry and Triboelectricity: Recent Progress and Perspectives. *RSC Adv.* **2014**, *4*, 64280-64298.

(25) Das-Gupta, D. K. Electrical Properties of Surfaces of Polymeric Insulators. *IEEE Trans. Electr. Insul.* **1992**, *27*, 909-923.

(26) Wiles, J. A.; Fialkowski, M.; Radowski, M. R.; Whitesides, G. M.; Grzybowski, B. A. Effects of Surface Modification and Moisture on the Rates of Charge Transfer between Metals and Organic Materials. *J. Phys. Chem. B* **2004**, *108*, 20296-20302.

(27) Scarabelot, L. T.; Rambo, C. R.; Rampinelli, G. A. A Relative Power-Based Adaptive Hybrid Model for DC/AC Average Inverter Efficiency of Photovoltaics Systems. *Renew. Sustain. Energy Rev.* **2018**, *92*, 470–477.

(28) Sun, Y.; Huang, X.; Soh, S. Solid-to-Liquid Charge Transfer for Generating Droplets with Tunable Charge. *Angew. Chem., Int. Ed.* **2016**, *55*, 9956–9960.

(29) Link, D. R.; et al. Electric Control of Droplets in Microfluidic Devices. *Angew. Chem., Int. Ed.* **2006**, *45*, 2556–2560.

(30) Zhou, H.; Yao, S. Electrostatic Charging and Control of Droplets in Microfluidic Devices. *Lab Chip* **2013**, *13*, 962–969.

(31) Yudistira, H. T.; Nguyen, V. D.; Dutta, P.; Byun, D. Flight Behavior of Charged Droplets in Electrohydrodynamic Inkjet Printing. *Appl. Phys. Lett.* **2010**, *96*, 023503.

(32) Park, J.-U.; et al. Nanoscale, Electrified Liquid Jets for High-Resolution Printing of Charge. *Nano Lett.* **2010**, *10*, 584–591.

(33) Li, A.; Zi, Y.; Guo, H.; Wang, Z. L.; Fernández, F. M. Triboelectric Nanogenerators for Sensitive Nano-Coulomb Molecular Mass Spectrometry. *Nat. Nanotechnol.* **2017**, *12*, 481–487.

(34) Xu, W.; et al. A Droplet-Based Electricity Generator with High Instantaneous Power Density. *Nature* **2020**, *578*, 392–396.

(35) Lin, Z.-H.; Cheng, G.; Lin, L.; Lee, S.; Wang, Z. L. Water–Solid Surface Contact Electrification and Its Use for Harvesting Liquid-Wave Energy. *Angew. Chem., Int. Ed.* **2013**, *52*, 12545–12549.

(36) Shi, Q.; Wang, H.; Wang, T.; Lee, C. Self-Powered Liquid Triboelectric Microfluidic Sensor for Pressure Sensing and Finger Motion Monitoring Applications. *Nano Energy* **2016**, *30*, 450–459.

(37) Choi, D.; et al. Spontaneous Occurrence of Liquid-Solid Contact Electrification in Nature: Toward a Robust Triboelectric Nanogenerator Inspired by the Natural Lotus Leaf. *Nano Energy* **2017**, *36*, 250–259.

(38) Scholtz, V.; Pazlarova, J.; Souskova, H.; Khun, J.; Julak, J. Nonthermal Plasma—A Tool for Decontamination and Disinfection. *Biotechnol. Adv.* **2015**, *33*, 1108–1119.

(39) Ajo, P.; et al. Hospital Wastewater Treatment with Pilot-Scale Pulsed Corona Discharge for Removal of Pharmaceutical Residues. *J. Environ. Chem. Eng.* **2018**, *6*, 1569–1577.

(40) Zhang, H.; Ma, D.; Qiu, R.; Tang, Y.; Du, C. Non-Thermal Plasma Technology for Organic Contaminated Soil Remediation: A Review. *Chem. Eng. J.* **2017**, *313*, 157–170.

(41) Moad, G.; Solomon, D. H. *The Chemistry of Radical Polymerization*, 2nd ed.; Elsevier Science Ltd.: 2005.

(42) Tietze, L. F. Domino Reactions in Organic Synthesis. *Chem. Rev.* **1996**, *96*, 115–136.

(43) Shen, S.; et al. A Hybrid Nanomaterial for the Controlled Generation of Free Radicals and Oxidative Destruction of Hypoxic Cancer Cells. *Angew. Chem., Int. Ed.* **2017**, *56*, 8801–8804.

(44) Wang, X.-Q.; Wang, W.; Peng, M.; Zhang, X.-Z. Free Radicals for Cancer Theranostics. *Biomaterials* **2021**, *266*, 120474.

(45) Friaa, O.; Brault, D. Kinetics of the Reaction between the Antioxidant Trolox® and the Free Radical DPPH$^{\cdot}$ in Semi-Aqueous Solution. *Org. Biomol. Chem.* **2006**, *4*, 2417–2423.

(46) Zheng, L.; Lin, L.; Su, G.; Zhao, Q.; Zhao, M. Pitfalls of Using 1,1-Diphenyl-2-Picrylhydrazyl (DPPH) Assay to Assess the Radical Scavenging Activity of Peptides: Its Susceptibility to Interference and Low Reactivity Towards Peptides. *Food Res. Int.* **2015**, *76*, 359–365.

(47) Williams, R. L.; Wilson, D. J.; Rhodes, N. P. Stability of Plasma-Treated Silicone Rubber and Its Influence on the Interfacial Aspects of Blood Compatibility. *Biomaterials* **2004**, *25*, 4659–4673.

(48) Nemani, S. K.; et al. Surface Modification of Polymers: Methods and Applications. *Adv. Mater. Interfaces* **2018**, *5*, 1801247.

(49) Zille, A.; Oliveira, F. R.; Souto, A. P. Plasma Treatment in Textile Industry. *Plasma Process. Polym.* **2015**, *12*, 98–131.

(50) Wolf, M. P.; Salieb-Beugelaar, G. B.; Hunziker, P. PDMS with Designer Functionalities—Properties, Modifications Strategies, and Applications. *Prog. Polym. Sci.* **2018**, *83*, 97–134.

(51) Zhou, J.; Khodakov, D. A.; Ellis, A. V.; Voelcker, N. H. Surface Modification for PDMS-Based Microfluidic Devices. *Electrophoresis* **2012**, *33*, 89–104.

(52) McDonald, J. C.; Whitesides, G. M. Poly(Dimethylsiloxane) as a Material for Fabricating Microfluidic Devices. *Acc. Chem. Res.* **2002**, *35*, 491–497.

(53) Zhou, J.; Ellis, A. V.; Voelcker, N. H. Recent Developments in PDMS Surface Modification for Microfluidic Devices. *Electrophoresis* **2010**, *31*, 2–16.

(54) Wong, I.; Ho, C.-M. Surface Molecular Property Modifications for Poly(Dimethylsiloxane) (PDMS)-Based Microfluidic Devices. *Microfluid. Nanofluidics* **2009**, *7*, 291.

(55) Vickers, J. A.; Caulum, M. M.; Henry, C. S. Generation of Hydrophilic Poly(Dimethylsiloxane) for High-Performance Microchip Electrophoresis. *Anal. Chem.* **2006**, *78*, 7446–7452.

(56) Hillborg, H.; Gedde, U. W. Hydrophobicity Recovery of Polydimethylsiloxane after Exposure to Corona Discharges. *Polymer* **1998**, *39*, 1991–1998.

(57) Efimenko, K.; Wallace, W. E.; Genzer, J. Surface Modification of Sylgard-184 Poly(Dimethyl Siloxane) Networks by Ultraviolet and Ultraviolet/Ozone Treatment. *J. Colloid Interface Sci.* **2002**, *254*, 306–315.

(58) Zhang, H.; et al. Fabrication of Robust Hydrogel Coatings on Polydimethylsiloxane Substrates Using Micropillar Anchor Structures with Chemical Surface Modification. *ACS Appl. Mater. Interfaces* **2014**, *6*, 9126–9133.

(59) Trantidou, T.; Elani, Y.; Parsons, E.; Ces, O. Hydrophilic Surface Modification of PDMS for Droplet Microfluidics Using a Simple, Quick, and Robust Method via PVA Deposition. *Microsyst. Nanoeng.* **2017**, *3*, 16091.

(60) He, Q.; et al. Preparation of Hydrophilic Poly(Dimethylsiloxane) Stamps by Plasma-Induced Grafting. *Langmuir* **2003**, *19*, 6982–6986.

(61) Tu, Q.; et al. Antifouling Properties of Poly(Dimethylsiloxane) Surfaces Modified with Quaternized Poly(Dimethylaminoethyl Methacrylate). *Colloids Surf. B* **2013**, *102*, 361–370.

(62) Hillborg, H.; et al. Crosslinked Polydimethylsiloxane Exposed to Oxygen Plasma Studied by Neutron Reflectometry and Other Surface-Specific Techniques. *Polymer* **2000**, *41*, 6851–6863.

(63) Zhu, Y.; Otsubo, M.; Honda, C.; Tanaka, S. Loss and Recovery in Hydrophobicity of Silicone Rubber Exposed to Corona Discharge. *Polym. Degrad. Stab.* **2006**, *91*, 1448–1454.

(64) Li, X.; et al. Polymer Electrets and Their Applications. *J. Appl. Polym. Sci.* **2021**, *138*, 50406.

(65) Bu, L.; Wu, X.; Wang, X.; Liu, L. Silicon-Based Polytetrafluoroethylene Electrets: Preparation and Corona Charging Characteristics. *J. Electrostat.* **2013**, *71*, 666–672.

(66) Zangmeister, C. D.; Radney, J. G.; Vicenzi, E. P.; Weaver, J. L. Filtration Efficiencies of Nanoscale Aerosol by Cloth Mask Materials Used to Slow the Spread of SARS-CoV-2. *ACS Nano* **2020**, *14*, 9188–9200.

(67) Ullah, S.; et al. Reusability Comparison of Melt-Blown vs Nanofiber Face Mask Filters for Use in the Coronavirus Pandemic. *ACS Appl. Nano Mater.* **2020**, *3*, 7231–7241.

(68) Antoniu, A.; Tabti, B.; Plopeanu, M.; Dascalescu, L. Accelerated Discharge of Corona-Charged Nonwoven Fabrics. *IEEE Trans. Ind. Appl.* **2010**, *46*, 1188–1193.

(69) Prawatya, Y. E.; Neagoe, M. B.; Zeghloul, T.; Dascalescu, L. Surface-Electric-Potential Characteristics of Tribo- and Corona-Charged Polymers: A Comparative Study. *IEEE Trans. Ind. Appl.* **2017**, *53*, 2423–2431.

(70) Kowalonek, J.; Kaczmarek, H.; Królikowski, B.; Klimiec, E.; Chylińska, M. Corona Charging of Isotactic-Polypropylene Composites. *Polymers* **2021**, *13*, 942.
