# Supplementary material for: Plug Flow: Generating Renewable Electricity with Water from Nature by Breaking the Limit of Debye Length
Source: ACS Cent Sci. 2025 Apr 16;11(5):719–33. doi: 10.1021/acscentsci.4c02110 (PMC12164936; doi:10.1021/acscentsci.4c02110)
Supplement: Supplementary file 2 [file oc4c02110_si_002.pdf]

oc-2024-02110m.R1

Name: Peer Review Information for "Plug Flow: Generating Renewable Electricity with Water from Nature by Breaking the Limit of Debye Length"

First Round of Reviewer Comments

Reviewer: 1

Comments to the Author

The article presents an innovative approach to harnessing renewable energy from natural water sources. The study focuses on the phenomenon of plug flow, where water falling through a millimeter-sized tube generates electricity with impressive efficiency and power density, surpassing the theoretical limits set by the Debye length in macroscale channels. The plug flow induces a unique interfacial chemistry that enables the complete spatial separation of aqueous H+ and OH- ions, eliminating the electric double layer and allowing for the harnessing of energy from natural water sources such as rain or river water without the need for pumps. The simplicity of the setup and its ability to power multiple LEDs, modify surfaces, and perform chemical reactions highlight its potential for sustainable energy solutions. However, there are still some issues in the article that need to be addressed before accepted:

1 There is still some controversy regarding the preferential adsorption of aqueous OH- ions over aqueous H+ ions onto solid surfaces. Can the author use NMR or XPS to test the surface after flowing through the liquid to determine if there is adsorption of OH-.

2 The author can use COMSOL to simulate the physical processes and differences of several flow modes, so that readers can better understand them

3.Suggest the author to conduct additional experiments to verify the experimental results of plug flow under different environmental conditions, such as different water quality, temperature, and flow rate.

4. Will repeated flow cause the internal surface of the pipeline to be filled with OH- adsorption sites? Will this lead to a decrease in charging efficiency?

5. The author should use a Faraday cup to measure the charge carried on water to demonstrate the experimental results.

Reviewer: 2

Comments to the Author

The manuscript reported a phenomenon that plug flow of water that falls naturally down a millimeter-sized tube generates continuous electricity for about 20 s, yielding a high efficiency of >10% and power density of ~100 W/m2. The generated electricity is attributed to the complete spatial separation of the positive and negative charges at the solid-liquid surface produced by plug flow. The results are interesting. However, some key problems including mechanism analysis of electricity generation in the manuscript are confusing and not convincing with scientific rigor. Thus, this submitted manuscript needs powerful improvements before considering for publication. Here are specific comments:

1. How does the OH- efficiently rise up along the hydrophobic and dielectric surface like PTFE and FEP? Some experimental or theoretical support is needed.

2. Considering the intermittent fluidity of water in plug flow, why the electricity produced by plug flow is continuous? If the plug flow stops, how will the voltage change?

3. Does the prolonged plug flow reduce the charge separating performance of the surface?

4. Do different salt solutions or salt concentrations have a significant effect on output? Considering the practical application, tap water is more common.

5. The review of previously references should be objective. For example, the author claimed that the droplet-based generators are only able to produce transient pulsed power and the highest reported average power density on the order of 0.1 to 1 W/m2. To my knowledge, however, such devices can produce a power density of over 50 W/m2.

Author's Response to Peer Review Comments:

NATIONAL UNIVERSITY OF SINGAPORE

DEPARTMENT OF CHEMICAL AND BIOMOLECULAR ENGINEERING

Siowling, Soh
Associate Professor

4 Engineering Drive 4
Singapore 117585, Singapore
Tel.: (+65) 6516 6617
Fax: (+65) 6779 1936
http://cheed.nus.edu.sg/stf/chessl/
E-mail: chessl@nus.edu.sg

March 7, 2025

To: Professor Nanfeng Zheng
Senior Editor
ACS Central Science

Dear Professor Zheng,

We thank you very much for evaluating our manuscript entitled "**Plug Flow: Generating Renewable Electricity with Water from Nature by Breaking the Limit of Debye Length**" that we submitted to *ACS Central Science.*

We sincerely thank all the Reviewers very much for their very helpful comments. The Reviewers commented that more details should be included in our work, including more material characterization, evaluation of our system under various conditions, and discussions. In this revision, we have addressed all their comments carefully. At the same time, we have modified the manuscript based on the Reviewers' comments, including new experimental results and more discussions. We think that the manuscript is greatly improved after the revision for publication.

In the following section of this letter, we provide our point-by-point replies to the Reviewers' comments. We have now modified the manuscript and highlighted the changes in yellow. Due to the contribution of another group member, Chengyu Zhang, we have added him as an author to this study, as agreed by all the authors involved.

We thank you very much for your consideration.

With Best Regards,

Siowling, Soh

Reviewer #1 (Remarks to the Author):

The article presents an innovative approach to harnessing renewable energy from natural water sources. The study focuses on the phenomenon of plug flow, where water falling through a millimeter-sized tube generates electricity with impressive efficiency and power density, surpassing the theoretical limits set by the Debye length in macroscale channels. The plug flow induces a unique interfacial chemistry that enables the complete spatial separation of aqueous H+ and OH- ions, eliminating the electric double layer and allowing for the harnessing of energy from natural water sources such as rain or river water without the need for pumps. The simplicity of the setup and its ability to power multiple LEDs, modify surfaces, and perform chemical reactions highlight its potential for sustainable energy solutions. However, there are still some issues in the article that need to be addressed before accepted:

Reply: We sincerely thank the Reviewer very much for evaluating this manuscript and the many valuable comments for improving our work.

1 There is still some controversy regarding the preferential adsorption of aqueous OH- ions over aqueous H+ ions onto solid surfaces. Can the author use NMR or XPS to test the surface after flowing through the liquid to determine if there is adsorption of OH-.

Reply: We thank the Reviewer very much for his/her comment. As the Reviewer commented, it is greatly needed to test the surface to determine if there is adsorption of OH^-. In our originally submitted manuscript, we tested the surface of the tube after flowing plug flows of water by FTIR (Figure 5d) and XPS (Figure 5e) to detect OH^-. However, the analyses were very challenging due to the small amount of charge present on the surface. We found from our analyses by FTIR and XPS that there was no difference before and after flowing the plug flow of water.

In response to the Reviewer's comment, we considered using NMR (specifically solid-state NMR) to test the surface but found that it was challenging due to various reasons, including low sensitivity of the technique, bulk analysis (instead of surface analysis), and similar chemical shifts of H_2O and OH^- (thus making it difficult to distinguish if the adsorption was due to the passing water or OH^-).

We are very pleased to mention that we have now obtained clear results that showed that OH^- ions adsorbed onto the surface in response to the comment by the Reviewer. For obtaining these results, we used a sensitive surface-specific technique: the Time-of-Flight Secondary Ion Mass Spectrometry (ToF-SIMS). ToF-SIMS can focus on the topmost layer of the surface and detect surface species down to the concentration of parts per billion. We found previously that this low detection limit was capable of detecting the charged species generated by contact electrification even when there was only a very small amount of charge (e.g., *Nat Commun* **15**, 1986 (2024)). Experimentally, we analyzed the inner surfaces of the FEP tubes by ToF-SIMS before and immediately after flowing plug flows of water or fully continuous flow. After analyzing the surface that contacted the plug flow, we found that a strong peak that represented OH^- intensity at $m/z = 17$ appeared (see the newly added plot II in Figure 5f). On the other hand, the OH^-

intensities of the surfaces before contacting any flowing water ("Dry tube" in plot I, Figure 5f) and after contacting the full continuous flow ("Full flow" in plot III, Figure 5f) were similar and negligible. These results indicated that OH^- ions absorbed onto the inner surface of the FEP tube after flowing plug flow across the tube.

In response to the Reviewer's comment, we have now included the new ToF-SIMS results in the main text, in Figure 5f, and SI. The newly added results are copied here as follows.

Main text:

Because water charged only positively regardless of the type of surface, the charged species generated in the water is probably not related to the surface (e.g., the mobile counterions of the polyelectrolytes) — but the water itself (Figure 5a). Previous studies have reported that aqueous OH^- ions preferentially adsorb over aqueous H^+ ions onto solid surfaces.[34-36] To investigate the role of OH^- ions, we measured the pH of basic (pH 9) aqueous solutions before and after flowing either the discontinuous drips or a continuous stream down an inclined V-shaped PTFE surface (Section S11). Results showed that pH decreased significantly (i.e., ΔpH ~0.3) when the flow was discontinuous, but was negligible when the flow was continuous (Figure 5b). No change in chemical composition of the water was detected by NMR (Figure 5c). Similarly, no change in chemical composition of the surface of the FEP tube before and after flowing water was detected by Fourier-transform infrared spectroscopy (FTIR; Figure 5d) and X-ray photoelectron spectroscopy (XPS; Figure 5e), possibly because the changes were small. For higher sensitivity,[37] we used Time-of-Flight Secondary Ion Mass Spectrometry (ToF-SIMS) to detect the adsorption of OH^- ions on the inner surface of the FEP tube before and immediately after flowing plug flows of water or fully continuous flow. Result of analyzing the surface after contacting the plug flow showed a strong peak that represented OH^- intensity at m/z = 17 appeared ("Plug flow" in plot II, Figure 5f). On the other hand, the OH^- intensities of the surfaces before contacting any flowing water ("Dry tube" in plot I, Figure 5f) and after contacting the full continuous flow ("Full flow" in plot III, Figure 5f) were similar and negligible. These results indicated that OH^- ions absorbed onto the inner surface of the FEP tube after flowing plug flow across the tube.

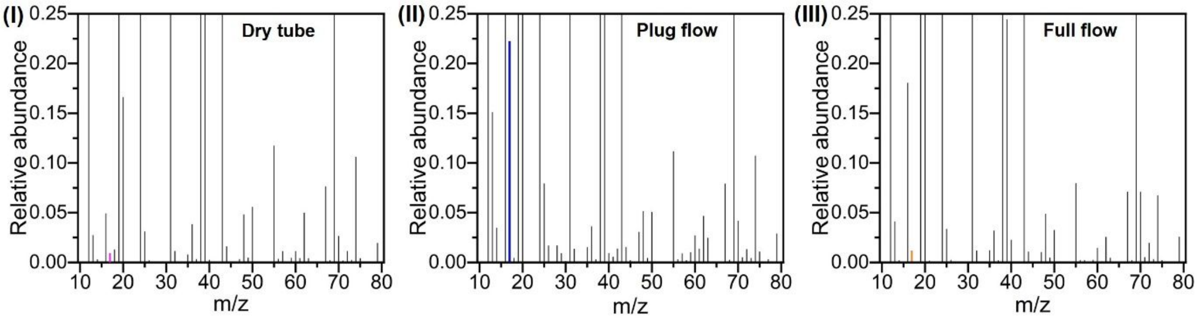

Figure 5. **Discontinuity triggers unique molecular charge separation and charge transport.** **(a)** The unique molecular mechanism of localized charge separation by discontinuous flows. **(b)** Change in pH of solution when it was not flowed across any surface ("Control"), flowed as discontinuous drips across a surface, or flowed as a continuous stream across a surface. **(c)** NMR spectra of the discontinuous drips of water down a FEP tube and discharged water without flowing through any tube ("Control"). **(d)** FTIR and **(e)** XPS survey spectra of the FEP tubes

before (black line) and after (red line) flowing water through them. The insets show the high-resolution spectra of C 1s and F 1s. **(f)** Normalized Time-of-Flight Secondary Ion Mass Spectrometry (ToF-SIMS) spectra of the surfaces of the FEP tubes with the OH^- peak shown at m/z = 17 for (I) before flow ("Dry tube"; red bar), (II) after plug flow ("Plug flow"; blue bar) or (III) after full continuous flow ("Full flow"; brown bar). **(g)** Charges of different portions of a long discrete column of water that flowed through the tube. **(h)** Scheme illustrates the large-scale charge transport by the plug flow that involves the counterflow of the positive and negative charges throughout the tube. All error bars in this figure represent the standard deviation.

SI:

S1. Analyzing the Chemical Composition of the Tube Before and After Flow

We analyzed the chemical composition of the fluorinated ethylene propylene (FEP) tubes that we purchased commercially and used in our experiments for generating power before and after flowing water. The surfaces of the tubes were analyzed by Fourier-transform infrared spectroscopy (FTIR), X-ray photoelectron spectroscopy (XPS), and Time-of-Flight Secondary Ion Mass Spectromety (Tof-SIMS). The FTIR spectra were recorded by a Vertex 70 spectrometer (Bruker, USA) using the Attenuated Total Reflectance (ATR) mode. The XPS spectra were recorded on a Kratos AXIS Ultra^{DLD} system (Kratos Analytical, UK) with Al Kα excitation radiation (1486.71 eV). The pressure in the analysis chamber was maintained at 10^{-6} Pa during the measurement. All spectra were referenced to the C 1s hydrocarbon peak at 285.0 eV to compensate for the effect of surface charging. ToF-SIMS analysis was carried out using a Time-of-Flight Secondary Ion Mass Spectrometer (ToF-SIMS 5 IONTOF) equipped with a Bi_1^+ liquid-metal primary ion source. Primary ion bombardment was done by 30 keV Bi_1^+ ions with a pulsed current of 1 pA. An analysis area of 100 × 100 µm with raster pixels of 128 by 128 was scanned and at least three different spots were analyzed. The total number of cycles for acquisition was fixed at 200. Electron flood gun was used to compensate the positive charging of the FEP sample by the primary ion beam.

Results from the analysis by FTIR showed the characteristic CF_2 symmetric stretching peaks at 1201 and 1146 cm^{-1}, C-CF_3 side chain stretching peak at 982 cm^{-1}, CF deformation peaks at 637 and 627 cm^{-1}, and CF_2 bending peaks at 554 and 511 cm^{-1} (Figure 5d).[1,2] Besides these signals, we did not obtain any other signal (e.g., no C-O stretching bond was detected). XPS detected only C 1s (285 eV, 293 eV) and F 1s (690 eV), together with a negligible amount of O 1s (532 eV) (Figure 5e). The peak at 293 eV from the C 1s spectrum represents the C-F bond. The peak at 690 eV from the F 1s spectrum also represents the C-F bond. The peak at 285 eV from the C 1s spectrum may represent the C-C bond of the material and/or any adventitious carbon on the surface. Quantitative elemental analysis of the surfaces was also performed by XPS. The elemental ratio of C to F is around 1.8 (Table S1); this ratio is commonly reported when solids made of FEP or PTFE are analyzed by XPS.[3,4] Based on the results from all these analyses, we can conclude that the tubes we used for generating power are made of the FEP polymer.

We further determined whether the chemical composition of the tube changed after flowing water through it and drying it. For this analysis, we flowed an extensive amount of water through the tube and kept it in humid condition for 3 days. We then dried the tube with a stream of argon gas for 1 min and analyzed its chemical composition by FTIR and XPS. The results from FTIR and XPS showed that there is no difference between the tubes that had water flowed through them or not.

We performed ToF-SIMS for three cases: before flowing water, after plug flow, and after full continuous flow. For comparing the intensities of the peaks across different spectra from different cases, normalization of the intensities was performed to eliminate the systematic differences generated by ToF-SIMS for each separate analysis.[5] The intensities of the peaks were normalized by the intensity of the peak CF^- (m/z = 31). We considered the intensity of the peak CF^- as the normalization factor because it is the main chemical moiety in FEP. For the normalization, we divided the intensities of all the peaks in the spectrum by the intensity of the peak CF^- for comparing the intensities of the peaks across different analyses.

The results of the analyses showed that the normalized intensity of OH^- on the inner surface of the FEP tube after flowing plug flow was strong. Negligible intensities were found for the surface before flowing water and the surface after full continuous flow. The trend was clear and remained the same using other normalization factors such as F^- (m/z = 19).

2 The author can use COMSOL to simulate the physical processes and differences of several flow modes, so that readers can better understand them

Reply: We sincerely thank the Reviewer for his/her comment. Yes, modeling the physical processes (e.g., via COMSOL) and the different flow modes would enable readers to understand the phenomena better. Our group is in the engineering department; hence, we can perform numerical simulations (including COMSOL simulations) and understand the value of computational simulations. On the other hand, it is technically difficult to model the processes at this initial stage of the technology. The main discovery of this current manuscript is that plug flow pattern greatly affects charge separation at the solid-liquid interface and triggers a new form of chemistry of charge separation. This main point, however, is difficult to model. We considered different simulations with different levels of difficulties. More details are included in the following paragraphs.

We first note that this manuscript reports for the first time a new form of chemistry that allows a superior form of solid-liquid charge separation. Therefore, there are no fundamental equations developed yet for describing this superior form of charge separation. Without equations, simulation cannot be performed. Specifically, we quantified the main point of this study: the superior charge separation at about ~1 nC/mm of the contact line between the receding edge of the plug and the solid surface. Ideally, any theory or simulation should be developed to explain the main point of this study; in this case, a simulation will need to explain the quantity of ~1 nC/mm of superior charge separation. However, because practically nothing else is known about this superior form of charge separation, it is difficult to perform simulations that will produce results that agree with this experimentally determined quantity of ~1 nC/mm.

The only way to perform simulations is to develop the equations ourselves. However, developing the theoretical framework for a newly discovered phenomenon is typically difficult. In our case, we envision that many experiments will first be needed. We will need to conduct many experiments and systematically vary the many parameters of systems to properly characterize the system. Many parameters affect charge separation in our system, including flow pattern, flowrate, length of the discrete column of plug, dimensions of the tube, material of tube, amount of air mixed, etc. For gaining a better understanding of the charge separation, the amount of each

parameter needs to be varied systematically, for most or all the parameters of the system. After the many experiments and sufficient data, we can understand the relationships between charge separation and the parameters of the system. With this understanding, we will be able to develop the theoretical framework. With the theoretical framework, we will be able to perform the simulations. We will then be able to verify the accuracy of the simulations by comparing the results of the simulations with the experimental results. However, this approach is probably out of the scope of this first manuscript that reports the superior form of solid-liquid charge separation. It is usually not expected to include all the experimental results that first describe and prove the fundamental discovery and also develop a rigorous theoretical and modeling framework, all in a single paper.

Another important factor is that a full simulation of the macroscale system (i.e., the whole tube of 32 cm) is technically challenging. A full-scale simulation of our system would possibly involve fluid mechanics, transport of charged species, electrostatic potential, multi-phase flow (i.e., mixture of air and liquid), and moving boundary conditions. One simulation of this scale is likely going to take at least months for an expert in computational simulations.

A simpler form of simulation that we considered is to simulate the processes occurring only inside a single plug of water in the tube, instead of simulating the full system. To do this, we first make use of the experimentally derived value of about ~1 nC/mm in the simulations, as it is difficult to obtain that value via modeling. Through using this experimental value, we can perform simulations of the fluid mechanics, transport of charged species, and electrostatic potential within the discrete column of plug of water. The results of these simulations will include the spatial distribution of the charged species inside the column of water, spatial velocity distribution, and their evolutions with time. However, these results that represent the processes within only a single plug of water are not of particular interest in this study. This study is about the molecular scale superior solid-liquid charge separation (i.e., the molecular chemistry), different flow patterns (i.e., not just plug flow), and macroscale system for generating electricity with high efficiency. Therefore, the quantification of spatial distributions of charge and velocity over time inside each plug is not really of interest to this study. In fact, we tried using COMSOL to simulate the multi-physics of this simpler case. However, even for this simpler case, it seems to require a long time and a longer time than that of a typical review of a manuscript.

In short, to perform rigorous simulations that describe the main point of this study, we will need many more systematic variations of experimental results and proper development of a relatively large-scale simulation with multiple physics involved. This effort will take time and is a direction that we will work on in future studies. However, this effort may possibly be beyond the scope of this manuscript that reports on the first experimental discovery of the phenomenon.

3.Suggest the author to conduct additional experiments to verify the experimental results of plug flow under different environmental conditions, such as different water quality, temperature, and flow rate.

Reply: We thank the Reviewer very much for his/her comment. In response to the Reviewer's comment, we have now measured the power generated by the plug flow under various

conditions, including water quality, temperature, and flow rate. All the experiments were conducted using the same experimental setup that produced the optimal efficiency of the system, except the parameter studied (i.e., water quality, temperature, or flowrate).

To study the influence of flowrate on efficiency, we performed experiments with four different flowrates: 20 mL/min, 40 mL/min, 60 mL/min, and 80 mL/min. We found that more than 90% of the optimal efficiency of the system can be achieved within a large range of flowrates from 40 mL/min to 80 mL/min.

To study the influence of quality of water on efficiency, we performed experiments with different types of liquids, including deionized water (i.e., the typical case used in our study), tap water, and solutions of sodium chloride (NaCl) with a concentration of 0.1 mM or 10 mM. Results showed that the experiments using tap water and NaCl solutions had more than 85% of the optimal efficiency of the system using deionized water. Although adding a small amount of salt reduced efficiency by a bit, adding more salt did not reduce the efficiency further.

To study the influence of temperature on efficiency, we performed experiments with three different temperatures of water: 4°C, 25°C (typical case), and 50°C. These temperatures cover the typical range of temperatures of water in practical settings. Results showed that the effect of different temperatures was not statistically significant. The pairwise p-values from Student's t-tests of 4°C and 25°C, 25°C and 50°C, and 4°C and 50°C were 0.782, 0.487, and 0.507 respectively. These p-values were all much higher than the standard p-value of 0.05. Hence, the efficiencies generated by experiments using water at different temperatures were statistically similar.

These experiments showed that the system provided consistently high efficiency of power generation under different conditions.

In response to the Reviewer's comment, we have now included the new experimental results in the main text, in a new Figure 2c, 2d, 2e, and SI. The newly added results are copied here as follows.

Main text:

We investigated the influences of different parameters of the system on the efficiency of power generation (Section S6). We first varied systematically the length of the tube and found that the voltage and current from both (P1) and (P2) increased linearly with increasing length of the tube until around 32 cm, beyond which they did not increase further. We varied the flowrate and found that the efficiency remained at more than 90% of the optimal efficiency of the system for a large range of flowrates from 40 – 80 mL/min (Figure 2c). The efficiencies generated by tap water, 0.1 mM NaCl solution, and 10 mM NaCl solution were more than 85% of the optimal efficiency of the system via using deionized water (Figure 2d). The efficiencies generated by using water with different temperatures of 4°C, 25°C, and 50°C were found to be statistically similar (Figure 2e). Therefore, it seemed that the efficiency of power generation by the system remained similar under different environmental conditions.

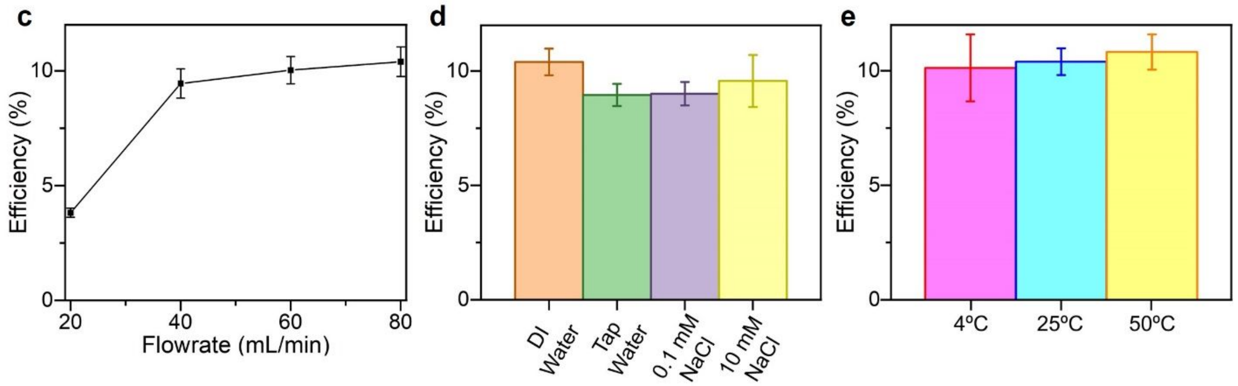

Figure 2. Plug flow provides consistent and highly efficient power generation under different environmental conditions. Efficiencies of power generation were similar when varying **(c)** flowrate (40 – 80 mL/min), **(d)** quality of water, and **(e)** temperature of water. Power was harvested using the optimal setup from (P1) with a resistive load of 61 GΩ and from (P2) with a resistive load of 41 GΩ in parts a-f.

SI:

S6. Highly Efficient Power Generation under Different Environmental Conditions

In the main text, we showed that our system can generate power with an optimal efficiency of 10.4% using a plug flow of deionized water with a flowrate of 80 mL/min through a FEP tube. We investigated the influences of different environmental conditions on efficiency, including flowrate, water quality, and temperature of water.

To study the influence of flowrate on efficiency, we performed experiments with four different flowrates: 20 mL/min, 40 mL/min, 60 mL/min, and 80 mL/min. We found that more than 90% of the optimal efficiency of the system can be achieved within a large range of flowrates from 40 mL/min to 80 mL/min (Figure 2c in the main text).

To study the influence of quality of water on efficiency, we performed experiments with different types of liquids, including deionized water (i.e., the typical case used in our study), tap water, and solutions of sodium chloride (NaCl) with a concentration of 0.1 mM or 10 mM. Results showed that the experiments using tap water and NaCl solutions had more than 85% of the optimal efficiency of the system using deionized water (Figure 2d in the main text). Although adding a small amount of salt reduced efficiency by a bit, adding more salt did not reduce the efficiency further.

To study the influence of temperature on efficiency, we performed experiments with three different temperatures of water: 4°C, 25°C (typical case), and 50°C. These temperatures cover the typical range of temperatures of water in practical settings. Results showed that the effect of different temperatures was not statistically significant (Figure 2e in the main text). The pairwise p-values from Student's t-tests of 4°C and 25°C, 25°C and 50°C, and 4°C and 50°C were 0.782, 0.487, and 0.507 respectively. These p-values were all much higher than the standard p-value of 0.05. Hence, the efficiencies generated by experiments using water at different temperatures were statistically similar.

These experiments showed that the system provided consistently high efficiency of power generation under different conditions.

4. Will repeated flow cause the internal surface of the pipeline to be filled with OH- adsorption sites? Will this lead to a decrease in charging efficiency?

Reply: We thank the Reviewer very much for his/her great comment. Yes, any energy-generating system needs to be able to perform effectively for prolonged periods of time. In response to the Reviewer's comment, we have now conducted two sets of experiments to test whether the system can generate power consistently over long periods of time. In the first experiment, we flowed the plug flows of water through the tube for three durations of time: 1 min, 1 h, or 2 h. Similar efficiencies were obtained at (P1) and (P2); hence, the total efficiencies were similar for all three durations of flow. In the second experiment, we flowed plug flows of water through the same FEP tube five times each day, 1 minute each time, for seven days in a row. We found that the efficiency of the system remained approximately constant throughout all seven days. Therefore, it seems that the system is able to be used repeatedly for long durations of time.

The Reviewer mentioned that the surface may be filled with OH^- after repeated use. Based on the two newly performed experiments, it seems that repeated use is fine. An explanation is that the OH^- ions migrate continuously upward on the surface of the FEP tube and the negative charge flows out of the system continuously at point (P2). Due to the continuous migration of the OH^- ions across the surface and discharge, the surface is not saturated with OH^- adsorption; hence, the system can be used repeatedly for long periods of time. In other words, the system has reached steady state for the production of both positive and negative charges.

In response to the Reviewer's comment, we have now included the new experimental results in the main text, in a new Figure 2f, 2g, and SI. The newly added results are copied here as follows.

Main text:

We found that the system produced consistent power after repeated and long-term use (Section S7). Similar efficiencies were obtained after flowing plug flows of water through the FEP tube at (P1) and (P2) for a duration of either 1 min, 1 h, or 2 h (Figure 2f); hence, the overall efficiencies of the system were similar for all three durations. In another set of experiments, we flowed plug flows of water through the FEP tube five times each day, one minute each time, for seven days in a row (Figure 2g). Similar efficiencies were obtained throughout the seven days.

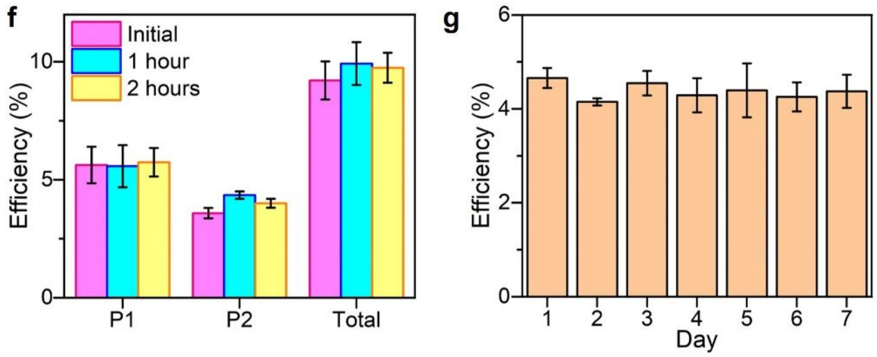

Figure 2. Plug flow provides consistent and highly efficient power generation under different environmental conditions. Long-term consistent power generation as shown by **(f)**

uninterrupted plug flow using a water tower for 1 min (“initial”), 1 h, or 2 h and **(g)** plug flows across seven days. Power was harvested using the optimal setup from (P1) with a resistive load of 61 GΩ and from (P2) with a resistive load of 41 GΩ in parts a-f. For part g, power was harvested only from (P1) with a resistive load of 31 GΩ.

SI:
S7. High Efficiency Maintained After Repeated and Prolonged Use
The ability of an energy-harvesting system to operate consistently for long periods of time is important in practical applications. To test whether our system could perform well after repeated use, we performed two experiments as follows.

The first experiment involved flowing plug flows of water continuously for different durations of 1 min, 1 h, or 2h. The experiment involved using the water tower of 1.65 m. The water tower was filled with water and the water was allowed to enter an FEP tube (inner diameter: 2 mm and length: 32 cm) at a flowrate of 75 mL/min (i.e., same flow rate used in Figure S11). We used a resistive load of 61 GΩ at (P1) and 41 GΩ at (P2). Results showed that the amounts of power generated at (P1) and (P2) were similar for all three durations of flowing water from the water tower (Figure 2f in the main text). Hence, the overall efficiencies of the system combining the power generated at (P1) and (P2) were similar for all durations.

The second experiment involved flowing plug flows of water at a flowrate of 80 mL/min through the same FEP tube (inner diameter: 2 mm and length: 32 cm) five times each day, 1 min each time, for seven days in a row. We used a resistive load of 31 GΩ at (P1) and grounded (P2). After each run, compressed air was blown through the FEP tube for 10 s to remove any stuck water in the tube. Results showed that the efficiency of power generation by the system was consistent throughout the 7 days of test (Figure 2g in the main text).

The results from both experiments showed that the system can consistently produce power with high efficiency after using it repeatedly for long durations of time.

5. The author should use a Faraday cup to measure the charge carried on water to demonstrate the experimental results.

Reply: We thank the Reviewer for his/her comment. We understand that it is important to measure the charge carried on water to show that the electricity generated by our system is due to the separation of charge in the system.

In the originally submitted manuscript, we had measured the charge carried in water as described in Section S12 of the SI. In this experiment, we used only a very small volume of water of 15 μL so that our measurement could be precise. We flowed this small volume of water as a single plug through the FEP tube. We flowed the single plug through the tube at a velocity of 0.6 m/s (i.e., a velocity that is of the same order of magnitude as the typical 80 mL/min that we used in our typical experiments) by compressed air flow. After flowing through the tube, we measured that the charge per mass generated by this small volume of water through the tube was 63.5 nC/g. This charge per mass is similar to that generated by our typical setup that consisted of the continuous stream of plug flow passing through the tube at a flow rate of 80 mL/min at 62 nC/g. Therefore, we verified our experimental results that the electricity generated by our system is due to the charge generated.

In response to the Reviewer's comment, we have now performed another experiment for this revision. Instead of measuring only one single plug of water, we measured the amount of charge generated by a continuous stream of plug flows as performed in a typical experiment in this study. In this case, we measured the total charge generated by 5 mL of deionized water at a flowrate of 80 mL/min through the FEP tube. After flowing through the tube, the water was collected by a Faraday cup, which was connected to an electrometer for measuring the charge of the water. Results showed that the charge per unit mass of water collected in the Faraday cup was measured to be around 60 nC/g. On the other hand, when we used the typical experiment for generating power at the optimal efficiency, we could convert the current generated into charge per unit mass of water at 62 nC/g. Therefore, the charge measured directly by the Faraday cup at 60 nC/g matched the amount generated by the typical experiment for generating optimal power. We thus showed that the power generated was due to the charge generated by the plug flow.

We have now included this new experimental result in the main text and SI. The added text is copied here as follows.

Main text:

We verified that the electricity generated was due to the separation of charge at the solid-liquid interface by directly measuring the amount of charge in the water after flowing out of the FEP tube by a Faraday cup connected to an electrometer. The amount of charge measured by the Faraday cup matched the electricity generated by the system (Section S5).

SI:

S5. Measuring Charge Generated by Plug Flow

To show that the electricity generated by our system was harvested directly from the separation of charge at the solid-liquid interface, we measured the charge generated by the system directly. The experiment involved a plug flow of 5 mL of deionized water through a 2 mm FEP tube at a flowrate of 80 mL/min. After flowing through the tube, the water was collected by a Faraday cup, which was connected to an electrometer for measuring the charge of the water. No external circuit was connected to the water (i.e., (P1)) collected in the Faraday cup. A resistive load of 41 GΩ was used at (P2) (i.e., the same resistive load used at (P2) for generating the optimal efficiency). Results showed that the charge per unit mass of water collected in the Faraday cup was measured to be around 60 nC/g. On the other hand, when we used the typical experiment for generating power at the optimal efficiency, we could convert the current generated into charge per unit mass of water at 62 nC/g. Therefore, the charge measured directly by the Faraday cup at 60 nC/g matched the amount generated by the typical experiment for generating optimal power. We thus showed that the power generated was due to the charge generated by the plug flow.

Reviewer #2 (Remarks to the Author):

The manuscript reported a phenomenon that plug flow of water that falls naturally down a millimeter-sized tube generates continuous electricity for about 20 s, yielding a high efficiency of >10% and power density of ~100 W/m2. The generated electricity is attributed to the complete spatial separation of the positive and negative charges at the solid-liquid surface produced by

plug flow. The results are interesting. However, some key problems including mechanism analysis of electricity generation in the manuscript are confusing and not convincing with scientific rigor. Thus, this submitted manuscript needs powerful improvements before considering for publication. Here are specific comments:

Reply: We sincerely thank the Reviewer very much for his/her many valuable comments for improving the manuscript.

However, there are several points to be clarified and explained more.

1. How does the OH- efficiently rise up along the hydrophobic and dielectric surface like PTFE and FEP? Some experimental or theoretical support is needed.

Reply: We thank the Reviewer very much for his/her comment. Indeed, it may seem difficult for OH^- ions to rise up along a hydrophobic surface like PTFE or FEP. In response to the comment by the Reviewer, we have now performed measurements to provide experimental support for the migration of OH^- ions across a FEP surface. Importantly, the inner surface of the FEP tube was highly moisturized in our system due to the constant flow of water through the tube. The decrease in surface resistivity due to the moisture on the surface enabled the OH^- ions to migrate across the surface. To show this point experimentally, we measured the surface resistivity of a FEP surface using a standard Resistivity Test Fixture (Keithley Model 8009) connected to an electrometer (Keithley Model 6517B). For simulating the moist environment of our system, we moisturized the surface of FEP using a humidifier for 30 seconds. We determined that there was a very large 7 orders of magnitude decrease in surface resistivity from 10^{16} Ω/sq before moisturizing the surface to 10^{9} Ω/sq after moisturizing the surface.

Based on this measured surface resistivity, we calculated the overall surface resistance of the FEP tube. The conversion was performed by multiplying the surface resistivity with the length of the surface (i.e., the length of the FEP tube, 32 cm) and dividing by the width of the surface (i.e., the perimeter of the circle, 6.28 mm). Therefore, the inner wall of the FEP tube has an overall surface resistance of around 10^{11} Ω or around 100 GΩ. This amount of resistance is on the same order of magnitude as the resistive load used in our system (e.g., the typical 61 GΩ or 41 GΩ). Therefore, it is possible that the OH^- ions can migrate across the inner surface of the FEP tube.

The analysis described above is for the case when the surface is exposed to air. On the other hand, when water flows down the tube as plug flows, the inner surface of the tube is exposed alternately to air and water. Water conducts ions readily. For a demonstration, we used an FEP tube of 32 cm (i.e., the tube that we use typically in our experiments) and filled it fully with water. We then measured the bulk resistance of the tube filled with water by attaching an electrode on each end of the tube. The resistance was measured to be about 0.1 GΩ. This resistance is much smaller than the resistive loads used in our typical setup; hence, ions can migrate much faster whenever water is on the surface of the FEP tube.

Previous studies have also determined that water molecules from a humid environment can adsorb onto hydrophobic surfaces such as PTFE and FEP (e.g., *Angew. Chem., Int. Ed.*, **2008**, *47*,

2188–2207. 42; *J. Phys.: Condens. Matter*, **2009**, *21*, 263002). Previous studies have determined that this adsorption of water molecules onto hydrophobic surfaces can increase their surface conductivities (e.g., *RSC Adv.*, **2014**, *4*, 64280–64298; *IEEE Trans. Electr. Insul.*, **1992**, *27*, 909923; *J. Phys. Chem. B*, **2004**, *108*, 20296–20302). Therefore, our measurements are coherent with the understanding from previous studies.

In response to the Reviewer's comment, we have now included the new experimental results performed for this revision of the manuscript in the main text and SI.

Main text:

For this experiment that involved flowing a single column of water through the tube only once, we obtained a high 1.6 nC/mm of charge per unit length of the trailing edge. For the typical plug flow that involved passing through many discrete columns of water (i.e., Figure 3a), the charge per unit length of the trailing edge was 0.8 nC/mm.

We investigated the possibility of migration of ions on the inner surface of the FEP tube. Due to the constant flow of water through the tube, the surface of the tube is expected to be highly moisturized. We measured experimentally that the surface resistivity of a moist FEP surface was 10^9 Ω/sq (Section S13), which corresponds to an overall surface resistance of the inner surface of the tube of 10^{11} Ω. Because the order of magnitude of this resistance is similar to the resistive loads used in the optimal system, it is possible for ions to migrate across the surface of the FEP tube.

Based on all these results, we propose a mechanism by which the plug flow through a tube separates charge effectively at the solid-liquid interface (Figure 5h). A mechanism that is not specific to the type of surface is the separation of H^+ and OH^- ions of water. Water molecules undergo self-ionization to form H^+ and OH^- ions. Previous studies have reported that OH^- ions of water have the tendency to preferentially adsorb over H^+ ions at the solid-liquid interface. The phenomenon is general and occurs for both hydrophilic and hydrophobic surfaces (e.g., polymeric surfaces, vesicles, and self-assembled monolayers on substrates).[36, 38-43] Results from molecular dynamics simulations also showed that OH^- ions tend to adsorb preferentially (i.e., more than the hydronium ions) at interface of water and solid.[34-36, 44] H^+ ions, on the other hand, are known to have superior mobility in aqueous media compared to other types of ions.[45-50]

SI:

S13. Surface Conduction of OH^- Ions Up the FEP Tube

In the main text, we proposed a mechanism in which there is charge separation at the receding edge of the plug of water; the separated H^+ ions follow the water down the tube, whereas the OH^- ions migrate up the surface of the inner wall of the tube. We measured a continuous negative current at the top of the tube (P2) with a power generated that is almost similar to the power generated by the positive current at the bottom of the tube at (P1). We also determined that the tube was negatively charged after the plug flow stopped and the tube did not contain any water in it. These results indicated that the negatively charged ions were generated on the surface of the tube and then migrated upward to the top of the tube.

In general, migration of ions on dry hydrophobic surfaces is difficult and slow. On the other hand, our system involves an extremely high amount of moisture due to the constant flow of water through the tube. Previous studies have determined that water molecules from a humid environment can adsorb onto hydrophobic surfaces such as PTFE and FEP.[22, 23] Previous studies

have also determined that this adsorption of water molecules onto hydrophobic surfaces can increase their surface conductivities.[24-26]

We showed experimentally that the surface conductivity of FEP greatly reduced in a humid environment, thus facilitating the migration of ions on the surface. To show this point experimentally, we measured the surface resistivity of FEP surfaces using a standard Resistivity Test Fixture (Keithley Model 8009) connected to an electrometer (Keithley Model 6517B). To simulate the humid environment, we placed FEP sheets 15 cm on top of the outlet of a commercial humidifier and operated the humidifier for 30 s. The surface conductivity of the moist FEP sheet was then measured immediately. The analysis showed that the surface resistivity of the moist FEP sheet was on the order of 10^9 Ω/sq. The unit Ω/sq refers to the surface resistance of a surface with an aspect ratio of one (i.e., the same length and width). The surface resistivity of the original piece of FEP sheet before moisturizing it with the humidifier was on the order of 10^{16} Ω/sq. Therefore, the surface resistivity decreased by 7 orders of magnitude when the surface of FEP was moist.

Based on the measured surface resistivity, we calculated the overall surface resistance of the FEP tube. The conversion was performed by multiplying the surface resistivity with the length of the surface (i.e., the length of the FEP tube, 32 cm) and dividing by the width of the surface (i.e., the perimeter of the circle, 6.28 mm). Therefore, the inner wall of the FEP tube has an overall surface resistance of around 100 GΩ. This amount of resistance is on the same order of magnitude as the resistive load used in our system (e.g., the typical 60 GΩ or 40 GΩ). Therefore, it is possible for OH^- ions to migrate across the inner surface of the FEP tube.

The analysis described above is for the case when the surface is exposed to air. On the other hand, when water flows down the tube as plug flows, the inner surface of the tube is exposed alternately to air and water. Water conducts ions readily. For a demonstration, we used an FEP tube of 32 cm (i.e., the tube that we use typically in our experiments) and filled it fully with water. We then measured the bulk resistance of the tube filled with water by attaching an electrode on each end of the tube. The resistance was measured to be about 0.1 GΩ. This resistance is much smaller than the resistive loads used in our typical setup; hence, ions can migrate much faster whenever water is on the surface of the FEP tube.

2. Considering the intermittent fluidity of water in plug flow, why the electricity produced by plug flow is continuous? If the plug flow stops, how will the voltage change?

Reply: We thank the Reviewer very much for this excellent comment. Yes, it is important to understand how the intermittent flow of water produces a continuous flow of electricity.

We first reply to the insightful comment by the Reviewer about how the voltage changes when the plug flow stops. In response to this comment by the Reviewer, we have now performed this experiment using our typical setup. In this experiment, we measured and monitored the voltage before and after the water stopped flowing in the system. After the water flow stopped (i.e., time, $t = 0$), we found that the electricity continued to flow and did not stop instantaneously (see the newly added Figure 2a and 2b). The electricity flowed as long as around the order of 1 s after the flow stopped.

For comparison, we determined that the average length of each column of air within the plug flow in the tube was around 5 mm and the velocity of flow in the tube was around 0.35 m/s. Hence, the column of air needed only around ~0.015 s to flow out of the tube. This time is far shorter than the continued flow of electricity even after the flow of water stopped. Therefore, it is possible to supply electricity continuously even when the plug flow consists of air columns.

Fundamentally, we found that the reason why the electricity flowed even though the water flow stopped is because of the high resistive loads that we used at (P1) and (P2). When we used a lower resistive load at (P1), we found that the electricity stopped flowing sooner after the water flow stopped. When no resistors were connected at (P1) and (P2) (i.e., both (P1) and (P2) were connected directly to ground), the flow of electricity stopped instantaneously at <0.1 s after the water stopped flowing in the system. These results indicated that due to the high resistive loads used, charge remained in the system and did not flow out immediately. When the water stopped flowing, the charge that remained in the system then gradually flowed out and caused electricity to continue to flow. The supply of electricity from the system fluctuated with time possibly due to this phenomenon but remained continuous throughout time.

We further provide an order-of-magnitude analysis of the amount of charge remaining in the system that drives the continuous flow of electricity even when air was flowing out of the tube instead of charged water. We first assume that the rate of charge generated by our system per unit time is constant at steady state (i.e., due to the same chemistry of charge separation across a constant length of the tube over time). For this analysis, we performed experiments and found that there was more charge per unit time that flowed out of the system when the resistive load was reduced. At (P1), when the resistive load was 61 GΩ, the rate of charge that flowed out of the system was ~70 nC/s. When the resistive load was only 1 GΩ, the rate of charge that flowed out of the system was ~110 nC/s. If we consider 1 GΩ to be negligible (i.e., relative to the optimal resistance of 61 GΩ) for this discussion, we could conclude from this experimental result that the system inherently was able to generate charge at a rate of 110 nC/s. On the other hand, we only obtained ~70 nC/s when a high resistive load was used. Therefore, based on our assumption of constant rate of charge generation in the system, the difference of around 40 nC/s is the rate of charge that remained in the system due to the high resistive load.

We compare this rate of charge that remained in the system with the rate of charge needed to continuously supply electricity even when air is flowing out of the system instead of charged water. Each column of air flowed out of the tube with a short duration of ~0.015 s. There were roughly on average 15 columns of air in one second. Therefore, there was around 0.2 s of air flowing out of the system per second. The rate of charge typically flowing out of the system with the optimal high resistive load was ~70 nC/s. Hence, the rate of charge needed to continuously supply electricity at the same rate was around 15 nC/s. Compared to the 40 nC/s remained in the system, there is thus sufficient amount of charge in the system for supplying electricity continuously.

In response to the Reviewer's excellent comment, we have now included the results from the newly performed experiments in the main text and SI. These additions are included in this reply as follows.

Main text:

The results showed that the power generated by the system fluctuated with time but remained continuous throughout time (i.e., instead of pulsed instantaneous power). Hence, the system is able to provide continuous power without interruptions. On the other hand, the plug flow was discontinuous and consisted of columns of air in between charged water. To study the continuous generation of power, we stopped the flow of water in the system and monitored the power generation. We found that electricity continued to flow for roughly ~1 s even after the flow stopped (Figure 2a, b). This duration of continued supply of electricity without the flow of charged water was much longer than the duration taken by a single column of air to flow out of the tube at around ~0.015 s. Therefore, electricity could be supplied continuously even when air was flowing out. The reason for the continued supply of electricity was possibly due to accumulated charge within the system that flowed out whenever air was flowing out instead of water (see Section S4 for a more detailed discussion).

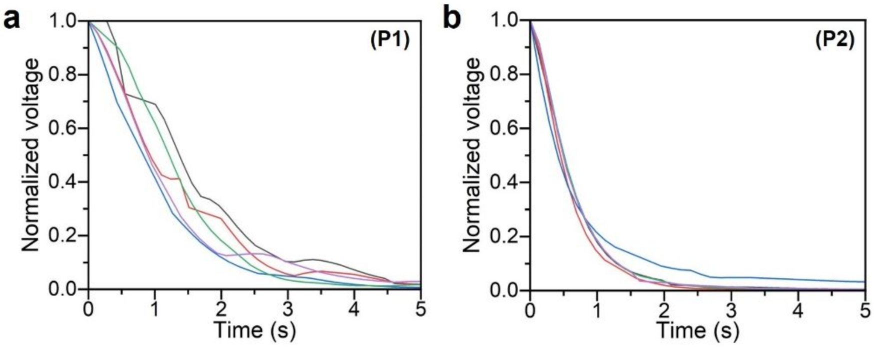

Figure 2. **Plug flow provides consistent and highly efficient power generation under different environmental conditions.** Continuous power generated as determined by decays of voltage after stopping plug flow measured at **(a)** (P1) and **(b)** (P2). Power was harvested using the optimal setup from (P1) with a resistive load of 61 GΩ and from (P2) with a resistive load of 41 GΩ in parts a-f.

SI:

S4. Mechanism of Continuous Power Generation by Discontinuous Plug Flow

In the main text, we described that the power generated from our system was continuous. We measured experimentally that the electricity produced was continuous with time without any interruptions (i.e., breaks with no current). On the other hand, the flow pattern (i.e., plug flow) was discontinuous. In this section, we studied the reason for the production of continuous power from a discontinuous flow pattern.

When charged water flows out of the tube, we obtain electricity. However, plug flow consists of alternating short columns of water and air. When it is time for air to flow out of the tube, we probably should not get any electricity. We calculated the average duration of air that flows out of the tube in between two charged columns of water. After quantifying the plug flow in our system experimentally, we found that the average height of a single column of air in the tube was around 5 mm. The velocity of flow inside the tube (for both water and air) was around 0.35 m/s. Therefore, we calculated that the duration of air that flows out of the tube per air gap in between two columns of water is ~0.015 s. This is a very short time of air flowing out during which there should be no electricity generated.

Subsequently, we examined the flow of electricity after we stopped the plug flow. In this experiment, we used the typical setup for obtaining the optimal electricity, including having a resistive load of 61 GΩ at (P1) and 41 GΩ at (P2). We flowed a finite amount of water (i.e., 50 mL in this case) through the tube until there was no more water. At the same time, we measured and monitored the electricity generated continuously. Our results showed that when the water stopped flowing in the system, the generation of electricity did not stop instantaneously; instead, we found that the electricity decreased gradually with time (see Figure 2a for (P1) and Figure 2b for (P2) in the main text). The results showed that electricity continued to flow even after a relatively long time, on the order of magnitude of ~1 s. This time is relatively much longer than the time when air flows out of the tube and no electricity is generated (i.e., ~0.015 s). Therefore, the electricity can be continuously supplied.

To gain a fundamental understanding of the continuous power, we examined why the electricity did not stop flowing even after water stopped flowing out of the tube for ~1 s. Based on our experimental results, it seemed that it was due to the high resistive loads at (P1) and (P2) that allowed charge to remain in the system for some time before flowing out of the system. To test this mechanism, we investigated different amounts of resistive loads at (P1) and (P2). Our experimental results showed that the duration that electricity continued to flow decreased with decreasing resistive loads. When no resistors were connected at (P1) and (P2) (i.e., both (P1) and (P2) were connected directly to ground), the flow of electricity stopped instantaneous at <0.1 s after the water stopped flowing in the system. These results indicated that due to the high resistive loads used, charge remained in the system and did not flow out immediately. When the water stopped flowing, the charge that remained in the system then gradually flowed out and caused electricity to continue to flow. The supply of electricity from the system fluctuated with time possibly due to this phenomenon but remained continuous throughout time.

We further provide an order-of-magnitude analysis of the amount of charge remaining in the system that drives the continuous flow of electricity even when air was flowing out of the tube instead of charged water. We first assume that the rate of charge generated by our system per unit time is constant at steady state (i.e., due to the same chemistry of charge separation across a constant length of the tube over time). For this analysis, we performed experiments and found that there was more charge per unit time that flowed out of the system when the resistive load was reduced. At (P1), when the resistive load was 61 GΩ, the rate of charge that flowed out of the system was ~70 nC/s. When the resistive load was only 1 GΩ, the rate of charge that flowed out of the system was ~110 nC/s. If we consider 1 GΩ to be negligible (i.e., relative to the optimal resistance of 61 GΩ) for this discussion, we could conclude from this experimental result that the system inherently was able to generate charge at a rate of 110 nC/s. On the other hand, we only obtained ~70 nC/s when a high resistive load was used. Therefore, based on our assumption of constant rate of charge generation in the system, the difference of around 40 nC/s is the rate of charge that remained in the system due to the high resistive load.

We compare this rate of charge that remained in the system with the rate of charge needed to continuously supply electricity even when air is flowing out of the system instead of charged water. Each column of air flowed out of the tube with a short duration of ~0.015 s. There were roughly on average 15 columns of air in one second. Therefore, there was around 0.2 s of air flowing out of the system per second. The rate of charge typically flowing out of the system with the optimal high resistive load was ~70 nC/s. Hence, the rate of charge needed to continuously supply electricity at the same rate was around 15 nC/s. Compared to the 40 nC/s

remained in the system, there is thus sufficient amount of charge in the system for supplying electricity continuously.

3. Does the prolonged plug flow reduce the charge separating performance of the surface?

Reply: We thank the Reviewer very much for his/her great comment. Yes, any energy-generating system needs to be able to perform effectively for prolonged periods of time. In response to the Reviewer's comment, we have now conducted two sets of experiments to test whether the system can generate power consistently over long periods of time. In the first experiment, we flowed the plug flows of water through the tube for three durations of time: 1 min, 1 h, or 2 h. Similar efficiencies were obtained at (P1) and (P2); hence, the total efficiencies were similar for all three durations of flow. In the second experiment, we flowed plug flows of water through the same FEP tube five times each day, 1 minute each time, for seven days in a row. We found that the efficiency of the system remained approximately constant throughout all seven days. Therefore, it seems that the system is able to be used repeatedly for long durations of time.

In response to the Reviewer's comment, we have now included the new experimental results in the main text, in a new Figure 2f, 2g, and SI. The details of the experiments were included. The newly added results are copied here as follows.

Main text:

We found that the system produced consistent power after repeated and long-term use (Section S7). Similar efficiencies were obtained after flowing plug flows of water through the FEP tube at (P1) and (P2) for a duration of either 1 min, 1 h, or 2 h (Figure 2f); hence, the overall efficiencies of the system were similar for all three durations. In another set of experiments, we flowed plug flows of water through the FEP tube five times each day, one minute each time, for seven days in a row (Figure 2g). Similar efficiencies were obtained throughout the seven days.

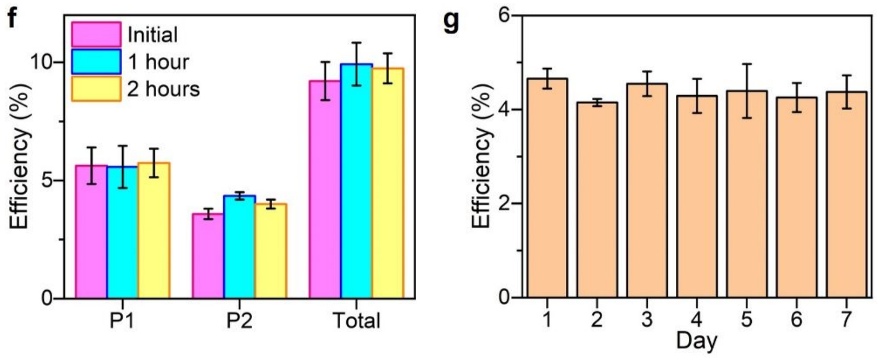

Figure 2. **Plug flow provides consistent and highly efficient power generation under different environmental conditions.** Long-term consistent power generation as shown by **(f)** uninterrupted plug flow using a water tower for 1 min ("initial"), 1 h, or 2 h and **(g)** plug flows across seven days. Power was harvested using the optimal setup from (P1) with a resistive load of 61 GΩ and from (P2) with a resistive load of 41 GΩ in parts a-f. For part g, power was harvested only from (P1) with a resistive load of 31 GΩ.

SI:

S7. High Efficiency Maintained After Repeated and Prolonged Use

The ability of an energy-harvesting system to operate consistently for long periods of time is important in practical applications. To test whether our system could perform well after repeated use, we performed two experiments as follows.

The first experiment involved flowing plug flows of water continuously for different durations of 1 min, 1 h, or 2h. The experiment involved using the water tower of 1.65 m. The water tower was filled with water and the water was allowed to enter an FEP tube (inner diameter: 2 mm and length: 32 cm) at a flowrate of 75 mL/min (i.e., same flow rate used in Figure S11). We used a resistive load of 61 GΩ at (P1) and 41 GΩ at (P2). Results showed that the amounts of power generated at (P1) and (P2) were similar for all three durations of flowing water from the water tower (Figure 2f in the main text). Hence, the overall efficiencies of the system combining the power generated at (P1) and (P2) were similar for all durations.

The second experiment involved flowing plug flows of water at a flowrate of 80 mL/min through the same FEP tube (inner diameter: 2 mm and length: 32 cm) five times each day, 1 min each time, for seven days in a row. We used a resistive load of 31 GΩ at (P1) and grounded (P2). After each run, compressed air was blown through the FEP tube for 10 s to remove any stuck water in the tube. Results showed that the efficiency of power generation by the system was consistent throughout the 7 days of test (Figure 2g in the main text).

The results from both experiments showed that the system can consistently produce power with high efficiency after using it repeatedly for long durations of time.

4. Do different salt solutions or salt concentrations have a significant effect on output? Considering the practical application, tap water is more common.

Reply: We again thank the Reviewer for the great comment. It is important to investigate different types of aqueous solutions to broaden the applicability of our system. In response to the Reviewer's comment, we have now performed experiments with tap water, a salt solution of NaCl with a low concentration of 0.1 mM, and a salt solution of NaCl with a relatively much higher concentration of 10 mM. Our results showed that the experiments using tap water and NaCl solutions had more than 85% of the optimal efficiency that we obtained in this study (i.e., using DI water). These results thus showed that adding a small amount of salt reduced efficiency by a bit but adding more salt does not reduce efficiency further.

In response to the Reviewer's comment, we have now included the new experimental results in the main text, in a new Figure 2d, and SI. The newly added results are copied here as follows.

Main text:

The efficiencies generated by tap water, 0.1 mM NaCl solution, and 10 mM NaCl solution were more than 85% of the optimal efficiency of the system via using deionized water (Figure 2d).

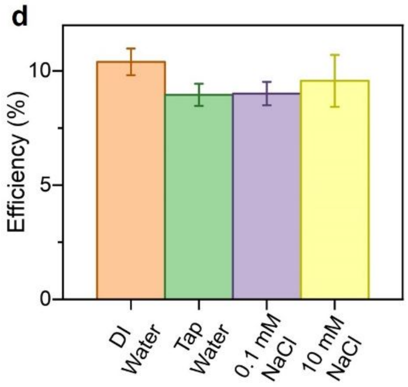

Figure 2. Plug flow provides consistent and highly efficient power generation under different environmental conditions. Efficiencies of power generation were similar when varying **(d)** quality of water. Power was harvested using the optimal setup from (P1) with a resistive load of 61 GΩ and from (P2) with a resistive load of 41 GΩ in parts a-f.

SI:

S6. Highly Efficient Power Generation under Different Environmental Conditions

To study the influence of quality of water on efficiency, we performed experiments with different types of liquids, including deionized water (i.e., the typical case used in our study), tap water, and solutions of sodium chloride (NaCl) with a concentration of 0.1 mM or 10 mM. Results showed that the experiments using tap water and NaCl solutions had more than 85% of the optimal efficiency of the system using deionized water (Figure 2d in the main text). Although adding a small amount of salt reduced efficiency by a bit, adding more salt did not reduce the efficiency further.

5. The review of previously references should be objective. For example, the author claimed that the droplet-based generators are only able to produce transient pulsed power and the highest reported average power density on the order of 0.1 to 1 W/m2. To my knowledge, however, such devices can produce a power density of over 50 W/m2.

Reply: We thank the Reviewer very much for his/her comment. In a very good paper on droplet-based generators, we found that the authors reported a high power density of 50.1 W/m^2 (*Nature* **2020**, *578(7795)*, 392-396). Because this paper is possibly the most well-known and has the same power density of 50 W/m^2 that the Reviewer mentioned, we believe that this is the paper that the Reviewer is referring to. However, the power density of 50.1 W/m^2 that the authors reported is the peak instantaneous power (i.e., or pulsed power) and not average continuous power density. In our study, we focus on average continuous power and not peak instantaneous power. So, we calculated the average continuous power densities of the previously published papers on droplet-based generators and included these values in our introduction, instead of using directly the peak instantaneous power densities reported by the authors. We should have provided more information in our description in the introduction of our manuscript to clarify this point. More details are included in the following paragraphs.

We first mention that peak instantaneous power density is typically far larger than average continuous power density because it refers to the highest achievable power throughout the entire

time (i.e., as opposed to the average power obtained over time). Our system produces continuous power. The average continuous power is usually a much more important quantity for a power source compared to instantaneous power. Therefore, we think that it is reasonable to compare the continuous power density of our system with the continuous power densities of other technologies on the same basis.

Therefore, to compare with our system, we calculated the average continuous power densities of the published works on droplet-based generators based solely on the information provided by the authors in the respective published manuscripts. We first discuss the droplet-based generator reported in the paper *Nature* **2020**, *578(7795)*, 392-396. The authors reported a peak instantaneous power density of 50.1 W/m^2. The authors also reported in this paper that the energy harvested from a single droplet was 3.2 μJ and the frequency of droplet impingement was 4.2 Hz. Therefore, the total energy harvested per second was 13.4 μJ. Because the droplet spreading area was 2.72 cm^2 as reported by the authors, the average continuous power density from the electricity generator in this study was 0.05 W/m^2. This analysis showed the huge difference between peak instantaneous power density and average continuous power density by a large 3 orders of magnitude.

The fundamental reason for the huge difference between peak instantaneous power density and average continuous power density as reported in these droplet-based generators is illustrated in the two figures from two representative articles that we provided below this paragraph: **Representative article 1** (citation: *Nature* **2020**, *578(7795)*, 392-396) and **Representative article 2** (citation: *Droplet* **2024**, *3(1)*, e91). In these two figures from their manuscripts, it is clear that the difference is huge. When the authors refer to the peak instantaneous power (e.g., 50.1 W/m^2), they are referring only to the highest point of the peak. But as we can see from the plots, the highest point on the peak is far from being the representative power continuously provided and averaged across the entire duration. The instantaneous power occurs only for a very short time and drops off rapidly to negligible power. In general, the duration of each instantaneous power is around the range of 0.001 s to 0.0001 s for all the previously published papers on droplet-based generators, including *Nature* **2020**, *578(7795)*, 392-396, *Phys. Rev. Lett.* **2020**, *125* (7), 078301, *EcoMat* **2021**, *3* (4), e12116, *Energy Environ. Sci.* **2022**, *15* (7), 2916-2926, *Droplet* **2022**, *1*, 56-64, *Droplet* **2024**, *3(1)*, e91, *Nano Energy* **2023**, *116*, 108831, *Nano Energy* **2023**, *106*, 108111, *Adv. Mater. Technol.* **2025**, 2401870. The plot in Representative article 1 shows that the duration of the instantaneous power lasts only around 0.001 s. The plot in Representative article 2 shows another typical example in which the instantaneous power lasts around only 0.0001 s. These are very short durations of time. Even within this duration of each instantaneous power, most of the power is still far less than the highest point of the peak.

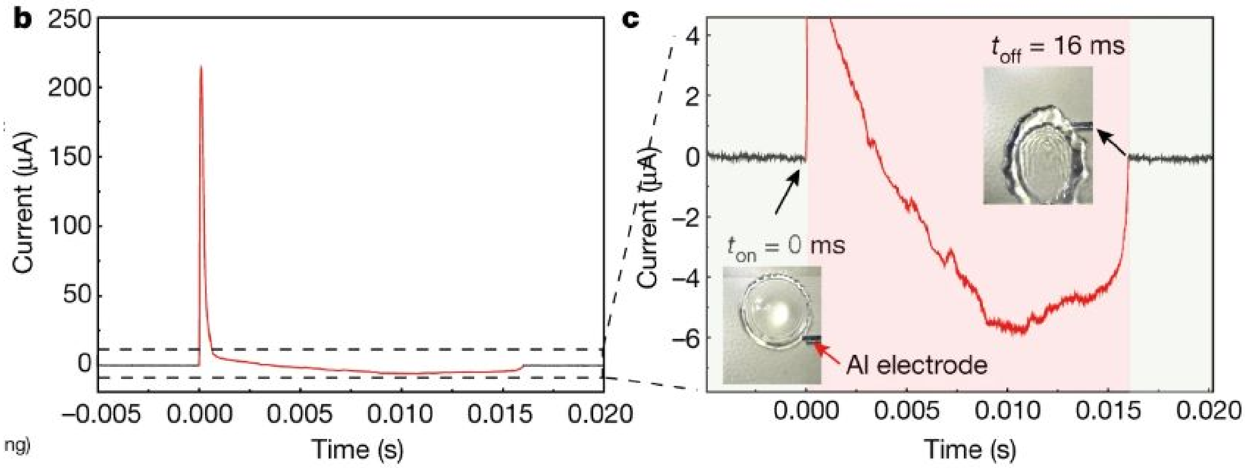

Representative article 1 (citation: *Nature* **2020**, *578(7795)*, 392-396): In this study, a droplet-based electricity generator is fabricated from PTFE. One electrode is placed partially on top of the layer of PTFE. Another electrode is placed at the bottom of the layer of PTFE. First, the water droplet comes into contact with the PTFE surface; it collides and spreads on the PTFE surface. Subsequently, there is detachment of the water droplet after the water leaves the surface on the upper electrode. Instantaneous power is generated by electrostatic induction due to the contact and detachment of the water droplet. The peak power density was found to be 50.1 W/m^2 when using deionized water. The duration of the instantaneous power as shown in the left plot lasts around 0.001 second per drop.

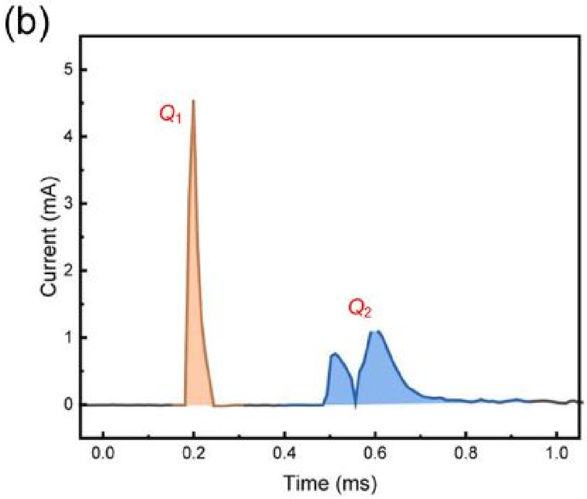

Representative article 2 (citation: *Droplet* **2024**, *3(1)*, e91): In this study, a droplet-based electricity generator made of FEP is combined with another electricity generator, the Kelvin water dropper. As discussed in the figure caption of representative article 1, instantaneous power is generated by electrostatic induction due to the contact and detachment of the water droplet (as the devices are similar). The Kelvin water dropper injects charge onto the FEP surface and leads to an increase in power generation. The duration of the instantaneous power as shown in peak “Q1” lasts less than 0.0001 second per drop.

In the subsequent paragraphs, we will provide a detailed discussion of all the other previously published papers on droplet-based generators, in addition to the discussion of the well-known paper *Nature* **2020**, *578(7795)*, 392-396 in the previous paragraph.

In subsequent studies by the same researchers, they further extended and optimized their system and were able to obtain higher peak instantaneous power than that reported in *Nature* **2020**, *578(7795)*, 392-396. However, after calculating the average continuous power density from the information provided by the manuscripts, the results are always low. For example, in *Droplet* **2022**, *1*, 56-64, the authors reported a higher peak instantaneous power density of 2030 W/m^2 by using concentrated NaCl solutions. However, the average continuous power density that we calculated was only 0.018 W/m^2, which is lower than that reported in *Nature* **2020**, *578(7795)*, 392-396. The reason is fundamentally the same: the very short duration of the instantaneous power as discussed in the previous paragraph and the two figures above.

The paper on droplet-based generators that reported the highest average continuous power density is *EcoMat* **2021**, *3* (4), e12116. In this study, the authors addressed the fundamental limitations of the short durations of the instantaneous power by using a high frequency of droplet impingement. The authors increased the frequency of impingement to the order of 100 Hz by using smaller droplets of volumes at 4 μL and a faster speed of the droplets at 1.85 m/s. With these changes, they obtained the highest average continuous power density of 0.51 W/m^2 with the frequency of droplet impingement at 165 Hz. To the best of our knowledge, this is the highest average continuous power density reported in all previously published papers on standalone droplet-based electricity generators (i.e., based only on the droplet-based generators and not combined with other systems such as solar panels). However, the amount is still small; our system produces around 2 to 3 orders of magnitude more average continuous power density than that reported in this paper, which is the highest reported in droplet-based generators.

In addition, there are some efforts to combine the droplet-based electricity generators with other energy-generating systems. For example, one study reported combining the droplet-based electricity generator with solar panels to increase the power density (*Nano Energy* **2023**, *116*, 108831). However, the scope of our study only involves water energy (e.g., rain or rivers), and not completely different forms of energy such as solar. Hence, we think that it is not necessary for us to compare with solar power or solar panels. Another study involves combining the droplet-based electricity generator with the Kelvin water dropper and reported a high peak instantaneous power density of 10^5 W/m^2 (*Droplet* **2024**, *3(1)*, e91). However, the authors took into account only the surface area of the small droplet-based electricity generator, and not the Kelvin water dropper, for calculating the power density. According to their manuscript, the surface area of the droplet-based electricity generator is small and much smaller than the Kelvin water dropper. Hence, it seems unfair to compare the results of this study. In general, we think that we do not need to compare our results with those droplet-based generators combined with other energy-generating systems.

In conclusion, the papers on droplet-based electricity generators typically report the peak instantaneous power densities and not average continuous power densities. Peak instantaneous power is always far larger than average continuous power for the droplet-based electricity generators. Therefore, we need to calculate and report the much lower value of the average

continuous power density of the droplet-based electricity generators to have a fair comparison with the average continuous power density of our system. Importantly, we found that the highest average continuous power density of the droplet-based electricity generators reported is 0.51 W/m^2, excluding those studies that involve the combination of other energy-harvesting systems (e.g., solar panel). Therefore, we mentioned in our manuscript that for the droplet-based generators, "*the highest reported average power density on the order of 0.1 to 1 W/m^2.*"

In response to the Reviewer's comment, we have now included a more in-depth discussion in the introduction of the manuscript to clarify the point that we make. We have also added more citations of the references that we considered. The modified introduction is included here as follows.

Main text:

Previous research works have thus relied on other fundamental mechanisms instead. One method involves electrostatic induction. Technologies that use electrostatic induction include the Kelvin water dropper and droplet-based electricity generators.[20-33] These systems typically involve first charging a solid surface and then flowing water droplets through or over the charged surface. Some studies reported first pre-charging the surface by either ion injection (i.e., a step that requires energy input) or charging very gradually via solid-liquid charge separation (i.e., a process that requires many droplets and a long time of charging).[22-24, 28] After charging the surface, water droplets are flowed across the charged surface. An electrode underneath the surface senses the changing amounts of charge via repeatedly having droplets on the charged surface or not by electrostatic induction for generating electricity. These devices are only able to produce transient pulsed (i.e., instantaneous but not continuous) power. Although the peak instantaneous powers of these devices are high, the duration of each instantaneous power was very short and ranged typically from 0.1 ms to 1 ms. To understand the effectiveness of these devices as a power source, it is needed to determine the average continuous power density instead of the reported peak instantaneous power. The average continuous power density can be determined by taking into account the energy harvested from one droplet of water, the frequency of droplets contacting the surface, and the surface area of the device. The highest average continuous power density of these devices was only on the order of 0.1 to 1 W/m^2 (i.e., excluding devices combined with other energy-generating systems such as solar panels).[22, 24, 25, 28] Importantly, electricity is generated by electrostatic induction of the pre-existing and steady amount of charge present on the surface — the charge on the surface is not directly harvested as electricity. Because streaming current harvests the constantly separated charge directly as electricity, the mechanisms of these droplet-based generators are fundamentally different from streaming current.

oc-2024-02110m.R2

Name: Peer Review Information for "Plug Flow: Generating Renewable Electricity with Water from Nature by Breaking the Limit of Debye Length"

Second Round of Reviewer Comments

Reviewer: 1

Comments to the Author

All the questions are addressed. The paper can be accepted.

Reviewer: 2

Comments to the Author

The authors have made carefully corrections and addressed my concerns. I support the publication of this work.

Author's Response to Peer Review Comments:

NATIONAL UNIVERSITY OF SINGAPORE

DEPARTMENT OF CHEMICAL AND BIOMOLECULAR ENGINEERING

Siowling, Soh

Associate Professor 4 Engineering Drive 4

Singapore 117585, Singapore

Tel.: (+65) 6516 6617

Fax: (+65) 6779 1936

http://cheed.nus.edu.sg/stf/chessl/

E-mail: chessl@nus.edu.sg

April 1, 2025

Senior Editor

ACS Central Science

Dear Editor,

We thank you very much for evaluating our manuscript entitled “Plug Flow: Generating Renewable Electricity with Water from Nature by Breaking the Limit of Debye Length” that we submitted to ACS Central Science. We sincerely thank all the Reviewers very much for their very helpful comments.

We thank you very much for your consideration.

With Best Regards,

Siowling, Soh

Reviewer #1 (Remarks to the Author):

All the questions are addressed. The paper can be accepted.

Reply: We sincerely thank the Reviewer very much for evaluating this manuscript and the many valuable comments for improving our work.

Reviewer #2 (Remarks to the Author):

The authors have made carefully corrections and addressed my concerns. I support the publication of this work.

Reply: We sincerely thank the Reviewer very much for his/her many valuable comments for improving the manuscript.
